# Supplementary material for: Financial burden of HIV and TB among patients in Ethiopia: a cross-sectional survey
Source: BMJ Open. 2020 Jun 1;10(6):e036892. doi: 10.1136/bmjopen-2020-036892 (PMC7265036; doi:10.1136/bmjopen-2020-036892)
Supplement: Supplementary data [file bmjopen-2020-036892supp001.pdf]

**Supplementary appendix 1**

# The financial burden of HIV and TB among patients in Ethiopia: a cross-sectional survey

Lelisa Fekadu[1]*, Eyerusalem Negussie[2], Abdulrahman Jbaily[3], Mieraf Taddesse Tolla[3], Kjell Arne Johansson[1]

## *S1. Consumption construction*

In this study, income (for HIV) and consumption as proxy for income (for tuberculosis) were used as household welfare measure. The consumption aggregates for tuberculosis (TB) was constructed using the overall value of the food (purchased food, home produced food) items, non-food items, and housing information collected at different recall periods (ranging from 1 to 12 months depending on how frequently the item purchased). Then, all reported expenditures on food items, non-food items, and housing were converted to a month period, then added up and multiplied by 12 to create annual consumption for each household.

The income (for HIV) and consumption (for TB) measures were also adjusted for family size and demographic composition to reflect economy of scale. We constructed per adult equivalence through dividing household income/consumption expenditure by an adult equivalent scale. Adult equivalent values (AE) were calculated using $AE = (A + \alpha K)^{\theta}$ for HIV, where $A$ stands for number of adults, $K$ is the number of children in the household, $\alpha$ is the cost of a child relative to an adult (0.33) and $\theta$ is the degree of economies of scale (0.9) (1). Whereas for TB, due to the unavailability of child data, we calculated using $AE = hsize^{\beta}$ , where "$hsize$" is the actual household size and β was set to be 0.56 (2). Using per adult equivalence income (HIV) and consumption (TB) values, we grouped all households into five income quintiles of equal size.

***S2. Incidence and intensity of catastrophic health expenditure (CHE)***

***S2.1. Measuring incidence of CHE (Headcount)***

Catastrophic health expenditure (headcount, H) occurs when household OOP health spending exceeds a predefined threshold (10%) of household income/consumption. Furthermore, we conducted analysis at 20% threshold of both OOP and total cost (new definition of catastrophic cost recommended for TB), and at 40% of non-food expenditure (i.e. net of basic subsistence expenditure).

The CHE headcount is calculated using the equation

$$H = \frac{1}{N}\sum_{i=1}^{N} E_i \,,$$

Where, $E_i = 1$ if $\frac{T_i}{X_i}$ or $\frac{T_i}{(X_i - FE_i)} > z$ and 0 otherwise.

Here, N equals the total sample size, $T_i$ is the OOP health spending of household $i$, $X_i$ is the total expenditure of household $i$, $FE_i$ is the food expenditure of household $i$ and $z$ is the specified threshold.

***S2.2. Measuring intensity of CHE***

The intensity is measured using overshoot (O) and mean positive overshoot (MPO).

***S2.2.1. Overshoot***

The overshoot measures the extent of average expenditure exceeding the given threshold in the entire sample. The overshoot is calculated using the equation

$$O = \frac{1}{N}\sum_{i=1}^{N} (E_i \left(\frac{Ti}{Xi}\right) - z)$$

***S2.2.2. Mean positive overshoot***



Assebe LF, *et al. BMJ Open* 2020; 10:e036892. doi: 10.1136/bmjopen-2020-036892

The mean positive overshoot measures the extent of an average expenditure exceeding the threshold among households experiencing CHE, and is calculated by:

$$MPO = \frac{O}{H}$$

**Table A1.** Mean (median) patient costs per TB case across three types of TB (Ethiopia) expressed in $.

| Income quintiles | Type of TB | | | | | |
|---|---|---|---|---|---|---|
| | Pulmonary[¥] | | Extra-pulmonary TB[¥] | | Drug resistant-TB | |
| | Mean (SD) | Median (IQR) | Mean (SD) | Median (IQR) | Mean (SD) | Median (IQR) |
| **Poorest** | 46 (43) | 32 (13-62) | 60 (45) | 46 (32-75) | 295 (290) | 139 (65-607) |
| **Poor** | 67 (44) | 56 (34-85) | 107 (90) | 83 (49-136) | 159 (85) | 153 (68-199) |
| **Middle** | 102 (80) | 75 (48-137) | 115 (74) | 94 (72-131) | 205 (175) | 156 (86-250) |
| **Rich** | 133 (99) | 102 (65-176) | 155 (86) | 133 (79-212) | 534 (671) | 291 (131-669) |
| **Richest** | 175 (165) | 121 (78-216) | 262 (226) | 212 (112-294) | 971 (1356) | 610 (201-821) |
| **Total*** | 104 (107) | 73 (41-135) | 140 (138) | 96 (58-180) | 446 (732) | 191 (107-607) |

* kruskal wallis test p-value <0.001

¥ CHE at 10% threshold ranged from 33% to 57% for pulmonary and extra-pulmonary TB, respectively.

**Table A2.** Incidence and intensity of CHE for HIV and TB across different income quintiles and threshold, 2019, Ethiopia

| Disease | Measure Of CHE | Threshold | Average | Income Quantile | | | | |
|---|---|---|---|---|---|---|---|---|
| | | | | Q1 | Q2 | Q3 | Q4 | Q5 |
| **HIV** | Out-of-pocket HIV spending: as a share of annual income | | | | | | | |
| | Head count (CHE incidence) % | 10% | 20 | 43 | 27 | 15 | 10 | 4 |
| | Overshoot % | 10% | 7.6 | 26.2 | 7.5 | 2.7 | 0.9 | 0.9 |
| | Mean positive overshoot % | 10% | 39.1 | 61.8 | 28.2 | 18.3 | 9.9 | 17.8 |
| | Out-of-pocket HIV spending: as a share of annual income | | | | | | | |
| | Head count (CHE incidence) % | 20% | 11 | 30 | 14 | 6 | 3 | 1 |
| | Overshoot % | 20% | 6.2 | 22.5 | 5.6 | 1.7 | 0.5 | 0.6 |
| | Mean positive overshoot % | 20% | 58.0 | 74.9 | 38.6 | 28.2 | 18.2 | 122.0 |
| | Total HIV spending: as a share of annual income | | | | | | | |
| | Head count (CHE incidence) % | 20% | 15 | 38 | 18 | 11 | 6 | 3 |
| | Overshoot % | 20% | 7.2 | 25.4 | 6.4 | 2.5 | 0.8 | 0.8 |
| | Mean positive overshoot % | 20% | 48.6 | 67.8 | 36.2 | 23.6 | 15.0 | 27.7 |
| | Out-of-pocket HIV spending: as a share of total non-food expenditure | | | | | | | |
| | Head count (CHE incidence) % | 40% | 11 | 31 | 15 | 7 | 3 | 1 |
| | Overshoot % | 40% | 13.1 | 47.4 | 11.8 | 3.6 | 1.0 | 1.2 |
| | Mean positive overshoot % | 40% | 116.3 | 153.0 | 79.5 | 55.4 | 39.7 | 128 |
| **TB** | Out-of-pocket TB spending: as a share of annual income | | | | | | | |
| | Head count (CHE incidence) % | 10% | 40 | 58 | 48 | 38 | 35 | 20 |
| | Overshoot % | 10% | 6.3 | 15.8 | 7.5 | 4.1 | 2.7 | 1.9 |
| | Mean positive overshoot % | 10% | 15.3 | 26.6 | 15.7 | 10.7 | 7.6 | 9.2 |

| | | | | | | | |
|---|---|---|---|---|---|---|---|
| | Out-of-pocket TB spending: as a share of annual income | | | | | | |
| | Head count (CHE incidence) % | 20% | 17 | 38 | 23 | 10 | 8 | 6 |
| | Overshoot % | 20% | 3.7 | 10.7 | 4.1 | 1.9 | 0.7 | 0.8 |
| | Mean positive overshoot % | 20% | 21.3 | 27.8 | 17.7 | 18.7 | 8.8 | 15.2 |
| | Total TB spending: as a share of annual income | | | | | | | |
| | Head count (CHE incidence) % | 20% | 48 | 60 | 53 | 48 | 46 | 36 |
| | Overshoot % | 20% | 10.0 | 18.6 | 11.0 | 8.0 | 6.7 | 4.4 |
| | Mean positive overshoot % | 20% | 20.0 | 31.1 | 20.9 | 16.7 | 14.7 | 12.3 |
| | Out-of-pocket HIV spending: as a share of total non-food expenditure | | | | | | | |
| | Head count (CHE incidence) % | 40% | 37 | 50 | 43 | 39 | 33 | 19 |
| | Overshoot % | 40% | 29.2 | 73.0 | 27.5 | 18.6 | 14.9 | 11.6 |
| | Mean positive overshoot % | 40% | 78.7 | 146 | 63.6 | 47.7 | 44.7 | 59.5 |
| | Where, Q1 = Poorest; Q2 = Poorer; Q3 = Middle; Q4 = Richer; Q5 = Richest. | | | | | | | |



Assebe LF, *et al. BMJ Open* 2020; 10:e036892. doi: 10.1136/bmjopen-2020-036892

## References


1. Deaton A, Zaidi S. Guidelines for constructing consumption aggregates for welfare analysis. 2002.
2. Xu K, Evans DB, Kawabata K, Zeramdini R, Klavus J, Murray CJ. Household catastrophic health expenditure: a multicountry analysis. Lancet (London, England). 2003;362(9378):111-7.
