# Supplementary material for: Financial burden of HIV and TB among patients in Ethiopia: a cross-sectional survey
Source: BMJ Open. 2020 Jun 1;10(6):e036892. doi: 10.1136/bmjopen-2020-036892 (PMC7265036; doi:10.1136/bmjopen-2020-036892)
Supplement: Author's manuscript [file bmjopen-2020-036892.draft_revisions.pdf]

BMJ Open is committed to open peer review. As part of this commitment we make the peer review history of every article we publish publicly available.

When an article is published we post the peer reviewers' comments and the authors' responses online. We also post the versions of the paper that were used during peer review. These are the versions that the peer review comments apply to.

The versions of the paper that follow are the versions that were submitted during the peer review process. They are not the versions of record or the final published versions. They should not be cited or distributed as the published version of this manuscript.

BMJ Open is an open access journal and the full, final, typeset and author-corrected version of record of the manuscript is available on our site with no access controls, subscription charges or pay-per-view fees (http://bmjopen.bmj.com).

If you have any questions on BMJ Open's open peer review process please email info.bmjopen@bmj.com

BMJ Open

The financial burden of HIV and TB among patients in Ethiopia: a cross-sectional survey

Journal:	*BMJ Open*
Manuscript ID	bmjopen-2020-036892
Article Type:	Original research
Date Submitted by the Author:	09-Jan-2020

Complete List of Authors:	Assebe, Lelisa; University of Bergen Faculty of Medicine and Dentistry, Global Public Health and Primary Care Medicine; Ethiopia Ministry of Health, Disease Prevention and control<br>Negussie, Eyerusalem; Ethiopia Ministry of Health, Disease prevention and control program<br>Jbaily, Abdulrahman; Harvard University T H Chan School of Public Health, Global Health and Population<br>Tolla, Mieraf Taddesse; Harvard University T H Chan School of Public Health,Global Health and Population<br>Johansson, Kjell Arne; University of Bergen Faculty of Medicine and Dentistry, Department of Global Public Health and Primary care

Keywords:	Tuberculosis < INFECTIOUS DISEASES, HIV & AIDS < INFECTIOUS DISEASES, HEALTH ECONOMICS

SCHOLARONE™ Manuscripts

I, the Submitting Author has the right to grant and does grant on behalf of all authors of the Work (as defined in the below author licence), an exclusive licence and/or a non-exclusive licence for contributions from authors who are: i) UK Crown employees; ii) where BMJ has agreed a CC-BY licence shall apply, and/or iii) in accordance with the terms applicable for US Federal Government officers or employees acting as part of their official duties; on a worldwide, perpetual, irrevocable, royalty-free basis to BMJ Publishing Group Ltd ("BMJ") its licensees and where the relevant Journal is co-owned by BMJ to the co-owners of the Journal, to publish the Work in this journal and any other BMJ products and to exploit all rights, as set out in our licence.

The Submitting Author accepts and understands that any supply made under these terms is made by BMJ to the Submitting Author unless you are acting as an employee on behalf of your employer or a postgraduate student of an affiliated institution which is paying any applicable article publishing charge ("APC") for Open Access articles. Where the Submitting Author wishes to make the Work available on an Open Access basis (and intends to pay the relevant APC), the terms of reuse of such Open Access shall be governed by a Creative Commons licence – details of these licences and which Creative Commons licence will apply to this Work are set out in our licence referred to above.

Other than as permitted in any relevant BMJ Author's Self Archiving Policies, I confirm this Work has not been accepted for publication elsewhere, is not being considered for publication elsewhere and does not duplicate material already published. I confirm all authors consent to publication of this Work and authorise the granting of this licence.

For peer review only

Title

The financial burden of HIV and TB among patients in Ethiopia: a cross-sectional survey

Lelisa Fekadu Assebe[1]*, Eyerusalem Negussie[2], Abdulrahman Jbaily[3], Mieraf Taddesse Tolla[3], Kjell Arne Johansson[1]

1. Department of Global Public Health and Primary Care Medicine, University of Bergen, Bergen, Norway

2. Disease prevention and control program, Ministry of Health, Addis Ababa, Ethiopia

3. Department of Global Health and Population, Harvard T.H. Chan School of Public Health, Harvard University, Boston, MA, USA

*Correspondence: lelfekadu1@gmail.com

For peer review only

ABSTRACT

Objectives: Human Immunodeficiency Virus (HIV) and Tuberculosis (TB) are major global health threats and can result in household financial hardships. Here, we aim to estimate the household economic burden and the incidence of catastrophic health expenditures (CHE) incurred by HIV and TB care across income quintiles in Ethiopia.

Design: A cross-sectional survey.

Setting: 27 health facilities of Afar and Oromia regions for TB, and nationwide household survey for HIV.

Participants: A total of 1,006 and 787 individuals seeking HIV and TB care were enrolled, respectively.

Outcome measures: The economic burden (i.e. direct and indirect cost) of HIV and TB care was estimated. In addition, the CHE incidence and intensity was determined using direct costs exceeding 10% of the household income threshold.

Results: The mean (SD) age of HIV and TB patient was 40 (10), and 30 (14) years, respectively. The mean (SD) patient cost of HIV was \$91 (\$199) per year and \$115 (\$118) per TB episode. Out of the total cost, the direct cost of HIV and TB constituted 69% and 46%, respectively. The mean (SD) indirect cost was \$28 (\$77) per year for HIV and \$63 (\$83) per TB episode. The incidence of CHE for HIV was 22%; ranges from 46% in the poorest to 6% in the richest income-quintile (P <0.001). Similarly, for TB, the CHE incidence was 40% and ranged between 58% and 20% among the poorest and richest income-quintiles respectively (P <0.001). This figure was higher for drug resistant TB (62%).

Conclusions: HIV and TB are causes of substantial economic burden and CHE, inequitably, affecting those in the poorest income quintile. Broadening the health policies to encompass interventions that reduce the high cost of HIV and TB care, particularly for the poor, is urgently needed.

For peer review only - http://bmjopen.bmj.com/site/about/guidelines.xhtml

Strengths and limitations of this study

- We estimated the cost, incidence, and determinants of CHE among individuals seeking HIV and TB care in Ethiopia.
- The cost of care was further disaggregated by inpatient/outpatient status, and income quintile.
- Our findings will inform policies related to patient centred care and financial risk protection of both diseases, which is central in achieving universal health coverage.
- The cost measurements relied on patient's ability to remember, which increases risk of recall bias.
- The sampling for HIV primarily consisted of urban population, and the TB study is limited to specific regions of the country that limits the representatives of our finding to all PLHIV and TB patients in Ethiopia.

view only

For peer review only - http://bmjopen.bmj.com/site/about/guidelines.xhtml

BACKGROUND

HIV and TB are major global health threats that cause a large financial burden on vulnerable populations. Global efforts, in the past two decades, have improved access to lifesaving HIV and TB interventions [1-3]. More than 72 million lives have been saved between 2000 – 2018 [4, 5]. Nevertheless, high disease burden, inequality in utilisation of healthcare and service quality issues still exists [1-3].

Globally, in 2018 alone, an estimated 1.7 million people acquired HIV infection, and 10 million new TB cases occurred. In the same period, more than 2 million people died from HIV and TB [4, 6]. In Ethiopia, 2018, the adult (aged between15-49 years) HIV prevalence was estimated to be 1% (confidence interval (CI): 0.7%-1.4%) and the TB incidence rate was 151/100,000 populations [4, 6]. In order to end HIV and TB, a comprehensive approach should include medical and non-medical interventions such as socio-economic support and poverty alleviation. This is fundamental to ascertain the status of universal health coverage (UHC) [7, 8].

Although many countries, including Ethiopia, offer ''free'' and ''decentralized'' HIV and TB services, the implemented policies do not adequately provide realistic financial risk protection. The health budget in Ethiopia is low ($33.2 per capita) [9]. Out-of- pocket payment (OOP) is a major source of health financing that accounts 31% of the total health finances [9, 10]. Hence, patient and their families, often face both direct and indirect costs, which create a substantial financial burden on households [11, 12]. A systematic review (where 32% of the studies were from Africa) showed that individuals spend on average $ 847 (range: $ 55 - $ 8,198) per TB episode [11]. Similarly, for HIV, spending ranges from $ 95 - $ 2,672 in sub-Saharan Africa [12]. Such high costs are related with catastrophic health expenditure (CHE), which occurs when the OOP exceeds 10% of annual income [13, 14] or 40% of household non-food expenditure [15]. Furthermore, high levels of patient cost may hinder access to care, and lead to poor treatment outcome and prolonged period with infection [12, 13, 16-18].

For peer review only - http://bmjopen.bmj.com/site/about/guidelines.xhtml

A lot of factors could contribute to CHE; exemptions of HIV and TB services often applies to limited aspects of the basic care package (e.g. CD_4, viral load, AFB microscopy/Gene X-pert tests), treatment (e.g. ART, anti TB medicines) and not, for instance for pre-diagnosis tests (i.e. X-ray, haematology), adverse event monitoring, non- HIV and TB medicines, hospitalization, transport, food, lodging, and lost working days, which patients pay for [16, 19-21]. In addition, unavailability of diagnostic services in public health facilities pushes patients to seek care from expensive private providers [19]. Furthermore, low health insurance coverage (around 24% of households covered in Ethiopia as of 2019), longer treatment duration, and repeated follow up visits were important contributing financial risk factors [22, 23]. Hence, OOP expenses for HIV and TB care may crowd out consumption of basic needs and leave vulnerable households in debt/impoverishment [12, 24, 25]. It is also imperative that HIV and TB programs, as part of the sustainable development goal (SDG), needs to monitor household protection from CHE (i.e. financial risk protection (FRP)) [26]. Similarly, the distribution of financial risk across income groups is important from an equity perspective, as OOP health spending imposes a greater burden on the poor [27].

In Ethiopia, few studies have been evaluating the level of patient cost due to HIV and TB care seeking. The costs related to severe forms of the diseases and assessments across income or consumption groups were lacking from the studies reviewed [19, 20, 28]. Furthermore, none studied predictors of CHE [19, 20, 28]. Because HIV and TB are chronic diseases and intimately linked, it is reasonable to look at them jointly. This paper aims to estimate the economic burden and incidence of CHE incurred by standard HIV and TB care across income or consumption quintile among Ethiopian households. Moreover, we will assess factors associated with CHE for HIV and TB.

For peer review only - http://bmjopen.bmj.com/site/about/guidelines.xhtml

METHODS

Study setting and population

In this study, a nationwide household survey for HIV [9] and a cross sectional health facility based survey for TB, were used to estimate direct and indirect costs, and CHE.

Data for HIV were collected from mid-September to mid-October 2014 and represent the total population of people living with HIV (PLHIV) in the country. The total estimated number of PLHIV were 722,747 in Ethiopia. However, for TB, data were collected in three zones (i.e. zone 3 of Afar region, and Jimma and Adama special zones of Oromia region) from December 2018 to September 2019. In total, around 4 million people live in these three zones and mirrors the geographic and socio-economic heterogeneity of Ethiopia. The zones account for 10% and 13% of the TB cases in Oromia and Afar regions (and 6% of the national prevalence), respectively.

Sample size and sampling technique

For HIV, the estimated sample size was 4,200. The response rate ranges from 92% to 100% across regions. A two-stage stratified cluster-sampling method was employed. In stage one, a sample of 105 HIV associations from a total of 588 were randomly selected and allocated to each region using probability proportional to the size. In stage two, 40 HIV members, in total 4,171, were randomly selected and interviewed. Among the study participants (i.e. 4,171), 1,006 had HIV related illness and were considered in our analysis.

For TB, the sample size was calculated using two-population proportion formula with 80% power, 5% type I error, 95% confidence level, and using 39% [18] of households incur CHE among the richest income quartile; and to detect 15% point difference revealed 186 samples for each quartile. The final sample size with 10% non-response rate was 818 (of which 7% were DR-TB). The three zones for the TB study were purposely selected for administrative convenience. Using systematic random sampling, 27 public health facilities was selected from the three zones. The total sample size was distributed proportional to the TB case load [29].

For peer review only - http://bmjopen.bmj.com/site/about/guidelines.xhtml

Patient and public involvement

The research question of this study is in line with the Ethiopian tuberculosis research plan developed through multiple consultative process with engagement of wider stakeholders, including patient representatives. We intend to disseminate the research findings through the national TB research conferences with wider involvement of researchers, policy makers, partners and affected communities.

Data collection tools and quality assurance

A standardized questionnaire, adapted from United States Agency International Development (USAID) for HIV and the World Health Organization (WHO) patient costing tool for TB, was used for data collection [30, 31]. The questionnaire captures sociodemographic variables, direct cost, indirect cost, productivity loss, assets, income, consumptions and coping related information. We complemented clinical information for TB through review of medical records. The questionnaire was translated into local languages, pre-tested and modified accordingly. Trained data collectors under close supervision undertook the face-to-face interview. TB patients with a minimum of one month on treatment were interviewed consecutively and reported on expenditures retrospectively. Whereas, for HIV, the expenditure in the past four weeks for outpatient and six months for inpatient care were gathered.

Patient cost

The costs of seeking standard HIV and TB care were estimated from patient perspective. The expenditure related to routine care, HIV related opportunistic infection, and co-morbidities were considered for HIV. For TB episode, the expenditure in the TB patient pathway from onset of symptoms, diagnosis and treatment completion were included.

Direct costs were household expenditures for medical (i.e. registration/consultation fees, laboratory tests, X-ray, medicines, hospital admission) and non-medical services (i.e. special food/nutrition, transportation, and guardian cost) net of reimbursement. Indirect costs

For peer review only - http://bmjopen.bmj.com/site/about/guidelines.xhtml

constitutes lost income following the disease episode. In order to estimate the indirect cost, patients were asked to estimate the time lost due to receiving and waiting for care, hospitalization, transportation, lost working days and guardian time (TB). Then the total time lost was multiplied by an hourly wage rate, which was derived from monthly income/consumption by assuming 22 working days a month and 8 hours a day. For children less than 15 years of age, non-medical direct and indirect TB costs was computed for the guardian. Total patient cost is the sum of all direct and indirect costs.

In order to annualize the cost, an average of four and TB episode outpatient visits was considered for HIV and TB, respectively. The TB patient cost was extrapolated for the whole duration of TB episode based on an individual data reported retrospectively. All costs were gathered in local currency (Ethiopian Birr) and converted to US dollar with the 2019 exchange rate of $ 1= 29.1468 Ethiopian Birr [32]. For HIV, the cost was first converted to a reference period (2019) using Ethiopia's consumer price index [33]. Due to unavailability of data, we used household income (for HIV) and consumption aggregates (for TB) as a proxy for the household welfare level and scaled to per adult equivalence in order to reflect family size and composition (Supplementary 1, S1). In addition, participants were grouped into five-income/consumption quintiles to reflect the socio-economic strata (supplementary 1, S1).

An incidence of CHE occurs when direct costs (i.e. OOP) exceed a 10% threshold of annual household income/consumption [13, 14, 24]. In addition to the 10% threshold, we conducted further analysis at 20% threshold of both OOP and total cost, and at 40% of non-food expenditure [15, 18, 30, 34]. Furthermore, the distribution of financial burden (measured as ratio of direct/total costs to total household expenditure) across income quintile was reported using headcount, overshoot and mean positive overshoot [35] (supplementary 1, S2).

For peer review only - http://bmjopen.bmj.com/site/about/guidelines.xhtml

Data analysis

Data was analysed using STATA version 16 software. The data was summarized using proportions, mean with standard deviation (SD) or median with interquartile range (IQR). The cost was disaggregated by outpatient and inpatient care. A concentration index was used to assess health outcome measure inequality across income quintiles [35, 36]. Multivariate logistic regression was conducted to identify independent determinants of CHE by including significant variables in univariate analysis. A stepwise regression approach was employed to develop the final model and adjusted odds ratios (aOR) with 95% CI was reported. P-value less than 0.05 declares statistical significance for each tests. Multicollinearity was ruled out (variance inflation factor <5). Goodness of fit was checked by Hosmer–Lemeshow test [37].

Ethical consideration

The study was approved by medical and health research ethics in Norway (2018/1647/REK) and Ethiopian public health institute (EPHI-IRB-121-2018). Informed written consent was obtained from TB participants. Oromia and Afar regional health bureau, and respective zonal health offices provided permission to undertake the TB study.

For peer review only

For peer review only - http://bmjopen.bmj.com/site/about/guidelines.xhtml

RESULTS

Out of the total 1,006 HIV and 787 TB participants, 75% of HIV and 50% of TB were females (Table 1). More than two thirds of the study participants were in the age group between 25-44 years for HIV and 18-34 years for TB. The median family size was 4 (IQR, HIV: 3–5, IQR, TB: 3-6). Seven percent of TB patients were co-infected with HIV. Almost all (99%) of HIV and 91% of TB/HIV co-infected were receiving ART. Nearly 22% and 6% of HIV and TB participants had one episode of hospitalization, respectively. The mean hospital stay was 11 days for HIV and 10 days for TB. The mean (SD) household annual income/consumption per adult equivalence was $1,188 ($1,288) for HIV and $545 ($462) for TB. The mean (SD) time interval from first health care visit to TB diagnosis (i.e. health system delay) was 14 (38) days and 85% of TB were diagnosed in public health facilities.

Table 1: Socio-demographic and clinical characteristics of HIV and TB study participants (Ethiopia).

**Background characteristics**		**HIV (N=1006)**	**TB (N=787)**
		**N (%)**	**N (%)**
Gender	Female	756 (75)	396 (50)
Age in years (mean, SD)		40 (10)	30 (14)
Age group	<18	-	110 (14)
	18-24	14 (1)	190 (24)
	25-34	268 (27)	229 (29)
	35-44	435 (43)	117 (15)
	45-54	202 (20)	86 (11)
	55-64	58 (6)	32 (4)
	65+	29 (3)	23 (3)
Marital status	Single	38 (4)	309 (39)
	Married/Living together	493 (49)	409 (52)
	Widowed	277 (27)	24 (3)
	Divorced	137 (14)	33 (4)
	Separated	61 (6)	11 (2)
Place of residence	Urban	930 (92)	394 (50)
	Rural	76 (8)	393 (50)
Highest level of education	Illiterate	290 (29)	257 (33)
	Elementary	457 (45)	334 (42)
	Secondary and Higher	259 (26)	195 (25)
Family size	≤ 4	640 (64)	443 (56)
	>4	366 (36)	344 (44)
Annual household	Lowest	200 (20)	159 (20)

For peer review only - http://bmjopen.bmj.com/site/about/guidelines.xhtml

income/consumption	Second	470 (21)	298 (20)
	Middle	797 (18)	443 (20)
	Fourth	1,342 (20)	631 (20)
	Highest	3,084 (21)	1,198 (20)
ART status	On ART	996 (99)	52 (91)
	Not on ART	10 (1)	5 (9)
Past history of illness*	Yes	1,006 (24)	61 (8)
	No	3,165 (76)	726 (92)
Type of visit	Outpatient	790 (79)	739 (94)
	Inpatient	216 (22)	47 (6)
Number of visits per year/TB episode	Outpatient	4,428 (4 visit/patient)	48,720 (70 visit/patient)**
	Inpatient	249 (1 visit/patient)	2,409 (73 visit/patient)**
Type of TB	Pulmonary TB	-	507 (65)
	Extra-pulmonary TB	-	222 (28)
	Drug resistant TB	-	57 (7)

*HIV related comorbidities (HIV), and history of TB (TB)

** The number of visits per patient reaches 125 for outpatient and 135 for inpatient drug resistant TB cases.

Patient cost of HIV and TB care

The total mean (SD) patient cost for HIV care was \$91 (\$199) per year and \$115 (\$118) for the entire duration of a TB episode (Table 2). The mean (SD) direct cost was \$63 (\$168) for HIV and \$53 (\$59) for TB, which constitutes 68% and 46% of the total cost, respectively. Medical costs contributed to 69% and 38% of the direct costs for HIV and TB, respectively. Diagnostics and medicine account for 39% of the total HIV cost. The mean (SD) indirect cost was \$28 (\$77) for HIV per year, and \$63 (\$83) per TB episode. The productivity loss related to TB follow-up visits accounts for 36% of the total cost.

The mean (SD) total cost was \$73 (\$192) for annual outpatient HIV care visit and \$110 (\$114) for the whole duration of outpatient TB care (Table 2). Similarly, the mean (SD) total cost for each hospitalization period was \$112 (\$162) for HIV and \$105 (\$78) for TB. For patients having both outpatient and inpatient visits, the mean cost reaches \$156 (\$208) ($p<0.001$) for HIV and \$ 217 (\$157) ($p<0.001$) for TB.

For TB, the patient costs incurred from the onset of illness to initiation of TB treatment were equal to the cost from treatment initiation up to completion (paired t-test>0.05). The mean (SD)

For peer review only - http://bmjopen.bmj.com/site/about/guidelines.xhtml

total cost for those with TB/HIV co-infection reached $188 ($33). Similarly, the total cost of care significantly varies by type of TB, was $104 ($107) for pulmonary TB, $140 ($138) for extra-pulmonary TB and $446 ($732) for DR-TB (Kruskal Wallis test 41.1, P <0.001) (supplementary 1, Table A2).

Coping costs

HIV and TB care results in adverse financial consequences for households. Nearly 24% of HIV and 68% of TB study participants have adopted coping mechanisms. Nine percent of HIV and 4% of TB patients have borrowed money; while, 2% of HIV and 19% of TB patients sold their household assets. Furthermore, 12% of HIV patient relied on family assistance and 16% of TB patient used their savings to cope with the costs. Only 2% of HIV and 6% of TB participants are covered by health insurance.

Table 2: Distribution of household direct, indirect and total cost of HIV and TB care across main cost category (Ethiopia).

Cost category		HIV (N=1,006)			TB (N=729)		
		Outpatient (Per year)	Inpatient (Per singl visit)	Total	Outpatient (Per TB episode)	Inpatient (Per singl visit)	Total
**I. Direct medical cost**							
Consultation fee	Mean (SD)	7 (17)	2 (6)	7 (17)	2 (5)	11 (16)	3 (7)
	Median (IQR)	0 (0-7)	0 (0-1)	0 (0-6)	1 (0-2)	7 (1-14)	1 (0-2)
Investigation cost	Mean (SD)	17 (52)	10 (22)	17 (54)	8 (13)	14 (16)	9 (15)
	Median (IQR)	0 (0-13)	0 (0-11)	1 (0-14)	4 (0-12)	7 (3-18)	5 (0-13)
Drug cost[1]	Mean (SD)	14 (46)	26 (74)	18 (56)	7 (14)	16 (21)	8 (16)
	Median (IQR)	0 (0-12)	5 (0-22)	1 (0-15)	1 (0-10)	8 (3-20)	2 (0-10)
**Subtotal**	Mean (SD)	37 (103)	39 (90)	43 (114)	17 (24)	40 (37)	20 (30)
	Median (IQR)	5 (0-40)	10 (0-37)	7 (0-44)	10 (1-22)	30 (16-45)	11 (1-25)
**II. Direct non-medical cost**							
Transportation fee	Mean (SD)	12 (65)	23 (39)	15 (61)	8 (10)	7 (7)	8 (10)
	Median (IQR)	3 (0-9)	7 (0-23)	3 (0-11)	5 (2-11)	7 (3-9)	5 (2-11)
Food/accommodation	Mean (SD)	4 (59)	11 (35)	6 (56)	23 (29)	28 (34)	25 (31)
	Median (IQR)	0	0 (0-6)	0	15 (7-27)	16 (7-38)	16 (7-29)
**Subtotal**	Mean (SD)	16 (93)	33 (57)	20 (89)	31 (34)	35 (36)	33 (37)
	Median (IQR)	3 (0-9)	11 (0-45)	3 (0-11)	21 (11-38)	20 (9-44)	21 (12-40)
**Total direct cost**	Mean (SD)	54 (166)	70 (132)	63 (168)	48 (50)	75 (68)	53 (59)
	Median (IQR)	13 (1-53)	25 (5-87)	18 (2-67)	35 (17-61)	54 (26-94)	36 (18-64)
**III. Indirect cost**							
Foregone income before treatment[2]	Mean (SD)	0	0	0	11 (28)	0	11 (26)
	Median (IQR)	0	0	0	0 (0-5)	0	0 (0-3)

For peer review only

Foregone income during treatment	Mean (SD)	12 (51)	25 (48)	18 (59)	11 (26)	12 (31)	10 (26)
	Median (IQR)	0	10 (4-23)	0 (0-9)	0 (0-4)	0	0 (0-3)
Time loss related cost	Mean (SD)	7 (22)	16 (61)	9 (35)	42 (59)	18 (24)	42 (58)
	Median (IQR)	3 (1-5)	3 (1-8)	3 (1-6)	27 (14-49)	8 (5-22)	27 (14-49)
**Subtotal**	Mean (SD)	19 (63)	41 (87)	28 (77)	62 (84)	30 (37)	63 (83)
	Median (IQR)	3 (2-9)	15 (6-37)	4 (2-20)	36 (17-77)	9 (5-39)	36 (17-78)
**IV. Total cost**	Mean (SD)	73 (192)	112 (162)	91 (199)	110 (114)	105 (78)	115 (118)
	Median (IQR)	24 (5-74)	60 (25-127)	31 (8-93)	79 (46-140)	87(39-158)	81 (47-150)

[1]Drug other than anti-retroviral and anti TB drugs
[2]Not captured in HIV survey

As shown in figure 1, the spikes in the pen's parade graph showed that the health care costs of HIV and TB cause a substantial decrease in annual income/consumption for many of the households. The consumption drop is more pronounced for TB as compared to the income drop for HIV (Figure 1).

[Figure 1 here]

As shown in Table 3, there are inequalities in OOP costs among HIV and TB participants. The total cost rises steadily across the income quintiles and is concentrated among the richer quintiles (i.e. the richer the quintile, the higher the cost). From the lowest to the highest income/consumption quintile, the mean (median) total cost of HIV increases from \$61 (\$23) to \$156 (\$69), with significant difference among income quintiles (Kruskal Wallis test 44.7, $P < 0.001$), and for TB the cost increases from \$50 (\$36) to \$202 (\$148) (Kruskal Wallis test 206.5, $P < 0.001$). In general, the median cost (both direct and indirect) seems to be a bit higher for TB patients as compared to those with HIV.

Table 3. Mean (median) HIV and TB patient costs per year across income quintiles (Ethiopia).

**Disease Category**	**Income quintiles**	**Cost type**					
		**Direct**		**Indirect**		**Total cost**	
		Mean (SD)	Median (IQR)	Mean (SD)	Median (IQR)	Mean (SD)	Median (IQR)
**HIV**	Poorest	53 (108)	17 (2-56)	9 (17)	2 (1-8)	61 (113)	23 (4-71)
	Poor	64 (199)	15 (0-61)	12 (22)	3 (1-10)	75 (202)	25 (4-76)
	Middle	58 (138)	22 (3-57)	18 (35)	3 (2-19)	76 (159)	37 (8-76)
	Rich	54 (104)	18 (3-66)	29 (60)	5 (3-24)	83 (130)	31 (8-95)
	Richest	85 (240)	22 (3-93)	70 (145)	16 (5-68)	156 (305)	69 (14-154)
**P-value***		0.358		<0.001		<0.001	

For peer review only - http://bmjopen.bmj.com/site/about/guidelines.xhtml

**TB**	Poorest	31 (35)	23 (7-41)	19 (20)	11 (6-24)	50 (44)	36 (20-66)
	Poor	44 (48)	27 (15-54)	36 (32)	27 (13-45)	79 (65)	60 (38-96)
	Middle	49 (53)	36 (17-61)	57 (46)	40 (22-82)	106 (79)	82 (57-137)
	Rich	61 (48)	49 (28-84)	79 (63)	58 (27-117)	140 (96)	118 (68-181)
	Richest	78 (86)	54 (34-99)	124 (145)	70 (40-163)	202 (189)	148 (88-260)
**P-value***		< 0.001		< 0.001		< 0.001	

*kruskal wallis test

Incidence and intensity of CHE

At the 10% threshold, the overall CHE incidence of HIV was 22% (219 households); with 46% of the poorest and 6% of the richest household experiencing CHE (Chi-square for trend -10.77, P <0.001) (Figure 2a, Supplementary 1-Table A1). The incidence is 36% for individuals with inpatient HIV care.

The corresponding level of TB was 40% (291 households); with 58% and 20% of the poorest and richest income-quintile experienced CHE, respectively (Chi-square for trend -6.79, P <0.001) (Table 4). The incidence was much higher for those with TB/HIV co-infection (48%), DR-TB (62%) and was almost universal (94%) for TB patient that had hospitalization episode. At the 20% threshold of total expenses, 48% (353 households) of TB households experienced catastrophic total costs (Fig 2b, Supplementary 1-Table A1).

In our study, for example, the mean overshoot of TB related CHE was 6.3% (range: 1.9%-15.3%). On average, households spent 6.3% beyond the 10% threshold for TB care. However, the average positive overshoot among households that experienced CHE was 15.8% (range: 9.2% - 26.6%). Thus, on average, households that experienced CHE spent 25.8% (10% threshold + mean positive overshoot) of their total annual consumption for TB care (Figure 2, Supplementary 1-Table A1).

[Figure 2 here]

For peer review only - http://bmjopen.bmj.com/site/about/guidelines.xhtml

Inequality in financial risk

In addition, as shown in figure 3, inequality in financial risk across income/consumption groups exists. The concentration curves for HIV and TB care costs lies below the 45° line of equality, which shows a greater concentration of the costs among the rich. However, the financial burden is higher among the poor – the concentration curves for HIV and TB care expenditure in relation to income/consumption lies above the 45° line of equality.

[Figure 3 here]

Determinants of CHE

In the multivariate analysis (Table 4), three variables were independently associated with HIV related CHE: hospitalized patients (aOR: 3.3, 95% CI: 2.3-4.9), being poorest (aOR: 15.4, 95% CI: 8.0-29.5) and poor (aOR: 8.2, 95% CI: 4.2-15.8) were associated with catastrophic HIV care expenditures. Moreover, every additional visit for HIV care increases the odds of CHE by 7% (aOR: 1.07, 95% CI: 1.01-1.1).

Seven variables were significantly associated with TB related CHE (Table 4): private facility diagnosis (aOR: 2.6, 95%CI: 1.52-4.33); extra-pulmonary TB (aOR: 2.6, 95%CI: 1.77-3.95); hospitalized patients (aOR: 30.6, 95% CI: 4.77-199.83); being poorest (aOR: 14.6, 95% CI: 7.49-28.26); and TB/HIV co-infection (aOR: 3.2, 95% CI: 1.63-6.15) were very likely to have TB related CHE as compared to their counterparts after adjusting for other variables. Every additional visit for TB diagnosis increases the odds of experiencing CHE by 2.4 times (aOR: 2.4, 95% CI: 1.92-3.05). There was marginally significant association among households having a health insurance scheme and CHE (aOR 2.7; 95%CI 1.06–6.73).

For peer review only

For peer review only - http://bmjopen.bmj.com/site/about/guidelines.xhtml

Table 4. Independent determinants of CHE for TB and HIV care using a multivariate logistic regression model (Ethiopia)

**TB** Variable	aOR (95% CI)	P-value	**HIV** Variable	aOR (95% CI)	P-value
Frequency of OPD visits[¥]	2.4 (1.9-3.1)	<0.001	Frequency of visits Per year[¥]	1.07 (1.01-1.1)	0.021
Hospitalization			Hospitalization		
No	Ref.		No	Ref.	
Yes	30.6 (4.8-199.8)	0.001	Yes	3.3 (2.3-4.9)	<0.001
Income quintiles			Income quintiles		
Richest	Ref.		Richest	Ref.	
Rich[£]	4.1 (2.1-7.8)	<0.001	Rich[£]	2.1 (1.0-4.5)	0.051
Middle	4.9 (2.5-9.4)	<0.001	Middle	3.9 (1.9-7.9)	<0.001
Poor	7.0 (3.6-13.7)	<0.001	Poor	8.2 (4.2-15.8)	<0.001
Poorest	14.6 (7.5-28.3)	<0.001	Poorest	15.4 (8.0-29.5)	<0.001
Place of diagnosis					
Government	Ref.				
Private	2.6 (1.5-4.3)	<0.001			
TB/HIV co-infection					
No	Ref.				
Yes	3.2 (1.6-6.2)	0.001			
Insurance (i.e. CBHI)					
No	Ref.				
Yes	2.7 (1.1-6.7)	0.038			
Type of TB					
Bacteriologically confirmed TB	Ref.				
Clinically diagnosed TB	1.6 (1.0-2.8)	0.075			
Extra pulmonary TB[£]	2.6 (1.8-4.0)	<0.001			

¥ Variable treated as continuous
£ Overall test is significant
CBHI: Community based health insurance

For peer review only

For peer review only - http://bmjopen.bmj.com/site/about/guidelines.xhtml

DISCUSSION

In this study, we tried to estimate the OOP, total cost, incidence and determinants of CHE among individuals seeking HIV and TB care. This could provide valuable insight into the level of FRP that a health system offers to its population.

HIV care costs and CHE

In Ethiopia, where ART is given free-of-charge, PLHIV had to pay a total of $91 per year for HIV care ($23 per visit). The total HIV patient cost was equivalent to 8% of their annual income. As HIV requires lifelong care, such cost have devastating impact on affected households. Our estimate was lower than the level and rate found in Cameron 17% [38], Ethiopia 21% [20], Nepal 28.5% [39] and South Africa (30%) [40]. These variations might arise from different study settings [38, 39]; high expenses of additional food and time loss [40]. Similarly, more centralized HIV service delivery, delay in seeking care, and long distance travel to access services might explain the difference with the previous study from Ethiopia. Additionally, the annual income in this study was twice the latter study [20]. However, a study on outpatient HIV care in Nigeria reported one-fourth of the cost in this study ($21.76) [41].

In our study, the average direct OOP expenditure for HIV care was $ 63 (i.e. 68% of the total cost) and comparable with previous studies from Lao, Ethiopia and Nepal [16, 20, 39]. Diagnostics, medicines and transportation costs constitute the largest share, and may pause a serious challenge to the success of the HIV program. This calls for public financing policies (treatment free of charge for vulnerable groups) and decentralization in the next steps of UHC expansion, especially for diagnosis and treatment of HIV related comorbidities. The productivity loss identified here was one half of the report from pervious Ethiopia and Nepal study, [20, 39] but one fourth of that of Lao study [16]. The lower cost in our study may be due to the wider scale up of ART in recent years, which minimizes costs related to pre-ART care and management of advanced HIV infection. Both direct and indirect costs of HIV care increases as income rises.

For peer review only - http://bmjopen.bmj.com/site/about/guidelines.xhtml

The equity ratios (Q1:Q5, 0.39) showed that higher expenditure among the richest income quintile, which is consistent with a study from southeast Nigeria [42].

In our study, about a fifth (22%) of patients seeking HIV care experienced CHE. A study from India also depicts similar findings [43]. However, the rate is lower than a study from Cameroon [38]. Furthermore, the incidence of HIV related CHE remained relatively high; in particular, where poorest households suffer more. Similar socio-economic inequality was observed in previous studies, [41, 42] highlighting the importance of rendering equitable access to all in need of HIV care, particularly for the poor [38]. Consistent with previous studies, being poor is associated with higher CHE [16, 44]. In addition, the poor were pushed further beyond the CHE threshold than the better off. This is of great concern, as health shocks are slightly managed by the poor through reduction of basic requirements to compensate for HIV care. Similar to previous studies, being hospitalized was a stronger determinant of CHE [16, 41].

TB care costs and CHE

In our study, TB patients incurred a total cost of $115 per episode and represents 21% of the household annual income, comparable to a study conducted in South Africa (22%) [45]. However, the total cost was less than the figure reported by previous studies in Ethiopia [19, 28], but twice higher than the finding from southern Ethiopia [46]. The greatest variation arises from food supplementation expenses ($72 vs $25) [28], lost workdays, and differences in the valuation of time loss [19]. Furthermore, close to 85% of the study participant primarily seek diagnostic care from public health facilities that may have lowered patient cost. The average patient costs of DR-TB care were four times higher than drug susceptible TB care. However, the DR-TB cost was three times lower than findings from another Ethiopian study [47]. Still the devastating nature of DR-TB may put them at special risk of CHE. The lower costs was mainly due to the introduction of shorter DR-TB treatment regimen (9-12 month regimen), which, 71% of our study participants were taking this regimen.

For peer review only - http://bmjopen.bmj.com/site/about/guidelines.xhtml

We found that one-half of the mean TB cost was incurred before treatment initiation, which was consistent with many previous studies [11, 18, 19, 48, 49]. This is mainly due to payment while seeking care in the pathway to proper TB diagnosis [18]. In addition to the financial burden of high pre-treatment cost, it will be a barrier to complete the diagnostic process, and to timely access treatment and care. These stress the need for early case finding with rapid and point of care TB diagnostics, engagement of private care providers, and instituting effective referral and linkage [46].

The direct patient costs incurred constitutes 46% of the total, comparable with findings from elsewhere (36%) [50] and systematic review result (40%) [11]. However, the proportion was higher than the finding from southwestern Ethiopia (29%) and lower than the report from central Ethiopia (71%) [19, 28]. Consistent with previous studies, non-medical and indirect costs represent a large share of the TB cost, while medical cost represent less than 20% [11, 19, 40]. Therefore, ensuring affordable TB services within the framework of UHC calls for interventions to minimize diagnostic delays and FRP to mitigate TB related costs.

A higher percentage of households incur CHE for TB care (40%), which was comparable with studies from Fiji (40%), Ghana (47.6%), China (53%), Philippines (35%) and lower than reported rates from Ethiopia (63%), Nigeria (65%) and Benin (72%) [28, 48, 49, 51-53]. However, CHE for TB care was higher in this study compared to findings in studies from India (21%) and Malaysia (6%) [50, 54]. This variation might arise from cost estimation method, study setting, health system and socioeconomic differences. The TB related CHE was higher than the reported rate for HIV; this might be due to variation in population affected by HIV and TB and socio-economic differences. Similarly, the mean overshoot for TB was 6.3% and the MPO was 15.3%, both were similar with finding from Benin (7.8% and, 14.8%) and Nigeria (6.0% and, 9.3%) [49, 51]. TB, inequitably, imposes a greater burden of CHE on the poor households. Even though poor households tend to spend less, a higher share of their income is spent on seeking TB care

For peer review only - http://bmjopen.bmj.com/site/about/guidelines.xhtml

[12, 28, 45, 49, 51]. In addition, the excess CHE beyond the threshold was inequitably high among the poor. This finding is also in line with other studies [28, 49, 51]. Similar to other studies, hospitalization, income status, and TB/HIV coinfection were among the key determinants of TB related CHE [27, 48, 49, 51, 54]. In addition, even if the health insurance coverage (i.e.CBHI) was low and is limited to medical costs, we found marginally protective effect of the scheme against CHE [55]. However, health insurance per se does not alleviate the major TB costs.

Not surprisingly, TB and HIV patient use different coping strategies. Majority of TB patients adopt coping strategies compared to those with HIV. Furthermore, the type of coping strategies was not similar across income quintile. The finding was higher than a previous study, where 15% of HIV and 8% of TB patient adopted coping strategy [40].

Study limitation and strengths

This study has some limitations. First, the cost measurements relied on patient's ability to remember, which increases risk of recall bias. However, we have limited the recall bias through interview of participants who received HIV and TB care within the past one month, when patients have a better recollection of their pathway to care [27, 30]. Second, HIV costs may be overestimated when aggregated over one-year period. Third, our findings might not be representative of all PLHIV and TB patients in Ethiopia, as the sampling for HIV primarily consisted of urban, and excludes person not member of PLHIV association and also the TB study is limited to specific regions of the country. Moreover, undetected HIV and TB cases not seeking care were not addressed. Despite these limitations, we used a standard tool and method to conduct the study. We also estimated CHE using both narrower and broader definitions to enable comparison. We believe that our study will be of high value to inform policy, at national and sub-national levels, related to FRP of both diseases, which is central in achieving UHC.

For peer review only - http://bmjopen.bmj.com/site/about/guidelines.xhtml

To this end, despite OOP exemption of HIV and TB care services in Ethiopia, we found that there is a large gap between the actual level of financial protection provided and the ideal goal. Our findings highlight several policy implications. First, the introduction of more patient centred model of care with effective diagnostics is required in order to optimize HIV and TB service delivery. Second, strategies to ensure social and FRP for households with significant risk of CHE of both diseases is necessary. Furthermore, national HIV and TB programs should adopt public finance policies where equitable and quality services are provided free of OOP for all in need.

CONCLUSION

HIV and TB affected individuals and their households in Ethiopia face substantial costs in seeking care despite "free medical services". The incidence of CHE related to HIV and TB care were high in all income quintiles, though more so in the poorest households. Policy makers should introduce patient centred care; expand FRP and social protection measures to minimize the high patient cost of HIV and TB care, particularly among vulnerable populations.

Supplementary file

Supplementary appendix 1: Consumption construction, and measurement of CHE, findings

Availability of data and materials

The data supporting the conclusion of this study can be available upon reasonable request from the corresponding author.

Competing Interests

The authors declare that they have no competing interests.

Funding

This study was supported by Bill & Melinda Gates Foundation grant number OPP1162384.

For peer review only - http://bmjopen.bmj.com/site/about/guidelines.xhtml

Contributors

LFA, KAJ, MTT: conception and design of the study. LFA: acquisition of data, data analysis, and drafted the paper. LFA, AJ, KAJ, MTT, ENH: critically reviewed the paper and gave approval for the final version to be published.

Acknowledgments

The authors would like to thank all HIV and TB patients and supervisors who participated in this study. We thank all stakeholders, the Ministry of Health, Ethiopia, Ethiopian public health institute, Oromia and Afar regional health Bureau with their respective districts for making this research possible.

Figure 1. Pen's parade of household income gross and net of payments for HIV (a) and TB care (b) (Ethiopia).

Figure 2. Interval plot (with 95% confidence interval for the mean) of incidence and intensity of HIV and TB-related catastrophic health expenditures (CHE) across income quintiles, using 10% threshold (Ethiopia).

Figure 3. Concentrations curves and index (I) for direct, indirect, total cost and catastrophic health expenditures (CHE) for (a) HIV and (b) TB services in Ethiopia.

For peer review only

For peer review only - http://bmjopen.bmj.com/site/about/guidelines.xhtml

REFERENCES

1. Vitoria M, Granich R, Gilks CF, et al. The global fight against HIV/AIDS, tuberculosis, and malaria: current status and future perspectives. American journal of clinical pathology. 2009;131(6):844-8.
2. Danforth K, Granich R, Wiedeman D, et al. Global Mortality and Morbidity of HIV/AIDS. 2017:29-44.
3. Bloom BR, Atun R, Cohen T, et al. Tuberculosis. 2017:233-313.
4. World Health Organization. Global tuberculosis report 2019. Geneva: World Health Organization; 2019.
5. World Health Organization. WHO fact sheet on HIV/AIDs. 25 July 2019: Available from:https://www.who.int/news-room/fact-sheets/detail/hiv-aids.
6. UNAIDS. UNAIDS data 2019. 16 JULY 2019, Available from https://www.unaids.org/en/resources/documents/2019/2019-UNAIDS-data.
7. WHO. IMPLEMENTING THE END TB STRATEGY: THE ESSENTIALS Geneva: World Health Organization; 2015.
8. Babigumira JB, Stergachis A, Veenstra DL, et al. Potential cost-effectiveness of universal access to modern contraceptives in Uganda. PLoS One. 2012;7(2):e30735.
9. Federal Democratic Republic of Ethiopia Ministry of Health. September 2019.Ethiopia Health Accounts, 2016/17. Addis Ababa, Ethiopia.
10. The Federal Democratic Republic of Ethiopia Ministry of Health. Health Sector Transformation Plan 2015/16 - 2019/2020(2008-2012 EFY). Addis Ababa, Ethiopia.2015.
11. Tanimura T, Jaramillo E, Weil D, et al. Financial burden for tuberculosis patients in low- and middle-income countries: a systematic review. The European respiratory journal. 2014;43(6):1763-75.
12. Russell S. The economic burden of illness for households in developing countries: a review of studies focusing on malaria, tuberculosis, and human immunodeficiency virus/acquired immunodeficiency syndrome. The American journal of tropical medicine and hygiene. 2004;71(2 Suppl):147-55.
13. Wagstaff A, van Doorslaer E. Catastrophe and impoverishment in paying for health care: with applications to Vietnam 1993-1998. Health economics. 2003;12(11):921-34.
14. O'Donnell O, Van Doorslaer E, Wagstaff A, et al. Analyzing Health Equity Using Household Survey Data A Guide to Techniques and Their Implementation. 2012.
15. Xu K, Evans DB, Kawabata K, et al. Household catastrophic health expenditure: a multicountry analysis. Lancet (London, England). 2003;362(9378):111-7.
16. Barennes H, Frichittavong A, Gripenberg M, et al. Evidence of High Out of Pocket Spending for HIV Care Leading to Catastrophic Expenditure for Affected Patients in Lao People's Democratic Republic. PLOS ONE. 2015;10(9):e0136664.
17. Long Q, Smith H, Zhang T, et al. Patient medical costs for tuberculosis treatment and impact on adherence in China: a systematic review. BMC Public Health. 2011;11(1):393.
18. Wingfield T, Boccia D, Tovar M, et al. Defining catastrophic costs and comparing their importance for adverse tuberculosis outcome with multi-drug resistance: a prospective cohort study, Peru. PLoS medicine. 2014;11(7):e1001675.
19. Asres A, Jerene D, Deressa W. Pre- and post-diagnosis costs of tuberculosis to patients on Directly Observed Treatment Short course in districts of southwestern Ethiopia: a longitudinal study. Journal of health, population, and nutrition. 2018;37(1):15.
20. Vassall A, Seme A, Compernolle P, et al. Patient costs of accessing collaborative tuberculosis and human immunodeficiency virus interventions in Ethiopia. The international journal of tuberculosis and lung disease : the official journal of the International Union against Tuberculosis and Lung Disease. 2010;14(5):604-10.

For peer review only - http://bmjopen.bmj.com/site/about/guidelines.xhtml

21. Mauch V, Bonsu F, Gyapong M, et al. Free tuberculosis diagnosis and treatment are not enough: patient cost evidence from three continents. The international journal of tuberculosis and lung disease : the official journal of the International Union against Tuberculosis and Lung Disease. 2013;17(3):381-7.
22. Bhojani U, Thriveni B, Devadasan R, et al. Out-of-pocket healthcare payments on chronic conditions impoverish urban poor in Bangalore, India. BMC Public Health. 2012;12:990.
23. Ministry of Health, Ethiopia. Annual health sector performance report 2011 EFY (2018/19). Addis Ababa, Ethiopia.
24. Kawabata K, Xu K, Carrin G. Preventing impoverishment through protection against catastrophic health expenditure. Bulletin of the World Health Organization. 2002;80(8):612.
25. Lopera MM, Einarson TR, Ivan Bula J. Out-of-pocket expenditures and coping strategies for people living with HIV: Bogota, Colombia, 2009. AIDS care. 2011;23(12):1602-8.
26. World Health Organization. Health Financing: Monitoring Sustainable Development Goals—Indicator 3.8.2; World Health Organization: Geneva, Switzerland, 2019. Available from: https://www.who.int/health financing/topics/financial-protection/monitoring-sdg/en/ (accessed on 5 September 2019).
27. Ukwaja KN, Alobu I, lgwenyi C, et al. The High Cost of Free Tuberculosis Services: Patient and Household Costs Associated with Tuberculosis Care in Ebonyi State, Nigeria. PLOS ONE. 2013;8(8):e73134.
28. Getahun B, Wubie M, Dejenu G, et al. Tuberculosis care strategies and their economic consequences for patients: the missing link to end tuberculosis. Infectious Diseases of Poverty. 2016;5(1):93.
29. Ministry of Health, Ethiopia. Health Management Information System (HMIS) annual TB and Leprosy report 2016. Addis Ababa, Ethiopia.
30. WHO. WHO Global TB Programme. Protocol for survey to determine direct and indirect costs due to TB and to estimate proportion of TB-affected households experiencing catastrophic costs. November 2015.
31. Federal Democratic Republic of Ethiopia Ministry of Health. August 2017. Health Service Utilization and Expenditure Survey among People Living with HIV (PLHIV), 2013/14, Addis Ababa, Ethiopia.
32. National Bank of Ethiopia. Inter-bank daily foreign exchange rate in (USD)—archive. Available from: National Bank of Ethiopia; 2019. https://www.nbe.gov.et/market/dailyexchange.html.
33. International Monetary Fund.World Economic Outlook Database October 2015. Available from: https://wwwimforg/external/pubs/ft/weo/2019/02/weodata/indexaspx.
34. WHO Global TB Programme. Protocol for survey to determine direct and indirect costs due to TB and to estimate proportion of TB-affected households experiencing catastrophic costs. November 2015.
35. O'Donnell OvD, Eddy Wagstaff, Adam Lindelow, Magnus. Analyzing Health Equity Using Household Survey Data.
36. O'Donnell O, O'Neill S, Van Ourti T, et al. conindex: Estimation of concentration indices. The Stata journal. 2016;16(1):112-38.
37. L. Gortmaker S, Hosmer D, Lemeshow S. Applied Logistic Regression. Contemporary Sociology. 1994;23:159.
38. Boyer S, Abu-Zaineh M, Blanche J, et al. Does HIV services decentralization protect against the risk of catastrophic health expenditures?: some lessons from Cameroon. Health services research. 2011;46(6pt2):2029-56.
39. Poudel AN, Newlands D, Simkhada P. The economic burden of HIV/AIDS on individuals and households in Nepal: a quantitative study. BMC health services research. 2017;17(1):76.

For peer review only - http://bmjopen.bmj.com/site/about/guidelines.xhtml

40. Mudzengi D, Sweeney S, Hippner P, et al. The patient costs of care for those with TB and HIV: a cross-sectional study from South Africa. Health policy and planning. 2017;32(suppl_4):iv48-iv56.
41. Etiaba E, Onwujekwe O, Torpey K, et al. What Is the Economic Burden of Subsidized HIV/AIDS Treatment Services on Patients in Nigeria and Is This Burden Catastrophic to Households? PLoS One. 2016;11(12):e0167117.
42. Onwujekwe O, Dike N, Chukwuka C, et al. Examining catastrophic costs and benefit incidence of subsidized antiretroviral treatment (ART) programme in south-east Nigeria. Health policy (Amsterdam, Netherlands). 2009;90(2-3):223-9.
43. Shukla M, Agarwal M, Singh J, et al. Catastrophic health expenditure amongst people living with HIV/AIDs availing antiretroviral treatment services at two tertiary care health facilities in district of northern India. National Journal of Community Medicine. 2015;6:323-8.
44. Mnzava T, Mmari E, Berruti A. Drivers of Patient Costs in Accessing HIV/AIDS Services in Tanzania. Journal of the International Association of Providers of AIDS Care. 2018;17:2325958218774775.
45. Foster N, Vassall A, Cleary S, et al. The economic burden of TB diagnosis and treatment in South Africa. Social Science & Medicine. 2015;12.
46. Datiko DG, Lindtjørn B. Cost and Cost-Effectiveness of Treating Smear-Positive Tuberculosis by Health Extension Workers in Ethiopia: An Ancillary Cost-Effectiveness Analysis of Community Randomized Trial. PLOS ONE. 2010;5(2):e9158.
47. Berhanu RH, Schnippel K, Kularatne R, et al. Can patients afford the cost of treatment for multidrug-resistant tuberculosis in Ethiopia? The international journal of tuberculosis and lung disease : the official journal of the International Union against Tuberculosis and Lung Disease. 2018;22(8):358-62.
48. Duan W, Zhang W, Wu C, et al. Extent and determinants of catastrophic health expenditure for tuberculosis care in Chongqing municipality, China: a cross-sectional study. BMJ Open. 2019;9(4):e026638.
49. Ukwaja KN, Alobu I, Abimbola S, et al. Household catastrophic payments for tuberculosis care in Nigeria: incidence, determinants, and policy implications for universal health coverage. Infect Dis Poverty. 2013;2(1):21.
50. Atif M, Sulaiman SA, Shafie AA, et al. Resource utilization pattern and cost of tuberculosis treatment from the provider and patient perspectives in the state of Penang, Malaysia. BMC health services research. 2014;14:353.
51. Laokri S, Dramaix-Wilmet M, Kassa F, et al. Assessing the economic burden of illness for tuberculosis patients in Benin: determinants and consequences of catastrophic health expenditures and inequities. Tropical medicine & international health : TM & IH. 2014;19(10):1249-58.
52. Viney K, Islam T, Hoa NB, et al. The Financial Burden of Tuberculosis for Patients in the Western-Pacific Region. Tropical medicine and infectious disease. 2019;4(2).
53. Pedrazzoli D, Siroka A, Boccia D, et al. How affordable is TB care? Findings from a nationwide TB patient cost survey in Ghana. Tropical medicine & international health : TM & IH. 2018;23(8):870-8.
54. Prasanna T, Jeyashree K, Chinnakali P, et al. Catastrophic costs of tuberculosis care: a mixed methods study from Puducherry, India. Global health action. 2018;11(1):1477493.
55. Zhou C, Long Q, Chen J, et al. The effect of NCMS on catastrophic health expenditure and impoverishment from tuberculosis care in China. International Journal for Equity in Health. 2016;15(1):172.

For peer review only - http://bmjopen.bmj.com/site/about/guidelines.xhtml

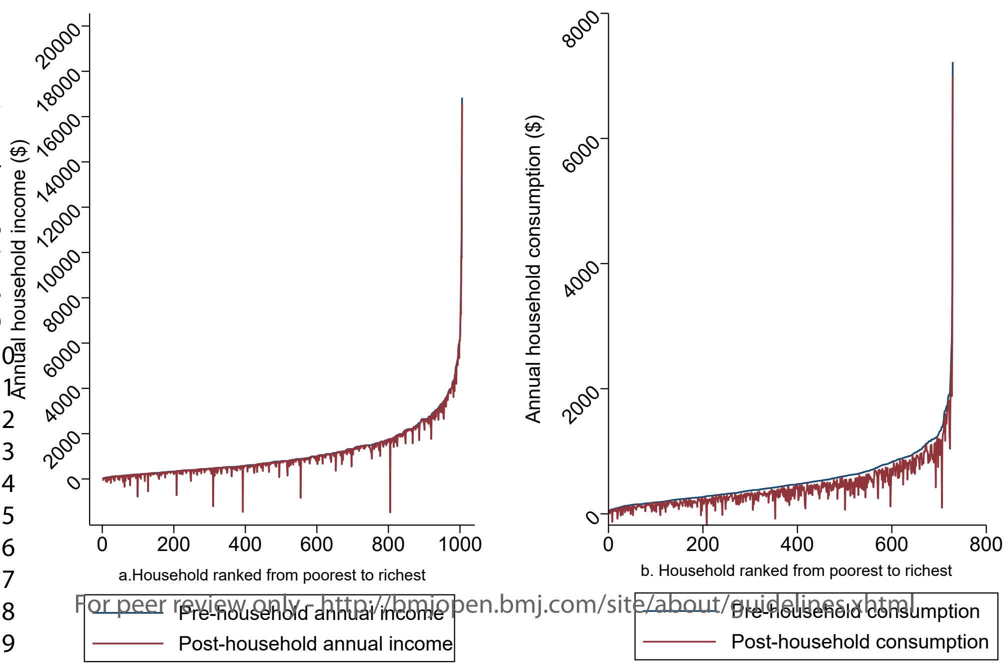

For peer review only - http://bmjopen.bmj.com/site/about/guidelines.xhtml

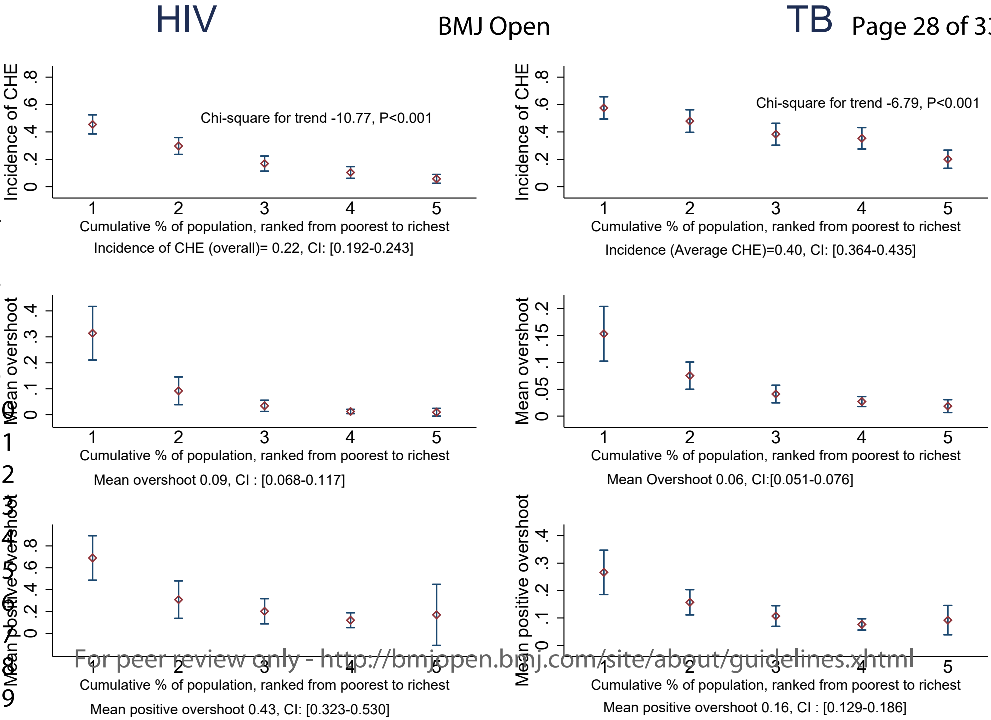

For peer review only - http://bmjopen.bmj.com/site/about/guidelines.xhtml

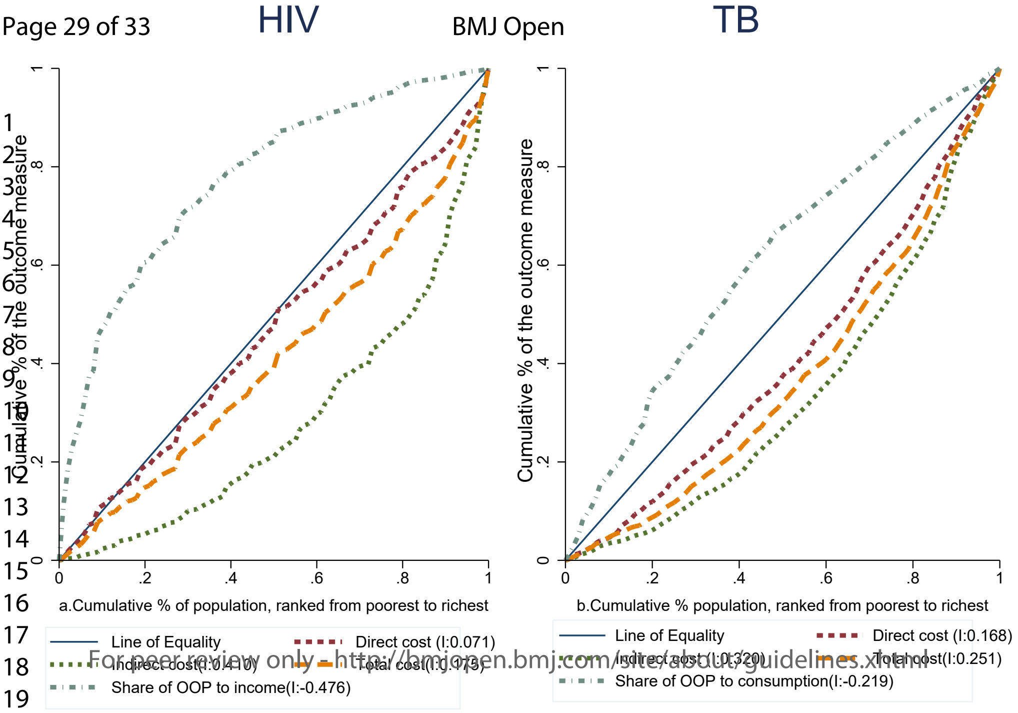

For peer review only - http://bmjopen.bmj.com/site/about/guidelines.xhtml

Supplementary appendix 1

The financial burden of HIV and TB among patients in Ethiopia: a cross-sectional survey

Lelisa Fekadu[1]*, Eyerusalem Negussie[2], Abdulrahman Jbaily[3], Mieraf Taddesse Tolla[3], Kjell Arne Johansson[1]

For peer review only

S1. Consumption construction

In this study, income (for HIV) and consumption as proxy for income (for tuberculosis) were used as household welfare measure. The consumption aggregates for tuberculosis (TB) was constructed using the overall value of the food (purchased food, home produced food) items, non-food items, and housing information collected at different recall periods (ranging from 1 to 12 months depending on how frequently the item purchased). Then, all reported expenditures on food items, non-food items, and housing were converted to a month period, then added up and multiplied by 12 to create annual consumption for each household.

The income (for HIV) and consumption (for TB) measures were also adjusted for family size and demographic composition to reflect economy of scale. We constructed per adult equivalence through dividing household income/consumption expenditure by an adult equivalent scale. Adult equivalent values (AE) were calculated using $AE = (A + \alpha K)^{\theta}$ for HIV, where A stands for number of adults, K is the number of children in the household, α is the cost of a child relative to an adult (0.33) and θ is the degree of economies of scale (0.9) (1). Whereas for TB, due to the unavailability of child data, we calculated using $AE = hsize^{\beta}$, where “$hsize$” is the actual household size and β was set to be 0.56 (2). Using per adult equivalence income (HIV) and consumption (TB) values, we grouped all households into five income quintiles of equal size.

For peer review only - http://bmjopen.bmj.com/site/about/guidelines.xhtml

S2. Incidence and intensity of catastrophic health expenditure (CHE)

S2.1. Measuring incidence of CHE (Headcount)

Catastrophic health expenditure (headcount, H) occurs when household OOP health spending exceeds a predefined threshold (10%) of household income/consumption. Furthermore, we conducted analysis at 20% threshold of both OOP and total cost (new definition of catastrophic cost recommended for TB), and at 40% of non-food expenditure (i.e. net of basic subsistence expenditure).

The CHE headcount is calculated using the equation

$$H = \frac{1}{N}\sum_{i=1}^{N} E_i \,,$$

$$\text{Where,} \quad E_i = 1 \;\text{ if }\; \frac{T_i}{X_i} \;\text{ or }\; \frac{T_i}{(X_i - FE_i)} > z \;\text{ and 0 otherwise.}$$

Here, N equals the total sample size, T_i is the OOP health spending of household i, X_i is the total expenditure of household i, FE_i is the food expenditure of household i and z is the specified threshold.

S2.2. Measuring intensity of CHE

The intensity is measured using overshoot (O) and mean positive overshoot (MPO).

S2.2.1. Overshoot

The overshoot measures the extent of average expenditure exceeding the given threshold in the entire sample. The overshoot is calculated using the equation

$$O = \frac{1}{N}\sum_{i=1}^{N} (E_i\left(\frac{Ti}{Xi}\right) - z)$$

S2.2.2. Mean positive overshoot

For peer review only - http://bmjopen.bmj.com/site/about/guidelines.xhtml

The mean positive overshoot measures the extent of an average expenditure exceeding the threshold among households experiencing CHE, and is calculated by:

$$MPO = \frac{O}{H}$$

Table A1. Incidence and intensity of CHE for HIV and TB across different income quintiles and threshold, 2019, Ethiopia

Disease	Measure Of CHE	Threshold	Average	Income Quantile				
				Q1	Q2	Q3	Q4	Q5
**HIV**	Out-of-pocket HIV spending: as a share of annual income							
	Head count (CHE incidence) %	10%	22	46	30	17	10	6
	Overshoot %	10%	9.3	31.4	9.2	3.4	1.3	1.0
	Mean positive overshoot %	10%	42.7	69.0	30.9	20.3	12.1	17.1
	Out-of-pocket HIV spending: as a share of annual income							
	Head count (CHE incidence) %	20%	13	36	17	9	3	2
	Overshoot %	20%	7.6	27.3	7.0	2.2	0.6	0.7
	Mean positive overshoot %	20%	58.0	77.1	40.8	25.4	24.7	49.6
	Total HIV spending: as a share of annual income							
	Head count (CHE incidence) %	20%	17.2	42	20	12	8	4
	Overshoot %	20%	8.9	31.0	8.1	3.2	1.2	1.1
	Mean positive overshoot %	20%	52.0	73.9	39.7	28.1	14.7	27.5
	Out-of-pocket HIV spending: as a share of total non-food expenditure							
	Head count (CHE incidence) %	40%	13	36	18	9	4	2
	Overshoot %	40%	16.1	57.6	14.9	4.8	1.3	1.5
	Mean positive overshoot %	40%	120	162	84.5	54.5	38.2	105
**TB**	Out-of-pocket TB spending: as a share of annual income							
	Head count (CHE incidence) %	10%	40	58	48	38	35	20
	Overshoot %	10%	6.3	15.8	7.5	4.1	2.7	1.9
	Mean positive overshoot %	10%	15.3	26.6	15.7	10.7	7.6	9.2
	Out-of-pocket TB spending: as a share of annual income							
	Head count (CHE incidence) %	20%	17	38	23	10	8	6
	Overshoot %	20%	3.7	10.7	4.1	1.9	0.7	0.8
	Mean positive overshoot %	20%	21.3	27.8	17.7	18.7	8.8	15.2
	Total TB spending: as a share of annual income							
	Head count (CHE incidence) %	20%	48	60	53	48	46	36
	Overshoot %	20%	10.0	18.6	11.0	8.0	6.7	4.4
	Mean positive overshoot %	20%	20.0	31.1	20.9	16.7	14.7	12.3
	Out-of-pocket HIV spending: as a share of total non-food expenditure							
	Head count (CHE incidence) %	40%	37	50	43	39	33	19
	Overshoot %	40%	29.2	73.0	27.5	18.6	14.9	11.6
	Mean positive overshoot %	40%	78.7	146	63.6	47.7	44.7	59.5
	Where, Q1 = Poorest; Q2 = Poorer; Q3 = Middle; Q4 = Richer; Q5 = Richest.							

For peer review only - http://bmjopen.bmj.com/site/about/guidelines.xhtml

Table A2. Mean (median) patient costs per TB case across three types of TB (Ethiopia).

Income quintiles	Type of TB					
	Pulmonary[¥]		Extra-pulmonary TB[¥]		Drug resistant-TB	
	Mean (SD)	Median (IQR)	Mean (SD)	Median (IQR)	Mean (SD)	Median (IQR)
**Poorest**	46 (43)	32 (13-62)	60 (45)	46 (32-75)	295 (290)	139 (65-607)
**Poor**	67 (44)	56 (34-85)	107 (90)	83 (49-136)	159 (85)	153 (68-199)
**Middle**	102 (80)	75 (48-137)	115 (74)	94 (72-131)	205 (175)	156 (86-250)
**Rich**	133 (99)	102 (65-176)	155 (86)	133 (79-212)	534 (671)	291 (131-669)
**Richest**	175 (165)	121 (78-216)	262 (226)	212 (112-294)	971 (1356)	610 (201-821)
**Total***	104 (107)	73 (41-135)	140 (138)	96 (58-180)	446 (732)	191 (107-607)

* kruskal wallis test p-value <0.001

¥ CHE at 10% threshold ranged from 33% to 57% for pulmonary and extra-pulmonary TB, respectively.

Figure A1. Average total cost of HIV and TB care across income quintiles in Ethiopia.

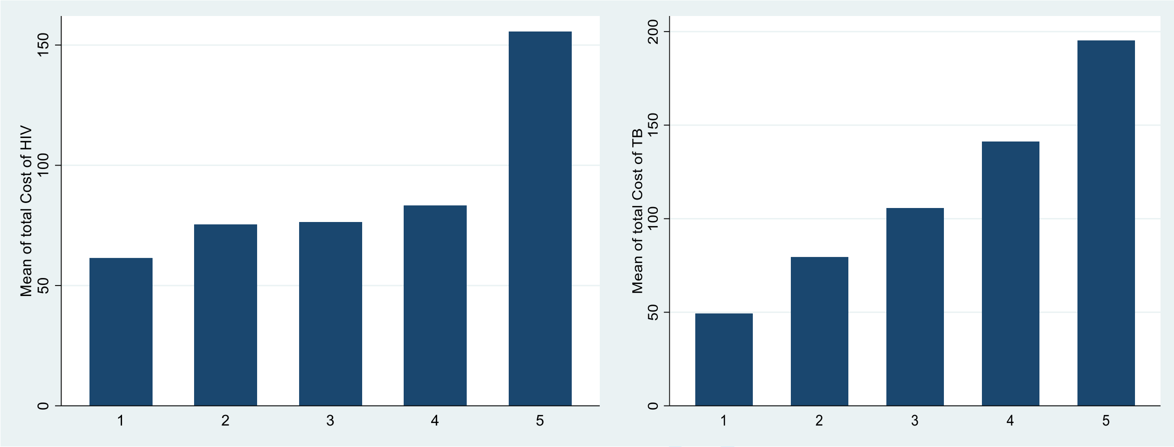

Figure A2. Out-of-pocket payment share curve for HIV and TB in Ethiopia.

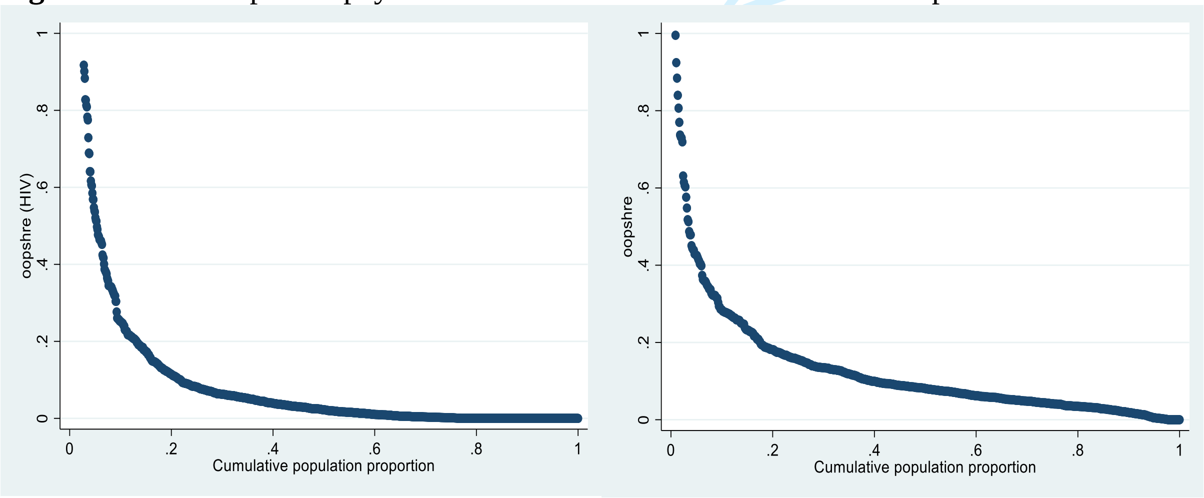

For peer review only - http://bmjopen.bmj.com/site/about/guidelines.xhtml

Figure A3. Receiver Operating Characteristic curve (ROC plot) for the prediction power of the HIV and TB model.

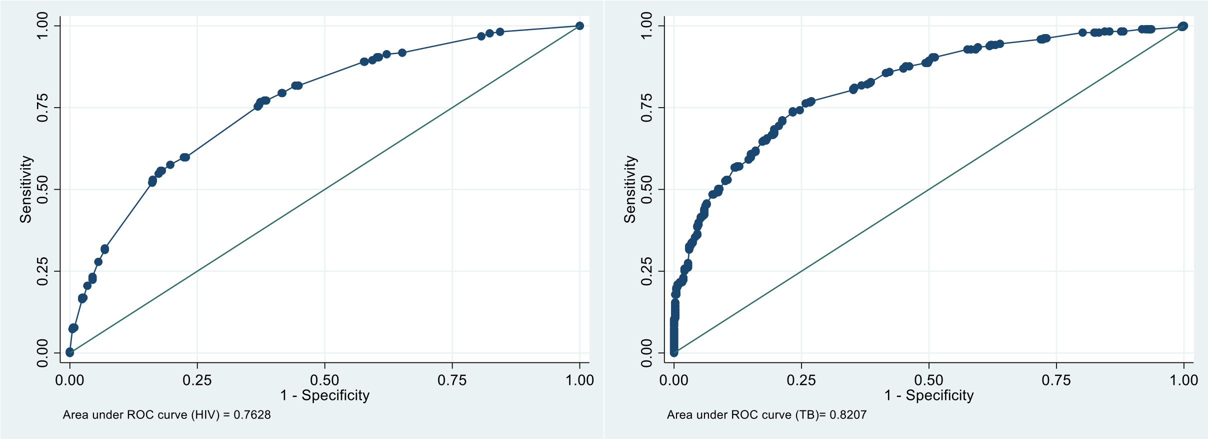

References

1. Deaton A, Zaidi S. Guidelines for constructing consumption aggregates for welfare analysis. 2002.
2. Xu K, Evans DB, Kawabata K, Zeramdini R, Klavus J, Murray CJ. Household catastrophic health expenditure: a multicountry analysis. Lancet (London, England). 2003;362(9378):111-7.

For peer review only

For peer review only - http://bmjopen.bmj.com/site/about/guidelines.xhtml

The financial burden of HIV and TB among patients in Ethiopia: a cross-sectional survey

Journal:	*BMJ Open*
Manuscript ID	bmjopen-2020-036892.R1
Article Type:	Original research
Date Submitted by the Author:	03-Mar-2020

Complete List of Authors:	Assebe, Lelisa; University of Bergen Faculty of Medicine and Dentistry, Global Public Health and Primary Care Medicine; Ethiopia Ministry of Health, Disease Prevention and control<br>Negussie, Eyerusalem; Ethiopia Ministry of Health, Disease prevention and control program<br>Jbaily, Abdulrahman; Harvard University T H Chan School of Public Health, Global Health and Population<br>Tolla, Mieraf Taddesse; Harvard University T H Chan School of Public Health,Global Health and Population<br>Johansson, Kjell Arne; University of Bergen Faculty of Medicine and Dentistry, Department of Global Public Health and Primary care

**Primary Subject Heading**:	Health economics
Secondary Subject Heading:	Public health
Keywords:	Tuberculosis < INFECTIOUS DISEASES, HIV & AIDS < INFECTIOUS DISEASES, HEALTH ECONOMICS

SCHOLARONE™ Manuscripts

I, the Submitting Author has the right to grant and does grant on behalf of all authors of the Work (as defined in the below author licence), an exclusive licence and/or a non-exclusive licence for contributions from authors who are: i) UK Crown employees; ii) where BMJ has agreed a CC-BY licence shall apply, and/or iii) in accordance with the terms applicable for US Federal Government officers or employees acting as part of their official duties; on a worldwide, perpetual, irrevocable, royalty-free basis to BMJ Publishing Group Ltd ("BMJ") its licensees and where the relevant Journal is co-owned by BMJ to the co-owners of the Journal, to publish the Work in this journal and any other BMJ products and to exploit all rights, as set out in our licence.

The Submitting Author accepts and understands that any supply made under these terms is made by BMJ to the Submitting Author unless you are acting as an employee on behalf of your employer or a postgraduate student of an affiliated institution which is paying any applicable article publishing charge ("APC") for Open Access articles. Where the Submitting Author wishes to make the Work available on an Open Access basis (and intends to pay the relevant APC), the terms of reuse of such Open Access shall be governed by a Creative Commons licence – details of these licences and which Creative Commons licence will apply to this Work are set out in our licence referred to above.

Other than as permitted in any relevant BMJ Author's Self Archiving Policies, I confirm this Work has not been accepted for publication elsewhere, is not being considered for publication elsewhere and does not duplicate material already published. I confirm all authors consent to publication of this Work and authorise the granting of this licence.

For peer review only

Title

The financial burden of HIV and TB among patients in Ethiopia: a cross-sectional survey

Lelisa Fekadu Assebe[1]*, Eyerusalem Kebede Negussie[2], Abdulrahman Jbaily[3], Mieraf Taddesse Tolla[3], Kjell Arne Johansson[1]

1. Department of Global Public Health and Primary Care Medicine, University of Bergen, Bergen, Norway

2. Disease prevention and control program, Ministry of Health, Addis Ababa, Ethiopia

3. Department of Global Health and Population, Harvard T.H. Chan School of Public Health, Harvard University, Boston, MA, USA

*Correspondence: lelfekadu1@gmail.com

For peer review only

For peer review only - http://bmjopen.bmj.com/site/about/guidelines.xhtml

ABSTRACT

Objectives: Human Immunodeficiency Virus (HIV) and Tuberculosis (TB) are major global health threats and can result in household financial hardships. Here, we aim to estimate the household economic burden and the incidence of catastrophic health expenditures (CHE) incurred by HIV and TB care across income quintiles in Ethiopia.

Design: A cross-sectional survey.

Setting: 27 health facilities of Afar and Oromia regions for TB, and nationwide household survey for HIV.

Participants: A total of 1,006 and 787 individuals seeking HIV and TB care were enrolled, respectively.

Outcome measures: The economic burden (i.e. direct and indirect cost) of HIV and TB care was estimated. In addition, the CHE incidence and intensity was determined using direct costs exceeding 10% of the household income threshold.

Results: The mean (SD) age of HIV and TB patient was 40 (10), and 30 (14) years, respectively. The mean (SD) patient cost of HIV was $78 ($170) per year and $115 ($118) per TB episode. Out of the total cost, the direct cost of HIV and TB constituted 69% and 46%, respectively. The mean (SD) indirect cost was $24 ($66) per year for HIV and $63 ($83) per TB episode. The incidence of CHE for HIV was 20%; ranges from 43% in the poorest to 4% in the richest income-quintile (P <0.001). Similarly, for TB, the CHE incidence was 40% and ranged between 58% and 20% among the poorest and richest income-quintiles respectively (P <0.001). This figure was higher for drug resistant TB (62%).

Conclusions: HIV and TB are causes of substantial economic burden and CHE, inequitably, affecting those in the poorest income quintile. Broadening the health policies to encompass interventions that reduce the high cost of HIV and TB care, particularly for the poor, is urgently needed.

For peer review only

For peer review only - http://bmjopen.bmj.com/site/about/guidelines.xhtml

Strengths and limitations of this study

- This study will be of high value to policies aimed at universal health coverage for HIV and TB care in Ethiopia due to the financial risk of seeking care.
- Patient costs from this study can provide empirical bases for national HIV and TB programs for adopting public finance or health insurance-based financing strategies.
- The patient cost for outpatient and inpatient HIV and TB care are presented together with income and consumption levels of households.
- The cost measurements relied on a patient's ability to remember, which increases risk of recall bias.
- The HIV sampling consisted primarily of urban population and the TB study was limited to specific regions of the country, therefore, these samples are not necessarily nationally representative.

review only

For peer review only - http://bmjopen.bmj.com/site/about/guidelines.xhtml

BACKGROUND

HIV and TB are major global health threats that cause a large financial burden on vulnerable populations. Global efforts, in the past two decades, have improved access to lifesaving HIV and TB interventions[1-3]. More than 72 million lives have been saved between 2000 – 2018[4, 5]. Nevertheless, high disease burden, inequality in utilisation of healthcare and service quality issues still exists[1-3]. Better understanding of factors that affect use of these services would help countries to achieve universal health coverage (UHC) of HIV and TB services.

The population in need for care is still large. Globally, 1.7 million people acquired HIV infection and 10 million new TB cases occurred in 2018. In the same period, more than 2 million people died from HIV and TB[4, 6]. In Ethiopia, the prevalence of HIV among adults was 1% (confidence interval (CI): 0.7%-1.4%) and the incidence rate of TB was 151/100,000 in 2018[4, 6]. In order to end HIV and TB, a comprehensive approach should include medical and non-medical interventions such as socio-economic support and poverty alleviation[7, 8].

Although many countries, including Ethiopia, offer ''free'' HIV and TB services, the implemented policies do not adequately provide realistic financial risk protection (FRP). The health budget in Ethiopia is low ($33.2 per capita) and mainly financed by out-of- pocket payment (OOP), which account for 31% of overall health finance[9, 10]. Hence, patient and their families, often face both direct and indirect costs, which create financial burden on households[11, 12]. A systematic review showed that individuals in low income countries spend a mean direct cost of $155 per drug susceptible TB (DS) and $406 per drug-resistant TB (DR-TB). The productivity losses were 2-3 times higher than the direct costs of DS and DR-TB cases, respectively[13]. Similarly, for HIV, spending ranges from $95 - $2,672 in sub-Saharan Africa[12]. Such high costs are related with catastrophic health expenditure (CHE), which occurs when the OOP exceeds 10% of annual income[14, 15] or 40% of household non-food expenditure[16]. In addition, OOP expenses for HIV and TB care may crowd out consumption

For peer review only - http://bmjopen.bmj.com/site/about/guidelines.xhtml

of basic needs and leave vulnerable households in debt/impoverishment[12, 17, 18]. Furthermore, high levels of patient cost may affect access to care, and lead to poor treatment outcome and prolonged period with infection[12, 14, 19-21].

Many factors may lead to CHE; exemptions of HIV and TB services often applies to limited aspects of the basic care package (e.g. $CD_{4,}$ viral load, AFB microscopy/Gene X-pert tests), treatment (e.g. antiretroviral therapy (ART), anti TB drugs). For example, not for pre-diagnostic tests (i.e. X-rays, haematology), adverse event screening, non-HIV and TB medication, hospitalization, transportation, food, lodging, and lost working days, which patients pay for[19, 22-24]. In addition, unavailability of diagnostic services in public health facilities pushes patients to seek care from expensive private providers[22]. Furthermore, low health insurance coverage (around 24% as of 2019), and repeated follow up visits were important contributing financial risk factors[25, 26]. It is also imperative that HIV and TB programs, needs to monitor household protection from CHE (i.e. financial risk protection) and its distribution across income groups (equity), as OOP health spending places greater burdens on the poor[27, 28].

In Ethiopia, few studies have been evaluating the extent of patient cost due to seeking HIV and TB care. The costs related to severe forms of the diseases and assessments across income or consumption groups were lacking from the studies reviewed[22, 23, 29]. Furthermore, none studied predictors of CHE[22, 23, 29]. Because HIV and TB are chronic diseases and intimately linked, it is reasonable to look at them jointly. This paper aims to estimate the economic burden and incidence of CHE incurred by standard HIV and TB care across income or consumption quintile among Ethiopian households. Moreover, we will assess factors associated with CHE for HIV and TB.

For peer review only - http://bmjopen.bmj.com/site/about/guidelines.xhtml

METHODS

Study setting and population

In this study, a nationwide household survey for HIV[10] and a cross sectional health facility based survey for TB, were used to estimate direct and indirect costs, and CHE.

Data for HIV were collected from mid-September to mid-October 2016. The total estimated number of PLHIV were 722,747 in Ethiopia. However, for TB, data were collected from December 2018 to September 2019 in three zones (i.e. zone 3 of Afar region, and Jimma and Adama special zones of Oromia region). The three zones were purposely selected and represents 4 million people mirroring the country's geographic and socio-economic heterogeneity. The zones account for 10% and 13% of the TB cases in Oromia and Afar regions (and 6% of the national prevalence), respectively.

Sample size and sampling technique

For HIV, PLHIV associations were used as a sampling frame to select HIV participants, as there is no national registry of PLHIV. The association operates in big cities and its members were mainly urban residents. The estimated sample size was 4,200. The response rate ranges from 92% to 100% across regions. A two-stage stratified cluster-sampling method was employed. In stage one, a sample of 105 HIV associations from a total of 588 were randomly selected and allocated to each region using probability proportional to the size. In stage two, 40 HIV members from each sampled association, in total 4,171, were randomly selected and interviewed. Among the study participants (i.e. 4,171), 1,006 had HIV related care and were considered in our analysis.

For TB, the sample size was calculated using two-population proportion formula with 80% power, 5% type I error, 95% confidence level, and using 39%[21] of households incur CHE among the richest income quartile; and to detect 15% point difference revealed 186 samples for each quartile. The final sample size with 10% non-response rate was 818 (of which 7% were DR-

For peer review only - http://bmjopen.bmj.com/site/about/guidelines.xhtml

TB). Systematic random sampling was employed to select 27 public health facilities from the three zones. The total sample size was distributed proportional to the TB case load[30].

Patient and public involvement

The research question of this study is in line with the Ethiopian tuberculosis research plan developed through multiple consultative processes involving broader stakeholders including patient representatives. We plan to disseminate the research findings through the national TB research conferences involving researchers, policy makers, stakeholders and affected communities.

Data collection tools and quality assurance

The data collection was based on a structured questionnaire adapted from the United States Agency for International Development (USAID) for HIV and the World Health Organization (WHO) patient costing tool for TB[31, 32]. The questionnaire captures sociodemographic variables, direct cost, indirect cost, productivity loss, assets, income, consumptions and coping related information. We complemented clinical information for TB through review of medical records. The questionnaire was translated into local languages, pre-tested and modified accordingly. Trained data collectors under close supervision undertook the face-to-face interview. TB patients with a minimum of one month on treatment were interviewed consecutively and the expenses were reported retrospectively. This timing is selected because patients' easily recall their pathway to TB care and would acquire adequate cost experience[28, 31]. Whereas, for HIV, the expenditure in the past four weeks for outpatient and six months for inpatient care were gathered.

Patient cost

The costs of seeking standard HIV and TB care were estimated from patient perspective. The expenditure related to routine care, HIV-related opportunistic infection, and managing co-

For peer review only - http://bmjopen.bmj.com/site/about/guidelines.xhtml

morbidities was considered for HIV. Likewise, for TB episode, the expenditure in the pathway to care from onset of symptoms, diagnosis and completion of treatment was included.

Direct costs were household expenditures for medical (i.e. registration/consultation fees, laboratory tests, X-ray, medicines, hospital admission) and non-medical services (i.e. special food/nutrition, transportation, and guardian cost) net of reimbursement. Indirect costs constitutes lost income following the disease episode. In order to estimate the indirect cost, patients were asked to estimate the time lost due to receiving and waiting for care, hospitalization, transportation, lost working days and guardian time (TB). Then the total time lost was multiplied by an hourly wage rate, which was derived from monthly income/consumption by assuming 22 working days a month and 8 hours a day. For children less than 15 years of age, non-medical direct and indirect TB costs was computed for the guardian. Total patient cost is the sum of all direct and indirect costs.

In order to annualize the cost, an average of four HIV outpatient visits and single TB episode outpatient visits was considered. The TB patient cost was extrapolated for the whole duration of TB episode based on an individual data reported retrospectively. All costs were gathered in local currency (Ethiopian Birr) and converted to US dollar ($) with the 2019 exchange rate of $ 1= 29.1468 Ethiopian Birr[33]. For HIV, the cost was first converted to a reference period (2019) using Ethiopia's consumer price index[34]. Due to unavailability of data, we used household income (for HIV) and consumption aggregates (for TB) as a proxy for the household welfare measure and scaled to per adult equivalence (Supplementary appendix 1). In addition, participants were grouped into five-income/consumption quintiles to reflect the socio-economic strata (Supplementary appendix 1).

An incidence of CHE occurs when direct costs (i.e. OOP) exceed a 10% threshold of annual household income/consumption[14, 15, 17]. In addition to the 10% threshold, we carried out

For peer review only - http://bmjopen.bmj.com/site/about/guidelines.xhtml

further analysis at 20% threshold of both OOP and total cost, and at 40% of non-food expenditure to allow comparison [16, 21, 31, 35]. Furthermore, the distribution of financial burden (measured as ratio of direct/total costs to total household expenditure) across income quintile was reported using headcount, overshoot and mean positive overshoot[36] (Supplementary appendix 1).

Data analysis

Data was analysed using STATA version 16 software. The data was summarized using mean with standard deviation (SD) or median with interquartile range (IQR) due to skewed distribution. The cost was disaggregated by outpatient and inpatient care. A concentration index was used to assess health outcome measure inequality across income quintiles[36, 37]. Multivariate logistic regression was conducted to identify determinants of CHE by including significant variables in univariate analysis. A stepwise regression approach was employed to develop the final model and adjusted odds ratios (aOR) with 95% CI was reported. P-value less than 0.05 declares statistical significance for each tests. Multicollinearity was ruled out (variance inflation factor <5). Goodness of fit was checked by Hosmer–Lemeshow test[38].

Ethical consideration

The study was approved by medical and health research ethics in Norway (2018/1647/REK) and Ethiopian public health institute (EPHI-IRB-121-2018). Informed written consent was obtained from TB participants. Oromia and Afar regional health bureau, and respective zonal health offices provided permission to undertake the TB study.

For peer review only - http://bmjopen.bmj.com/site/about/guidelines.xhtml

208 **RESULTS**

209 Out of the total 1,006 HIV and 787 TB participants, 75% of HIV and 50% of TB were females 210 (Table 1). More than two thirds of the study participants were in the age group between 25-44 211 years for HIV and 18-34 years for TB. The median family size was 4 (IQR, HIV: 3–5, IQR, TB: 212 3-6). The mean (SD) household annual income/consumption per adult equivalence was $1,188 213 ($1,288) for HIV and $545 ($462) for TB. Seven percent of TB patients were co-infected with 214 HIV. Almost all (99%) of HIV and 91% of TB/HIV co-infected were receiving ART. About 22% 215 and 6% of HIV and TB patients had both outpatient and inpatient care, respectively. The mean 216 hospital stay was 11 days for HIV and 10 days for TB. The mean (SD) time interval from first 217 health care visit to TB diagnosis (health system delay) was 14 (38) days and 85% of TB were 218 diagnosed in public health facilities.

219 **Table 1:** Socio-demographic and clinical characteristics of HIV and TB study participants 220 (Ethiopia).

**Background characteristics**		**HIV (N=1006)**	**TB (N=787)**
		**N (%)**	**N (%)**
Gender	Female	756 (75)	396 (50)
Age in years (mean, SD)		40 (10)	30 (14)
Age group	<18	-	110 (14)
	18-24	14 (1)	190 (24)
	25-34	268 (27)	229 (29)
	35-44	435 (43)	117 (15)
	45-54	202 (20)	86 (11)
	55-64	58 (6)	32 (4)
	65+	29 (3)	23 (3)
Marital status	Single	38 (4)	309 (39)
	Married/Living together	493 (49)	409 (52)
	Widowed	277 (27)	24 (3)
	Divorced	137 (14)	33 (4)
	Separated	61 (6)	11 (2)
Place of residence	Urban	930 (92)	394 (50)
	Rural	76 (8)	393 (50)
Highest level of education	Illiterate	290 (29)	257 (33)
	Elementary	457 (45)	334 (42)
	Secondary and Higher	259 (26)	195 (25)
Family size	≤ 4	640 (64)	443 (56)
	>4	366 (36)	344 (44)
Annual household	Lowest	200 (20)	159 (20)

For peer review only - http://bmjopen.bmj.com/site/about/guidelines.xhtml

income/consumption	Second	470 (21)	298 (20)
	Middle	797 (18)	443 (20)
	Fourth	1,342 (20)	631 (20)
	Highest	3,084 (21)	1,198 (20)
ART status	On ART	996 (99)	52 (91)
	Not on ART	10 (1)	5 (9)
Past history of illness*	Yes	1,006 (24)	61 (8)
	No	3,165 (76)	726 (92)
Type of visit	Outpatient	790 (79)	739 (94)
	Inpatient	216 (22)	47 (6)
Number of visits per year/TB episode	Outpatient	4,428 (4 visit/patient)	48,720 (70 visit/patient)**
	Inpatient	249 (1 visit/patient)	2,409 (73 visit/patient)**
Type of TB	Pulmonary TB	-	507 (65)
	Extra-pulmonary TB	-	222 (28)
	Drug resistant TB	-	57 (7)

*HIV related comorbidities (HIV), and history of TB (TB)

** The number of daily visits per patient reaches 125 for outpatient and 135 for inpatient drug resistant TB cases.

Patient cost of HIV and TB care

The total mean (SD) patient cost for HIV care was $78 ($170) per year and $115 ($118) for the entire duration of a TB episode (Table 2). The mean (SD) direct cost was $54 ($144) for HIV and $53 ($59) for TB, which constitutes 69% and 46% of the total cost, respectively. Medical costs contributed to 68% and 38% of the direct costs for HIV and TB, respectively. Diagnostics and medicine account for 39% of the total HIV cost. The mean (SD) indirect cost was $24 ($66) for HIV per year, and $63 ($83) per TB episode. The productivity loss related to TB follow-up visits accounts for 36% of the total cost.

The mean (SD) total cost was $63 ($165) for annual outpatient HIV care visit and $110 ($114) for the whole duration of outpatient TB care (Table 2). Similarly, the mean (SD) total cost for each hospitalization was $96 ($139) for HIV and $105 ($78) for TB. For patients having both outpatient and inpatient visits, the mean cost reaches $133 ($178) ($p<0.001$) for HIV and $ 217 ($157) ($p<0.001$) for TB.

For TB, the patient costs incurred prior to initiation of treatment is equal to the cost from initiation to completion of TB treatment (paired t-test>0.05). The mean (SD) total cost for those with

For peer review only - http://bmjopen.bmj.com/site/about/guidelines.xhtml

TB/HIV co-infection reached $188 ($33). Similarly, the total cost of care significantly varies by type of TB, was $104 ($107) for pulmonary, $140 ($138) for extra-pulmonary and $446 ($732) for DR-TB (Kruskal Wallis test 41.1, P <0.001) (Supplementary appendix 1: Table A1).

Coping costs

HIV and TB care results in adverse financial consequences for households. Nearly 24% of HIV and 68% of TB study participants have adopted coping mechanisms. Nine percent of HIV and 4% of TB patients have borrowed money; while, 2% of HIV and 19% of TB patients sold their household assets. Furthermore, 12% of HIV patient relied on family assistance and 16% of TB patient used their savings to cope with the costs. Only 2% of HIV and 6% of TB participants are covered by health insurance.

Table 2: Distribution of household direct, indirect and total cost of HIV and TB care across main cost category in Ethiopia (expressed in $).

Cost category ($)		HIV (N=1,006)			TB (N=729)		
		Outpatient (Per year)	Inpatient (Per single visit)	Total	Outpatient (Per TB episode)	Inpatient (Per single visit)	Total
**I. Direct medical cost**							
Consultation fee	Mean (SD)	6 (15)	1 (5)	6 (15)	2 (5)	11 (16)	3 (7)
	Median (IQR)	0 (0-6)	0 (0-1)	0 (0-5)	1 (0-2)	7 (1-14)	1 (0-2)
Investigation cost	Mean (SD)	14 (45)	9 (19)	15 (47)	8 (13)	14 (16)	9 (15)
	Median (IQR)	0 (0-11)	0 (0-9)	1 (0-12)	4 (0-12)	7 (3-18)	5 (0-13)
Drug cost[1]	Mean (SD)	12 (40)	22 (63)	16 (48)	7 (14)	16 (21)	8 (16)
	Median (IQR)	0 (0-10)	4 (0-19)	1 (0-13)	1 (0-10)	8 (3-20)	2 (0-10)
**Subtotal**	Mean (SD)	32 (88)	32 (77)	37 (98)	17 (24)	40 (37)	20 (30)
	Median (IQR)	4 (0-34)	8 (0-32)	6 (0-38)	10 (1-22)	30 (16-45)	11 (1-25)
**II. Direct non-medical cost**							
Transportation fee	Mean (SD)	11 (55)	19 (34)	13 (53)	8 (10)	7 (7)	8 (10)
	Median (IQR)	2 (0-8)	6 (0-20)	3 (0-9)	5 (2-11)	7 (3-9)	5 (2-11)
Food/accommodation	Mean (SD)	3 (50)	10 (30)	5 (48)	23 (29)	28 (34)	25 (31)
	Median (IQR)	0	0 (0-5)	0	15 (7-27)	16 (7-38)	16 (7-29)
**Subtotal**	Mean (SD)	14 (80)	29 (49)	17 (76)	31 (34)	35 (36)	33 (37)
	Median (IQR)	2 (0-8)	10 (0-38)	3 (0-10)	21 (11-38)	20 (9-44)	21 (12-40)
**Total direct cost**	Mean (SD)	46 (142)	60 (113)	54 (144)	48 (50)	75 (68)	53 (59)
	Median (IQR)	12 (1-45)	22 (4-74)	15 (2-57)	35 (17-61)	54 (26-94)	36 (18-64)
**III. Indirect cost**							
Foregone income before treatment[2]	Mean (SD)	0	0	0	11 (28)	0	11 (26)
	Median (IQR)	0	0	0	0 (0-5)	0	0 (0-3)
Foregone income during treatment	Mean (SD)	11 (44)	22 (41)	16 (50)	11 (26)	12 (31)	10 (26)
	Median (IQR)	0	8 (3-19)	0 (0-8)	0 (0-4)	0	0 (0-3)

For peer review only - http://bmjopen.bmj.com/site/about/guidelines.xhtml

Time loss related cost	Mean (SD)	6 (19)	14 (52)	8 (30)	42 (59)	18 (24)	42 (58)
	Median (IQR)	2 (1-4)	2 (1-7)	2 (1-5)	27 (14-49)	8 (5-22)	27 (14-49)
**Subtotal**	Mean (SD)	17 (54)	35 (75)	24 (66)	62 (84)	30 (37)	63 (83)
	Median (IQR)	3 (1-8)	13 (5-32)	4 (2-17)	36 (17-77)	9 (5-39)	36 (17-78)
**IV. Total cost**	Mean (SD)	63 (165)	96 (139)	78 (170)	110 (114)	105 (78)	115 (118)
	Median (IQR)	20 (5-63)	52 (25-109)	27 (7-80)	79 (46-140)	87(39-158)	81 (47-150)

251 [1]Drug other than anti-retroviral and anti TB drugs
252 [2]Not captured in HIV survey

253 As shown in figure 1, the spikes in the pen's parade graph revealed that the health care costs of HIV and TB cause a large decrease in annual income/consumption for many of the households. The consumption drop is more pronounced for TB than the income drop for HIV (Figure 1).

256 [Figure 1 here]

257 As shown in Table 3, there are inequalities in OOP costs among HIV and TB participants. The total cost rises steadily across the income quintiles and is concentrated among the richer quintiles (i.e. the richer the quintile, the higher the cost). From the lowest to the highest income/consumption quintile, the mean (median) total cost of HIV increases from \$53 (\$20) to \$133 (\$60), with significant difference among income quintiles (Kruskal Wallis test 44.7, P < 0.001), and for TB the cost increases from \$50 (\$36) to \$202 (\$148) (Kruskal Wallis test 206.5, P < 0.001). In general, the median cost (both direct and indirect) seems to be a bit higher for TB patients as compared to those with HIV.

265 **Table 3.** Mean (median) HIV and TB patient costs per year across income quintiles in Ethiopia (expressed in \$).

Disease Category	Income quintiles	Cost type					
		Direct		Indirect		Total cost	
		Mean (SD)	Median (IQR)	Mean (SD)	Median (IQR)	Mean (SD)	Median (IQR)
**HIV**	Poorest	45 (92)	14 (2-48)	8 (15)	2 (1-7)	53 (97)	20 (3-61)
	Poor	55 (170)	13 (0-52)	10 (19)	3 (1-9)	65 (173)	22 (4-65)
	Middle	50 (118)	19 (3-49)	16 (30)	3 (2-16)	66 (136)	32 (7-65)
	Rich	47 (89)	15 (3-57)	25 (51)	4 (3-20)	71 (111)	27 (7-81)
	Richest	73 (206)	19 (3-79)	60 (124)	14 (5-59)	133 (262)	60 (12-132)
**P-value***		0.358		<0.001		<0.001	
**TB**	Poorest	31 (35)	23 (7-41)	19 (20)	11 (6-24)	50 (44)	36 (20-66)
	Poor	44 (48)	27 (15-54)	36 (32)	27 (13-45)	79 (65)	60 (38-96)

For peer review only - http://bmjopen.bmj.com/site/about/guidelines.xhtml

	Middle	49 (53)	36 (17-61)	57 (46)	40 (22-82)	106 (79)	82 (57-137)
	Rich	61 (48)	49 (28-84)	79 (63)	58 (27-117)	140 (96)	118 (68-181)
	Richest	78 (86)	54 (34-99)	124 (145)	70 (40-163)	202 (189)	148 (88-260)
**P-value***		< 0.001		< 0.001		< 0.001	

*kruskal wallis test

Incidence and intensity of CHE

At the 10% threshold, the overall CHE incidence of HIV was 20% (197 households); with 43% of the poorest and 4% of the richest household experiencing CHE (Chi-square for trend -10.58, P <0.001) (Figure 2a, Supplementary appendix 1: Table A2). The incidence is 33% for individuals with inpatient HIV care. The corresponding level of TB was 40% (291 households); with 58% and 20% of the poorest and richest income-quintile experienced CHE, respectively (Chi-square for trend -6.79, P <0.001) (Table 4). The incidence was much higher for those with TB/HIV co-infection (48%), DR-TB (62%) and was almost universal (94%) for hospitalized TB patient. At the 20% threshold of total expenses, 48% (353 households) of TB households experienced catastrophic total costs (Fig 2b, Supplementary appendix 1: Table A2).

In our study, for example, the mean overshoot of TB related CHE was 6.3% (range: 1.9%-15.3%). On average, households spent 6.3% beyond the 10% threshold for TB care. However, the average positive overshoot among households that experienced CHE was 15.8% (range: 9.2% - 26.6%). Thus, on average, households that experienced CHE spent 25.8% (10% threshold + mean positive overshoot) of their total annual consumption for TB care (Figure 2, Supplementary appendix 1: Table A2).

[Figure 2 here]

Inequality in financial risk

In addition, as shown in figure 3, inequality in financial risk across income/consumption groups exists. The concentration curves for HIV and TB care costs lies below the 45° line of equality, which shows a greater concentration of the costs among the rich. However, the financial burden

For peer review only

For peer review only - http://bmjopen.bmj.com/site/about/guidelines.xhtml

is higher among the poor – the concentration curves for HIV and TB care expenditure in relation to income/consumption lies above the 45° line of equality.

[Figure 3 here]

Determinants of CHE

In the multivariate analysis (Table 4), three variables were independently associated with HIV related CHE: hospitalized patients (aOR: 3.3, 95% CI: 2.2-4.9), being poorest (aOR: 18.4, 95% CI: 8.9-37.7) and poor (aOR: 9.4, 95% CI: 4.5-19.5) were associated with catastrophic HIV care expenditures. Moreover, every additional visit for HIV care increases the odds of CHE by 7% (aOR: 1.07, 95% CI: 1.003-1.1).

Seven variables were significantly associated with TB related CHE (Table 4): private facility diagnosis (aOR: 2.6, 95%CI: 1.52-4.33); extra-pulmonary TB (aOR: 2.6, 95%CI: 1.77-3.95); hospitalized patients (aOR: 30.6, 95% CI: 4.77-199.83); being poorest (aOR: 14.6, 95% CI: 7.49-28.26); and TB/HIV co-infection (aOR: 3.2, 95% CI: 1.63-6.15) were very likely to have TB related CHE as compared to their counterparts after adjusting for other variables. Every additional visit for TB diagnosis increases the odds of experiencing CHE by 2.4 times (aOR: 2.4, 95% CI: 1.92-3.05). Households with a health insurance scheme have protection from CHE (aOR 2.7; 95%CI 1.06–6.73).

Table 4. Multivariate logistic regression model of determinants of CHE for TB and HIV care at a 10% threshold of household income/consumption (Ethiopia).

**TB**<br>**Variable**	**aOR (95% CI)**	**P-value**	**HIV**<br>**Variable**	**aOR (95% CI)**	**P-value**
Frequency of visits¥	2.4 (1.9-3.1)	<0.001	Frequency of visits Per year¥	1.07 (1.003-1.1)	0.040
Hospitalization			Hospitalization		
No	Ref.		No	Ref.	
Yes	30.6 (4.8-199.8)	0.001	Yes	3.3 (2.2-4.9)	<0.001
Income quintiles			Income quintiles		
Richest	Ref.		Richest	Ref.	
Rich£	4.1 (2.1-7.8)	<0.001	Rich	2.5 (1.1-5.8)	0.025
Middle	4.9 (2.5-9.4)	<0.001	Middle	4.5 (2.1-9.8)	<0.001

For peer review only

For peer review only - http://bmjopen.bmj.com/site/about/guidelines.xhtml

Poor	7.0 (3.6-13.7)	<0.001	Poor	9.4 (4.5-19.5)	<0.001
Poorest	14.6 (7.5-28.3)	<0.001	Poorest	18.4 (8.9-37.7)	<0.001
Place of diagnosis					
Government	Ref.				
Private	2.6 (1.5-4.3)	<0.001			
TB/HIV co-infection					
No	Ref.				
Yes	3.2 (1.6-6.2)	0.001			
Insurance (i.e. CBHI)					
Yes	Ref.				
No	2.7 (1.1-6.7)	0.038			
Type of TB					
Bacteriologically confirmed TB	Ref.				
Clinically diagnosed TB	1.6 (1.0-2.8)	0.075			
Extra pulmonary TB[£]	2.6 (1.8-4.0)	<0.001			

[¥] Variable treated as continuous

[£]Overall test is significant

CBHI: Community based health insurance

For peer review only

DISCUSSION

In this study, we tried to estimate the OOP, total cost, incidence and determinants of CHE among individuals seeking HIV and TB care. This could provide valuable insight into the level of FRP that a health system offers to its population.

HIV care costs and CHE

In Ethiopia, where ART is given free-of-charge, PLHIV had to pay a total of $78 per year for HIV care ($19.5 per visit). The total HIV patient cost was equivalent to 7% of their annual income. As HIV requires lifelong care, such cost have devastating impact on affected households. Our estimate was lower than the level and rate found in Cameron 17%[39], Ethiopia 21%[23], Nepal 28.5%[40] and South Africa (30%)[41]. These variations might arise from different study settings[39, 40]; high expenses of additional food and time loss[41]. Similarly, more centralized HIV service delivery, delays in seeking care, and long distance travel to access services may explain the difference with the previous study from Ethiopia. Additionally, the annual income in this study was twice the latter study[23]. However, a study on outpatient HIV care in Nigeria reported one-fourth of the cost in this study ($21.76)[42].

In our study, the average direct OOP expenditure for HIV care was $54 (i.e. 69% of the total cost) and comparable with previous studies from Lao, Ethiopia and Nepal[19, 23, 40]. Diagnostics, medicines and transportation costs constitute the largest share, and may pause a serious challenge to the success of the HIV program. This calls for public financing policies (i.e. free of charge diagnosis and treatment of HIV related comorbidities for vulnerable groups) in the next steps of UHC expansion. The productivity loss found here was one half of the previous studies in Ethiopia and Nepal,[23, 40] but one fourth of that of Lao study[19]. The lower cost in our study may be due to the wider scale up of ART in recent years, which minimizes costs of pre-ART treatment and advanced HIV infection management. The total costs of HIV care

For peer review only - http://bmjopen.bmj.com/site/about/guidelines.xhtml

increases as income rises. The equity ratios (Q1:Q5, 0.39) showed that higher expenditure among the richest income quintile, which is consistent with a study from southeast Nigeria[43].

In our study, about a fifth (20%) of patients seeking HIV care experienced CHE. A study from India also depicts similar findings[44]. However, the rate is lower than a study from Cameroon[39]. Furthermore, the incidence of HIV related CHE remained relatively high; in particular, where poorest households suffer more. Similar socio-economic inequality was observed in previous studies,[42, 43] highlighting the importance of rendering equitable access to all in need of HIV care, particularly for the poor[39]. Consistent with previous studies, being poor is associated with higher CHE[19, 45]. In addition, the poor were pushed further beyond the CHE threshold than the better off. This is of great concern, as health shocks are slightly managed by the poor through reduction of basic requirements to compensate for HIV care. Similar to previous studies, being hospitalized was a stronger determinant of CHE[19, 42].

TB care costs and CHE

In our study, patients with TB incurred a total cost of $115 per episode and represents 21% of the annual household income, comparable to a study in South Africa (22%)[46]. However, the total cost was less than the figure reported by previous studies in Ethiopia[22, 29], but twice higher than the finding from southern Ethiopia[47]. The variation in cost from previous studies arises from high expenditure on nutritional supplements ($72 vs $25)[29], lower level of seeking diagnostic care from public health facilities (64% vs 85%), high rates of clinically diagnosed cases (49% vs 24%), diagnostic delays result in longer losses of working days [22], only direct cost captured[47]. The average patient costs of DR-TB care were four times higher than drug susceptible TB care. However, the DR-TB cost was three times lower than findings from another Ethiopian study[48]. Still the devastating nature of DR-TB may put them at special risk of CHE. The lower costs is mainly due to decentralization of DR-TB services in recent years and the

For peer review only - http://bmjopen.bmj.com/site/about/guidelines.xhtml

introduction of shorter DR-TB treatment regimen (i.e. 71% the study participants took 9-12 month regimen).

In this study, one-half of the mean TB cost was incurred prior to initiation of treatment, which was consistent with many previous studies[11, 21, 22, 49, 50]. This is mostly due to payment while demanding for proper diagnosis of TB[21]. In addition to the financial burden of high pre-treatment costs, it will be a barrier to complete the diagnostic process, and to timely access treatment and care. These emphasize the need for early case finding with rapid and point of care TB diagnostics, involvement of private care providers, and instituting effective referral and linkage[47].

The direct patient costs incurred constitutes 46% of the total, comparable with findings from elsewhere (36%) [51] and systematic review result (40%)[11]. However, the proportion was higher than the finding from southwestern Ethiopia (29%) and lower than the report from central Ethiopia (71%)[22, 29]. Consistent with previous studies, non-medical and indirect costs represent a large share of the TB cost, while medical cost represent less than 20%[11, 22, 41]. Therefore, ensuring the expansion of TB service package through the effective integration of a health insurance scheme and decentralization of services can reduce the direct costs.

A higher percentage of households incur CHE for TB care (40%), which was comparable with studies from Fiji (40%), Ghana (47.6%), China (53%), Philippines (35%) and lower than reported rates from Ethiopia (63%), Nigeria (65%) and Benin (72%)[29, 49, 50, 52-54]. However, CHE for TB care was higher in this study compared to findings in studies from India (21%) and Malaysia (6%)[51, 55]. This variation might arise from cost estimation method, study setting, health system and socioeconomic differences. The TB related CHE was higher than the reported rate for HIV. One important reason for this is the difference in the duration of treatment and intervention characteristics between the two diseases. TB patients experience a very onerous set of direct and indirect costs during diagnostic and intensive phase of directly observed short course

For peer review only - http://bmjopen.bmj.com/site/about/guidelines.xhtml

therapy (i.e. more intense for retreatment, extra-pulmonary and DR-TB cases). After treatment completion and possible sputum conversion, TB patients are less likely to face additional costs. Even though the annual cost of HIV care is lower, PLHIV faces these costs over its lifetime because HIV infection is a chronic disease that needs lifelong treatment. The comparison of incidence and intensity of CHE for TB and HIV is also complicated by the use of income for HIV and consumption for TB-related computations. In developing countries, income is poor self-reported estimator of welfare due to more common informal employment, seasonal agricultural activities and widespread reluctance to disclose income[15]. Therefore, income could understate the welfare of the household, whereas using consumption may overstate the condition of the household because of the use of dissaving/borrowing to smooth consumption over time[56].

The mean overshoot for TB was 6.3% and the MPO was 15.3%, both were similar with finding from Benin (7.8% and, 14.8%) and Nigeria (6.0% and, 9.3%)[50, 52]. TB, inequitably, imposes a greater burden of CHE on the poor households. Even though poor households tend to spend less, a higher share of their income is spent on seeking TB care[12, 29, 46, 50, 52]. In addition, the excess CHE beyond the threshold was inequitably high among the poor. This finding is also in line with other studies[29, 50, 52]. Similar to other studies, hospitalization, income status, and TB/HIV coinfection were among the key determinants of TB related CHE[28, 49, 50, 52, 55]. In addition, even if the health insurance coverage (i.e.CBHI) was low and is limited to medical costs, we found protective effect of the scheme against CHE[57]. However, health insurance per se does not alleviate the major TB costs.

Study limitation and strengths

This study has some limitations. First, the cost measurements relied on patient's ability to remember, which increases risk of recall bias. However, we reduced the recall bias by interviewing participants who sought HIV and TB care within the past one month, when patients have a better recollection of their pathway to care[28, 31]. Second, HIV costs may be

For peer review only - http://bmjopen.bmj.com/site/about/guidelines.xhtml

overestimated when aggregated over one-year period. Third, our findings might not be representative of all PLHIV and TB patients in Ethiopia, as the sampling for HIV mainly consisted of urban, and excludes person not member of PLHIV association and the TB study is limited to specific regions of the country. Moreover, undetected HIV and TB cases not seeking care were not addressed. Despite these limitations, we used a standard tool and method to conduct the study. We believe that our study will be of high value to inform policy, at national and sub-national levels, related to FRP of both diseases, which are central in achieving UHC.

To this end, despite OOP exemption of HIV and TB services in Ethiopia, we found that there is a large gap between the actual level of financial protection provided and the ideal goal. Our findings highlight several policy implications. First, the introduction of more patient centred model of care with effective diagnostics is required in order to optimize HIV and TB service delivery. Second, strategies to ensure social and FRP for HIV and TB affected households are necessary. This requires effective integration of HIV and TB services with health insurance, provision of differentiated care and community-based treatment for stable patients, and enabling access to social protection schemes. Furthermore, national HIV and TB programs should adopt public finance policies where equitable and quality services are provided free of OOP for all in need.

CONCLUSION

HIV and TB affected individuals and their households in Ethiopia face substantial costs in seeking care despite "free medical services". The incidence of CHE related to HIV and TB care were high in all income quintiles, though more so in the poorest households. Policy makers should introduce patient centred care; expand FRP and social protection measures to minimize the high patient cost of HIV and TB care, particularly among vulnerable populations.

For peer review only - http://bmjopen.bmj.com/site/about/guidelines.xhtml

Supplementary file: Supplementary appendix 1: Consumption construction, and measurement of CHE, findings

Availability of data: The data supporting the conclusion of this study can be available upon reasonable request from the corresponding author.

Competing Interests: None declared

Funding: Bill & Melinda Gates Foundation grant number OPP1162384 supported this study.

Contributors: LFA, KAJ, MTT: conception and design of the study. LFA: acquisition of data, data analysis, and drafted the paper. LFA, AJ, KAJ, MTT, EKN: critically reviewed the paper and gave approval for the final version to be published.

Acknowledgments: The authors would like to thank all HIV and TB patients and supervisors who participated in this study. We thank all stakeholders, the Ministry of Health, Ethiopia, Ethiopian public health institute, Oromia and Afar regional health Bureau with their respective districts for making this research possible.

Figure 1. Pen's parade of household annual income/consumption gross and net of payments for HIV (a) and TB care (b) (Ethiopia).

Figure 2. Interval plot (with 95% confidence interval for the mean) of incidence and intensity of HIV and TB-related catastrophic health expenditures (CHE) across income quintiles, using 10% threshold (Ethiopia).

Figure 3. Concentrations curves and index (I) for direct, indirect, total cost and share of OOP to income/consumption for (a) HIV and (b) TB services in Ethiopia.

For peer review only - http://bmjopen.bmj.com/site/about/guidelines.xhtml

REFERENCES

1. Vitoria M, Granich R, Gilks CF, et al. The global fight against HIV/AIDS, tuberculosis, and malaria: current status and future perspectives. *American journal of clinical pathology*. 2009;131(6):844-8 DOI: 10.1309/ajcp5xhdb1pnaeyt.
2. Danforth K, Granich R, Wiedeman D, et al. Global Mortality and Morbidity of HIV/AIDS. 2017:29-44.
3. Bloom BR, Atun R, Cohen T, et al. Tuberculosis. 2017:233-313.
4. World Health Organization. Global tuberculosis report 2019. Geneva: World Health Organization; 2019.
5. World Health Organization. WHO fact sheet on HIV/AIDs. 25 July 2019. Available from: https://www.who.int/news-room/fact-sheets/detail/hiv-aids. Accessed 1 Sep 2019.
6. UNAIDS. UNAIDS data 2019. Available from https://www.unaids.org/en/resources/documents/2019/2019-UNAIDS-data. Accessed 24 Nov 2019.
7. WHO. IMPLEMENTING THE END TB STRATEGY: THE ESSENTIALS Geneva: World Health Organization; 2015.
8. Babigumira JB, Stergachis A, Veenstra DL, et al. Potential cost-effectiveness of universal access to modern contraceptives in Uganda. *PLoS One*. 2012;7(2):e30735 DOI: 10.1371/journal.pone.0030735.
9. The Federal Democratic Republic of Ethiopia Ministry of Health. Health Sector Transformation Plan 2015/16 - 2019/2020(2008-2012 EFY). Addis Ababa, Ethiopia.2015.
10. Federal Democratic Republic of Ethiopia Ministry of Health. September 2019.Ethiopia Health Accounts, 2016/17. Addis Ababa, Ethiopia.
11. Tanimura T, Jaramillo E, Weil D, et al. Financial burden for tuberculosis patients in low- and middle-income countries: a systematic review. *The European respiratory journal*. 2014;43(6):1763-75 DOI: 10.1183/09031936.00193413.
12. Russell S. The economic burden of illness for households in developing countries: a review of studies focusing on malaria, tuberculosis, and human immunodeficiency virus/acquired immunodeficiency syndrome. *The American journal of tropical medicine and hygiene*. 2004;71(2 Suppl):147-55.
13. Laurence YV, Griffiths UK, Vassall A. Costs to Health Services and the Patient of Treating Tuberculosis: A Systematic Literature Review. *PharmacoEconomics*. 2015;33(9):939-55 DOI: 10.1007/s40273-015-0279-6.
14. Wagstaff A, van Doorslaer E. Catastrophe and impoverishment in paying for health care: with applications to Vietnam 1993-1998. *Health economics*. 2003;12(11):921-34 DOI: 10.1002/hec.776.
15. O'Donnell O, Van Doorslaer E, Wagstaff A, et al. Analyzing Health Equity Using Household Survey Data A Guide to Techniques and Their Implementation. 2012.
16. Xu K, Evans DB, Kawabata K, et al. Household catastrophic health expenditure: a multicountry analysis. *Lancet (London, England)*. 2003;362(9378):111-7 DOI: 10.1016/s0140-6736(03)13861-5.
17. Kawabata K, Xu K, Carrin G. Preventing impoverishment through protection against catastrophic health expenditure. *Bulletin of the World Health Organization*. 2002;80(8):612.
18. Lopera MM, Einarson TR, Ivan Bula J. Out-of-pocket expenditures and coping strategies for people living with HIV: Bogota, Colombia, 2009. *AIDS care*. 2011;23(12):1602-8 DOI: 10.1080/09540121.2011.579938.
19. Barennes H, Frichittavong A, Gripenberg M, et al. Evidence of High Out of Pocket Spending for HIV Care Leading to Catastrophic Expenditure for Affected Patients in Lao People's Democratic Republic. *PLOS ONE*. 2015;10(9):e0136664 DOI: 10.1371/journal.pone.0136664.

For peer review only - http://bmjopen.bmj.com/site/about/guidelines.xhtml

20. Long Q, Smith H, Zhang T, et al. Patient medical costs for tuberculosis treatment and impact on adherence in China: a systematic review. *BMC Public Health*. 2011;11(1):393 DOI: 10.1186/1471-2458-11-393.
21. Wingfield T, Boccia D, Tovar M, et al. Defining catastrophic costs and comparing their importance for adverse tuberculosis outcome with multi-drug resistance: a prospective cohort study, Peru. *PLoS medicine*. 2014;11(7):e1001675 DOI: 10.1371/journal.pmed.1001675.
22. Asres A, Jerene D, Deressa W. Pre- and post-diagnosis costs of tuberculosis to patients on Directly Observed Treatment Short course in districts of southwestern Ethiopia: a longitudinal study. *Journal of health, population, and nutrition*. 2018;37(1):15 DOI: 10.1186/s41043-018-0146-0.
23. Vassall A, Seme A, Compernolle P, et al. Patient costs of accessing collaborative tuberculosis and human immunodeficiency virus interventions in Ethiopia. *The international journal of tuberculosis and lung disease : the official journal of the International Union against Tuberculosis and Lung Disease*. 2010;14(5):604-10.
24. Mauch V, Bonsu F, Gyapong M, et al. Free tuberculosis diagnosis and treatment are not enough: patient cost evidence from three continents. *The international journal of tuberculosis and lung disease : the official journal of the International Union against Tuberculosis and Lung Disease*. 2013;17(3):381-7 DOI: 10.5588/ijtld.12.0368.
25. Bhojani U, Thriveni B, Devadasan R, et al. Out-of-pocket healthcare payments on chronic conditions impoverish urban poor in Bangalore, India. *BMC Public Health*. 2012;12:990 DOI: 10.1186/1471-2458-12-990.
26. Ministry of Health, Ethiopia. Annual health sector performance report 2011 EFY (2018/19). Addis Ababa, Ethiopia.
27. World Health Organization. Health Financing: Monitoring Sustainable Development Goals—Indicator 3.8.2; World Health Organization: Geneva, Switzerland, 2019. Available from: https://www.who.int/health financing/topics/financial-protection/monitoring-sdg/en/. Accessed 5 Sep 2019.
28. Ukwaja KN, Alobu I, lgwenyi C, et al. The High Cost of Free Tuberculosis Services: Patient and Household Costs Associated with Tuberculosis Care in Ebonyi State, Nigeria. *PLOS ONE*. 2013;8(8):e73134 DOI: 10.1371/journal.pone.0073134.
29. Getahun B, Wubie M, Dejenu G, et al. Tuberculosis care strategies and their economic consequences for patients: the missing link to end tuberculosis. *Infectious Diseases of Poverty*. 2016;5(1):93 DOI: 10.1186/s40249-016-0187-9.
30. Ministry of Health, Ethiopia. Health Management Information System (HMIS) annual TB and Leprosy report 2016. Addis Ababa, Ethiopia.
31. WHO. WHO Global TB Programme. Protocol for survey to determine direct and indirect costs due to TB and to estimate proportion of TB-affected households experiencing catastrophic costs. November 2015.
32. Federal Democratic Republic of Ethiopia Ministry of Health. Health Service Utilization and Expenditure Survey among People Living with HIV (PLHIV), 2015/16, Addis Ababa, Ethiopia. August 2017.
33. National Bank of Ethiopia. Inter-bank daily foreign exchange rate in (USD)—archive. 2019. Available from: https://www.nbe.gov.et/market/dailyexchange.html. Accessed 29 Oct 2019.
34. International Monetary Fund.World Economic Outlook Database October 2015. Available from: https://www.imf.org/external/pubs/ft/weo/2019/02/weodata/index.aspx . Accessed 17 May 2019.
35. WHO Global TB Programme. Protocol for survey to determine direct and indirect costs due to TB and to estimate proportion of TB-affected households experiencing catastrophic costs. November 2015.

For peer review only

For peer review only - http://bmjopen.bmj.com/site/about/guidelines.xhtml

36. O'Donnell OvD, Eddy Wagstaff, Adam Lindelow, Magnus. Analyzing Health Equity Using Household Survey Data.
37. O'Donnell O, O'Neill S, Van Ourti T, et al. conindex: Estimation of concentration indices. *The Stata journal*. 2016;16(1):112-38.
38. L. Gortmaker S, Hosmer D, Lemeshow S. Applied Logistic Regression. *Contemporary Sociology*. 1994;23:159 DOI: 10.2307/2074954.
39. Boyer S, Abu-Zaineh M, Blanche J, et al. Does HIV services decentralization protect against the risk of catastrophic health expenditures?: some lessons from Cameroon. *Health services research*. 2011;46(6pt2):2029-56 DOI: 10.1111/j.1475-6773.2011.01312.x.
40. Poudel AN, Newlands D, Simkhada P. The economic burden of HIV/AIDS on individuals and households in Nepal: a quantitative study. *BMC health services research*. 2017;17(1):76 DOI: 10.1186/s12913-017-1976-y.
41. Mudzengi D, Sweeney S, Hippner P, et al. The patient costs of care for those with TB and HIV: a cross-sectional study from South Africa. *Health policy and planning*. 2017;32(suppl_4):iv48-iv56 DOI: 10.1093/heapol/czw183.
42. Etiaba E, Onwujekwe O, Torpey K, et al. What Is the Economic Burden of Subsidized HIV/AIDS Treatment Services on Patients in Nigeria and Is This Burden Catastrophic to Households? *PLoS One*. 2016;11(12):e0167117 DOI: 10.1371/journal.pone.0167117.
43. Onwujekwe O, Dike N, Chukwuka C, et al. Examining catastrophic costs and benefit incidence of subsidized antiretroviral treatment (ART) programme in south-east Nigeria. *Health policy (Amsterdam, Netherlands)*. 2009;90(2-3):223-9 DOI: 10.1016/j.healthpol.2008.10.006.
44. Shukla M, Agarwal M, Singh J, et al. Catastrophic health expenditure amongst people living with HIV/AIDs availing antiretroviral treatment services at two tertiary care health facilities in district of northern India. *National Journal of Community Medicine*. 2015;6:323-8.
45. Mnzava T, Mmari E, Berruti A. Drivers of Patient Costs in Accessing HIV/AIDS Services in Tanzania. *Journal of the International Association of Providers of AIDS Care*. 2018;17:2325958218774775 DOI: 10.1177/2325958218774775.
46. Foster N, Vassall A, Cleary S, et al. The economic burden of TB diagnosis and treatment in South Africa. *Social Science & Medicine*. 2015;12 DOI: 10.1016/j.socscimed.2015.01.046.
47. Datiko DG, Lindtjørn B. Cost and Cost-Effectiveness of Treating Smear-Positive Tuberculosis by Health Extension Workers in Ethiopia: An Ancillary Cost-Effectiveness Analysis of Community Randomized Trial. *PLOS ONE*. 2010;5(2):e9158 DOI: 10.1371/journal.pone.0009158.
48. Berhanu RH, Schnippel K, Kularatne R, et al. Can patients afford the cost of treatment for multidrug-resistant tuberculosis in Ethiopia? *The international journal of tuberculosis and lung disease : the official journal of the International Union against Tuberculosis and Lung Disease*. 2018;22(8):358-62 DOI: 10.5588/ijtld.17.0837.
49. Duan W, Zhang W, Wu C, et al. Extent and determinants of catastrophic health expenditure for tuberculosis care in Chongqing municipality, China: a cross-sectional study. *BMJ Open*. 2019;9(4):e026638 DOI: 10.1136/bmjopen-2018-026638.
50. Ukwaja KN, Alobu I, Abimbola S, et al. Household catastrophic payments for tuberculosis care in Nigeria: incidence, determinants, and policy implications for universal health coverage. *Infect Dis Poverty*. 2013;2(1):21 DOI: 10.1186/2049-9957-2-21.
51. Atif M, Sulaiman SA, Shafie AA, et al. Resource utilization pattern and cost of tuberculosis treatment from the provider and patient perspectives in the state of Penang, Malaysia. *BMC health services research*. 2014;14:353 DOI: 10.1186/1472-6963-14-353.
52. Laokri S, Dramaix-Wilmet M, Kassa F, et al. Assessing the economic burden of illness for tuberculosis patients in Benin: determinants and consequences of catastrophic health

For peer review only

For peer review only - http://bmjopen.bmj.com/site/about/guidelines.xhtml

expenditures and inequities. *Tropical medicine & international health : TM & IH*. 2014;19(10):1249-58 DOI: 10.1111/tmi.12365.

53. Viney K, Islam T, Hoa NB, et al. The Financial Burden of Tuberculosis for Patients in the Western-Pacific Region. *Tropical medicine and infectious disease*. 2019;4(2) DOI: 10.3390/tropicalmed4020094.
54. Pedrazzoli D, Siroka A, Boccia D, et al. How affordable is TB care? Findings from a nationwide TB patient cost survey in Ghana. *Tropical medicine & international health : TM & IH*. 2018;23(8):870-8 DOI: 10.1111/tmi.13085.
55. Prasanna T, Jeyashree K, Chinnakali P, et al. Catastrophic costs of tuberculosis care: a mixed methods study from Puducherry, India. *Global health action*. 2018;11(1):1477493 DOI: 10.1080/16549716.2018.1477493.
56. Tracking universal health coverage: 2017 global monitoring report. World Health Organization and International Bank for Reconstruction and Development / The World Bank; 2017. Licence: CC BY-NC-SA 3.0 IGO.
57. Zhou C, Long Q, Chen J, et al. The effect of NCMS on catastrophic health expenditure and impoverishment from tuberculosis care in China. *International Journal for Equity in Health*. 2016;15(1):172 DOI: 10.1186/s12939-016-0463-0.

For peer review only

For peer review only - http://bmjopen.bmj.com/site/about/guidelines.xhtml

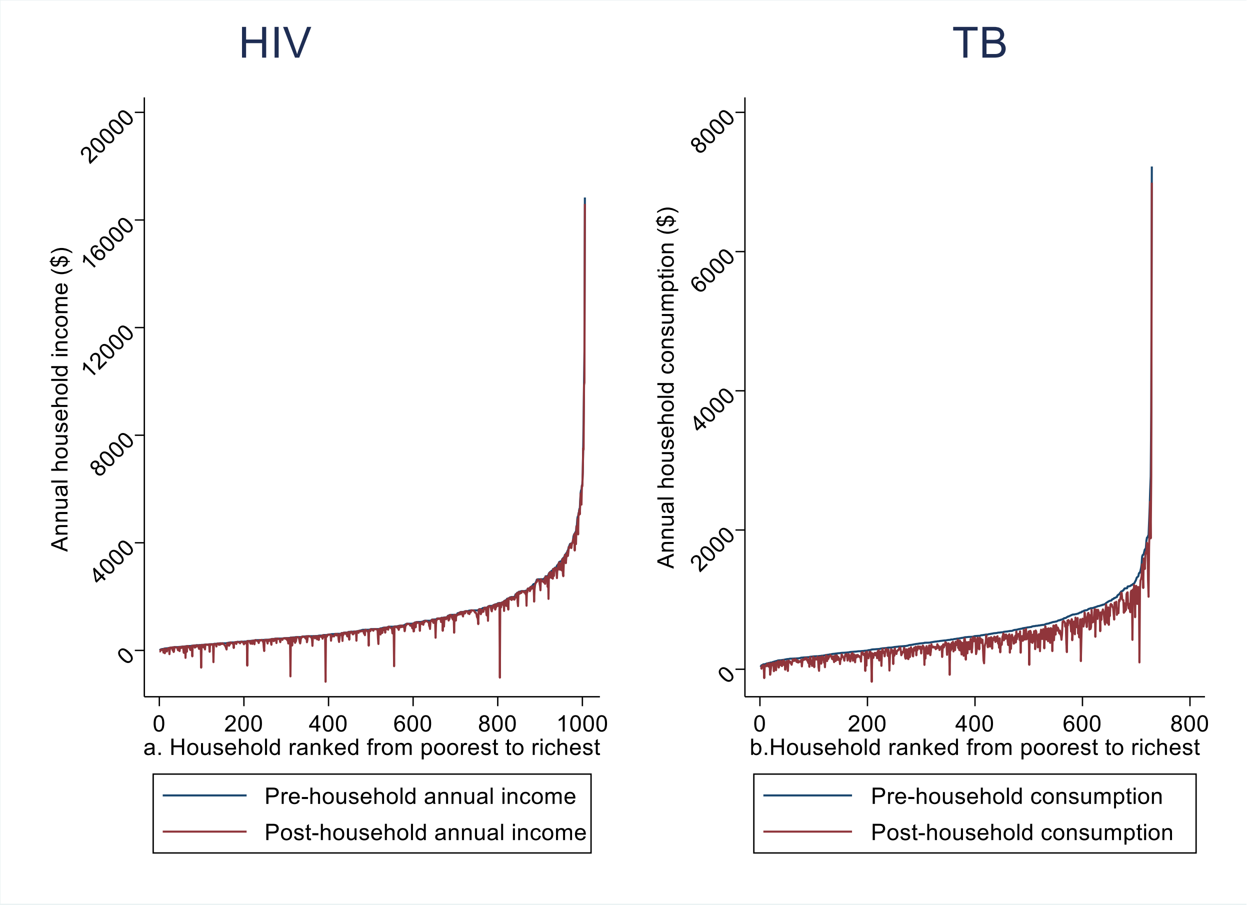

Figure 1. Pen's parade of household annual income/consumption gross and net of payments for HIV (a) and TB care (b) (Ethiopia).

For peer review only

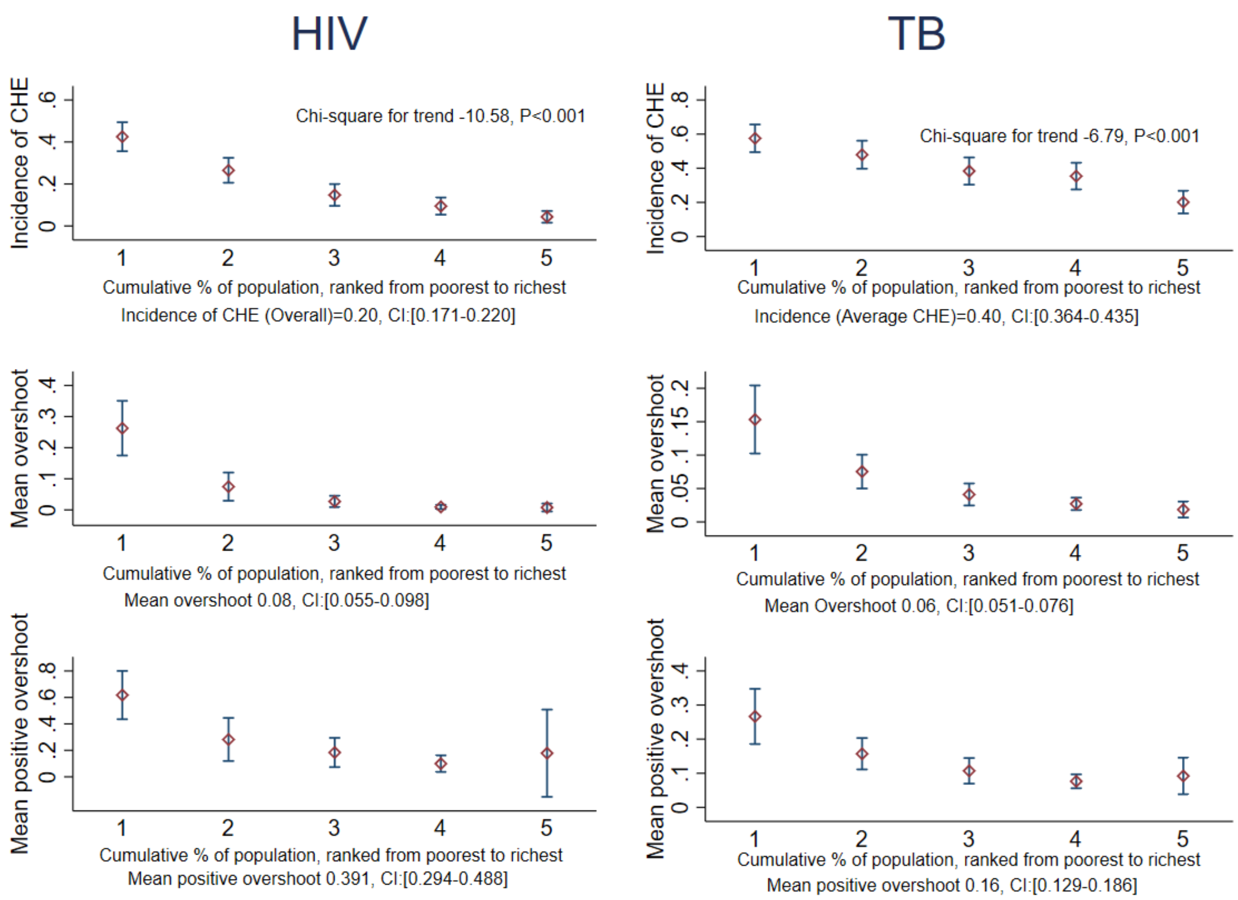

Figure 2. Interval plot (with 95% confidence interval for the mean) of incidence and intensity of HIV and TB-related catastrophic health expenditures (CHE) across income quintiles, using 10% threshold (Ethiopia).

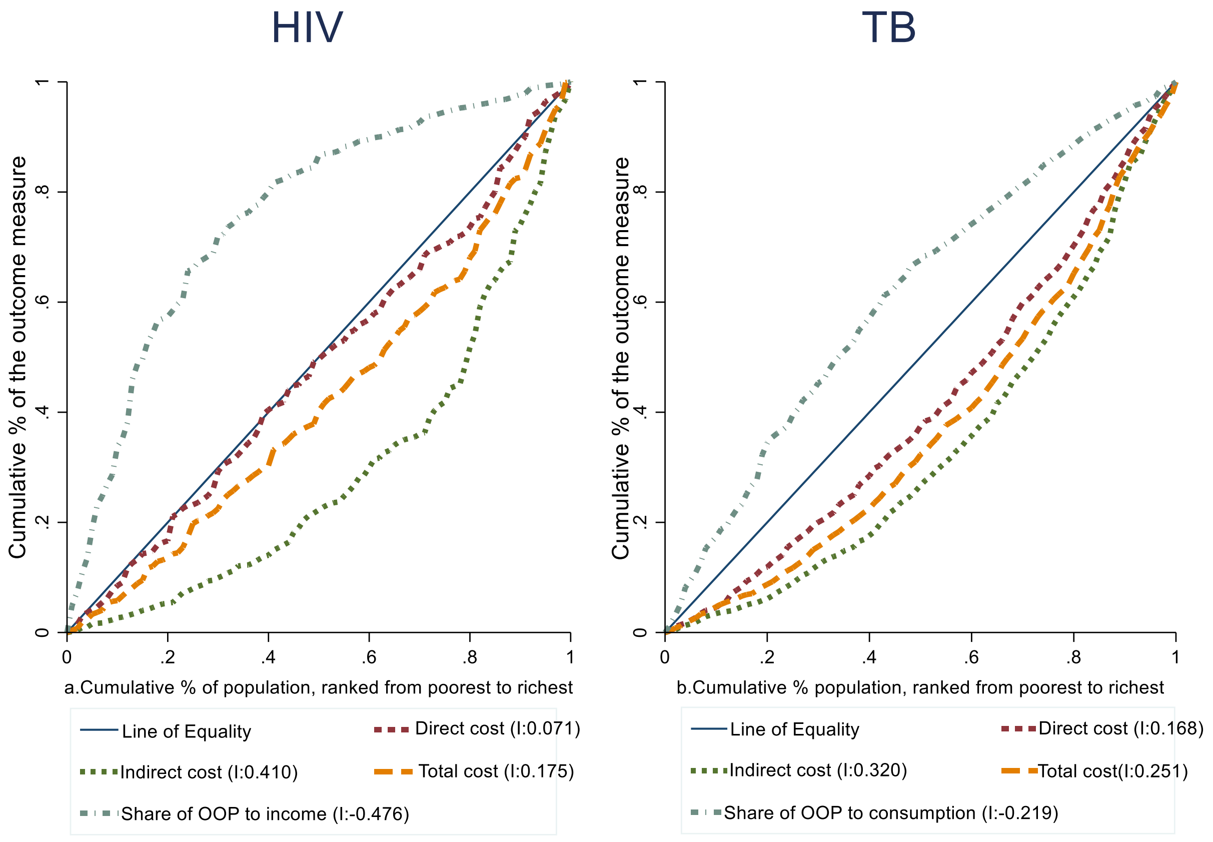

Figure 3. Concentrations curves and index (I) for direct, indirect, total cost and share of OOP to income/consumption for (a) HIV and (b) TB services in Ethiopia.

For peer review only - http://bmjopen.bmj.com/site/about/guidelines.xhtml

Supplementary appendix 1

The financial burden of HIV and TB among patients in Ethiopia: a cross-sectional survey

Lelisa Fekadu[1]*, Eyerusalem Negussie[2], Abdulrahman Jbaily[3], Mieraf Taddesse Tolla[3], Kjell Arne Johansson[1]

For peer review only

S1. Consumption construction

In this study, income (for HIV) and consumption as proxy for income (for tuberculosis) were used as household welfare measure. The consumption aggregates for tuberculosis (TB) was constructed using the overall value of the food (purchased food, home produced food) items, non-food items, and housing information collected at different recall periods (ranging from 1 to 12 months depending on how frequently the item purchased). Then, all reported expenditures on food items, non-food items, and housing were converted to a month period, then added up and multiplied by 12 to create annual consumption for each household.

The income (for HIV) and consumption (for TB) measures were also adjusted for family size and demographic composition to reflect economy of scale. We constructed per adult equivalence through dividing household income/consumption expenditure by an adult equivalent scale. Adult equivalent values (AE) were calculated using $AE = (A + \alpha K)^{\theta}$ for HIV, where A stands for number of adults, K is the number of children in the household, α is the cost of a child relative to an adult (0.33) and θ is the degree of economies of scale (0.9) (1). Whereas for TB, due to the unavailability of child data, we calculated using $AE = hsize^{\beta}$, where "$hsize$" is the actual household size and β was set to be 0.56 (2). Using per adult equivalence income (HIV) and consumption (TB) values, we grouped all households into five income quintiles of equal size.

For peer review only - http://bmjopen.bmj.com/site/about/guidelines.xhtml

S2. Incidence and intensity of catastrophic health expenditure (CHE)

S2.1. Measuring incidence of CHE (Headcount)

Catastrophic health expenditure (headcount, H) occurs when household OOP health spending exceeds a predefined threshold (10%) of household income/consumption. Furthermore, we conducted analysis at 20% threshold of both OOP and total cost (new definition of catastrophic cost recommended for TB), and at 40% of non-food expenditure (i.e. net of basic subsistence expenditure).

The CHE headcount is calculated using the equation

$$H = \frac{1}{N}\sum_{i=1}^{N} E_i \, ,$$

Where, $E_i = 1$ if $\frac{T_i}{X_i}$ or $\frac{T_i}{(X_i - FE_i)} > z$ and 0 otherwise.

Here, N equals the total sample size, T_i is the OOP health spending of household i, X_i is the total expenditure of household i, FE_i is the food expenditure of household i and z is the specified threshold.

S2.2. Measuring intensity of CHE

The intensity is measured using overshoot (O) and mean positive overshoot (MPO).

S2.2.1. Overshoot

The overshoot measures the extent of average expenditure exceeding the given threshold in the entire sample. The overshoot is calculated using the equation

$$O = \frac{1}{N}\sum_{i=1}^{N} (E_i \left(\frac{Ti}{Xi}\right) - z)$$

S2.2.2. Mean positive overshoot

For peer review only - http://bmjopen.bmj.com/site/about/guidelines.xhtml

The mean positive overshoot measures the extent of an average expenditure exceeding the threshold among households experiencing CHE, and is calculated by:

$$MPO = \frac{O}{H}$$

Table A1. Mean (median) patient costs per TB case across three types of TB (Ethiopia) expressed in $.

Income quintiles	Type of TB					
	Pulmonary[¥]		Extra-pulmonary TB[¥]		Drug resistant-TB	
	Mean (SD)	Median (IQR)	Mean (SD)	Median (IQR)	Mean (SD)	Median (IQR)
**Poorest**	46 (43)	32 (13-62)	60 (45)	46 (32-75)	295 (290)	139 (65-607)
**Poor**	67 (44)	56 (34-85)	107 (90)	83 (49-136)	159 (85)	153 (68-199)
**Middle**	102 (80)	75 (48-137)	115 (74)	94 (72-131)	205 (175)	156 (86-250)
**Rich**	133 (99)	102 (65-176)	155 (86)	133 (79-212)	534 (671)	291 (131-669)
**Richest**	175 (165)	121 (78-216)	262 (226)	212 (112-294)	971 (1356)	610 (201-821)
**Total***	104 (107)	73 (41-135)	140 (138)	96 (58-180)	446 (732)	191 (107-607)

* kruskal wallis test p-value <0.001

¥ CHE at 10% threshold ranged from 33% to 57% for pulmonary and extra-pulmonary TB, respectively.

Table A2. Incidence and intensity of CHE for HIV and TB across different income quintiles and threshold, 2019, Ethiopia

Disease	Measure Of CHE	Threshold	Average	Income Quantile				
				Q1	Q2	Q3	Q4	Q5
**HIV**	Out-of-pocket HIV spending: as a share of annual income							
	Head count (CHE incidence) %	10%	20	43	27	15	10	4
	Overshoot %	10%	7.6	26.2	7.5	2.7	0.9	0.9
	Mean positive overshoot %	10%	39.1	61.8	28.2	18.3	9.9	17.8
	Out-of-pocket HIV spending: as a share of annual income							
	Head count (CHE incidence) %	20%	11	30	14	6	3	1
	Overshoot %	20%	6.2	22.5	5.6	1.7	0.5	0.6
	Mean positive overshoot %	20%	58.0	74.9	38.6	28.2	18.2	122.0
	Total HIV spending: as a share of annual income							
	Head count (CHE incidence) %	20%	15	38	18	11	6	3
	Overshoot %	20%	7.2	25.4	6.4	2.5	0.8	0.8
	Mean positive overshoot %	20%	48.6	67.8	36.2	23.6	15.0	27.7
	Out-of-pocket HIV spending: as a share of total non-food expenditure							
	Head count (CHE incidence) %	40%	11	31	15	7	3	1
	Overshoot %	40%	13.1	47.4	11.8	3.6	1.0	1.2
	Mean positive overshoot %	40%	116.3	153.0	79.5	55.4	39.7	128
**TB**	Out-of-pocket TB spending: as a share of annual income							
	Head count (CHE incidence) %	10%	40	58	48	38	35	20
	Overshoot %	10%	6.3	15.8	7.5	4.1	2.7	1.9
	Mean positive overshoot %	10%	15.3	26.6	15.7	10.7	7.6	9.2

For peer review only - http://bmjopen.bmj.com/site/about/guidelines.xhtml

Out-of-pocket TB spending: as a share of annual income							
Head count (CHE incidence) %	20%	17	38	23	10	8	6
Overshoot %	20%	3.7	10.7	4.1	1.9	0.7	0.8
Mean positive overshoot %	20%	21.3	27.8	17.7	18.7	8.8	15.2
Total TB spending: as a share of annual income							
Head count (CHE incidence) %	20%	48	60	53	48	46	36
Overshoot %	20%	10.0	18.6	11.0	8.0	6.7	4.4
Mean positive overshoot %	20%	20.0	31.1	20.9	16.7	14.7	12.3
Out-of-pocket HIV spending: as a share of total non-food expenditure							
Head count (CHE incidence) %	40%	37	50	43	39	33	19
Overshoot %	40%	29.2	73.0	27.5	18.6	14.9	11.6
Mean positive overshoot %	40%	78.7	146	63.6	47.7	44.7	59.5
Where, Q1 = Poorest; Q2 = Poorer; Q3 = Middle; Q4 = Richer; Q5 = Richest.							

Figure A1. Average total cost of HIV and TB care across income quintiles in Ethiopia.

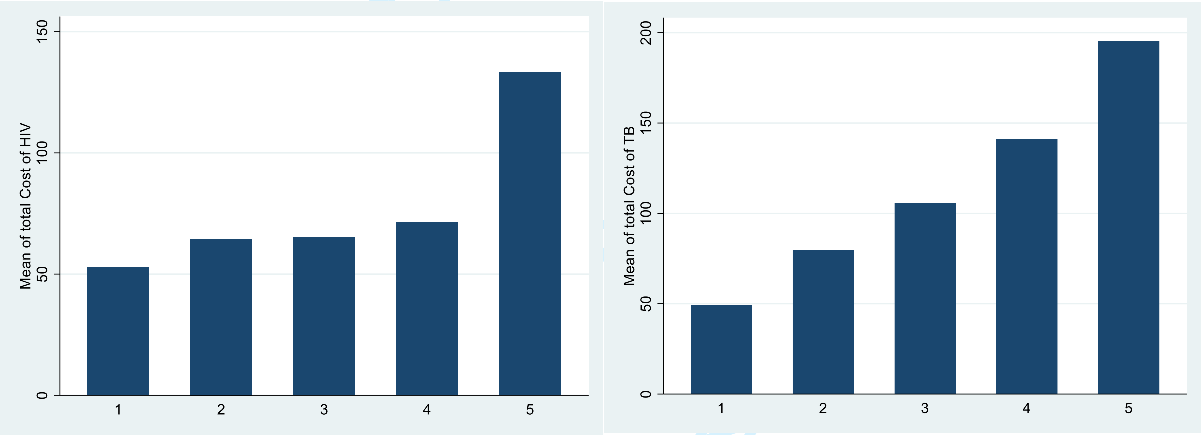

Figure A2. Out-of-pocket payment share curve for HIV and TB in Ethiopia.

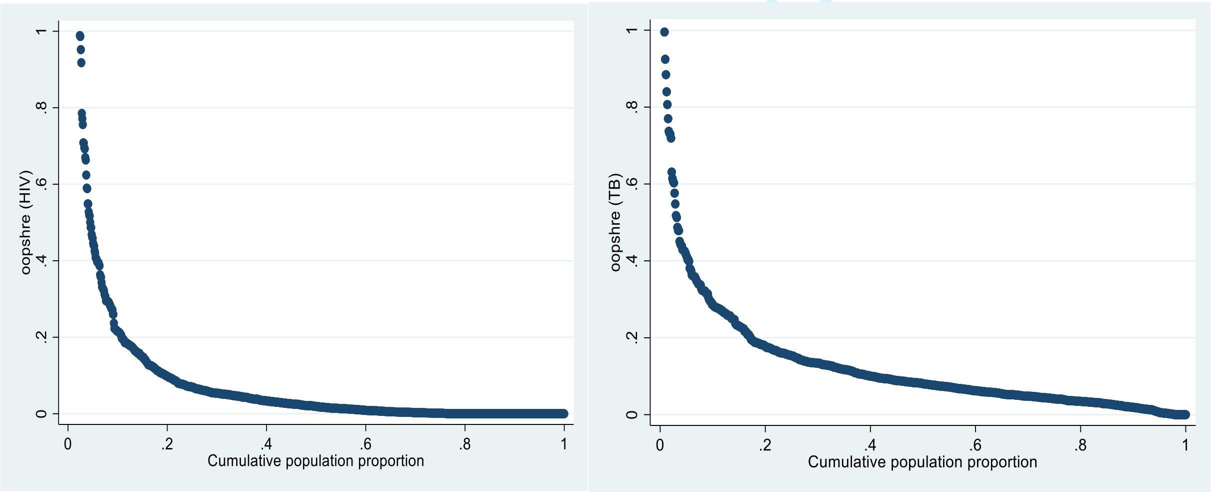

For peer review only - http://bmjopen.bmj.com/site/about/guidelines.xhtml

Figure A3. Receiver Operating Characteristic curve (ROC plot) for the prediction power of the HIV and TB model.

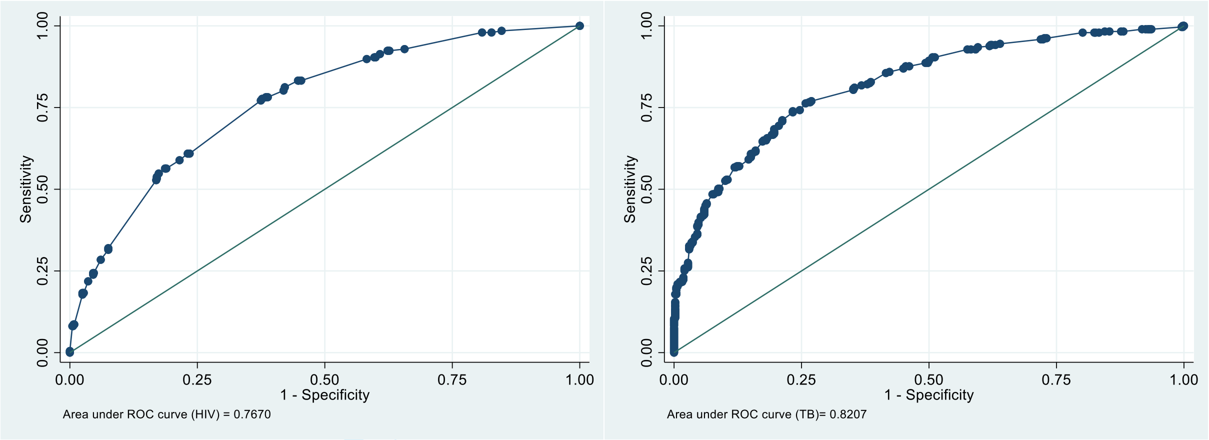

References

1. Deaton A, Zaidi S. Guidelines for constructing consumption aggregates for welfare analysis. 2002.
2. Xu K, Evans DB, Kawabata K, Zeramdini R, Klavus J, Murray CJ. Household catastrophic health expenditure: a multicountry analysis. Lancet (London, England). 2003;362(9378):111-7.

For peer review only - http://bmjopen.bmj.com/site/about/guidelines.xhtml

For peer review only

For peer review only - http://bmjopen.bmj.com/site/about/guidelines.xhtml

BMJ Open

The financial burden of HIV and TB among patients in Ethiopia: a cross-sectional survey

Journal:	*BMJ Open*
Manuscript ID	bmjopen-2020-036892.R2
Article Type:	Original research
Date Submitted by the Author:	13-Apr-2020
Complete List of Authors:	Assebe, Lelisa; University of Bergen Faculty of Medicine and Dentistry, Global Public Health and Primary Care Medicine; Ethiopia Ministry of Health, Disease Prevention and control<br>Negussie, Eyerusalem; Ethiopia Ministry of Health, Disease prevention and control program<br>Jbaily, Abdulrahman; Harvard University T H Chan School of Public Health, Global Health and Population<br>Tolla, Mieraf Taddesse; Harvard University T H Chan School of Public Health,Global Health and Population<br>Johansson, Kjell Arne; University of Bergen Faculty of Medicine and Dentistry, Department of Global Public Health and Primary care
**Primary Subject Heading**:	Health economics
Secondary Subject Heading:	Public health
Keywords:	Tuberculosis < INFECTIOUS DISEASES, HIV & AIDS < INFECTIOUS DISEASES, HEALTH ECONOMICS

SCHOLARONE™
Manuscripts

I, the Submitting Author has the right to grant and does grant on behalf of all authors of the Work (as defined in the below author licence), an exclusive licence and/or a non-exclusive licence for contributions from authors who are: i) UK Crown employees; ii) where BMJ has agreed a CC-BY licence shall apply, and/or iii) in accordance with the terms applicable for US Federal Government officers or employees acting as part of their official duties; on a worldwide, perpetual, irrevocable, royalty-free basis to BMJ Publishing Group Ltd ("BMJ") its licensees and where the relevant Journal is co-owned by BMJ to the co-owners of the Journal, to publish the Work in this journal and any other BMJ products and to exploit all rights, as set out in our licence.

The Submitting Author accepts and understands that any supply made under these terms is made by BMJ to the Submitting Author unless you are acting as an employee on behalf of your employer or a postgraduate student of an affiliated institution which is paying any applicable article publishing charge ("APC") for Open Access articles. Where the Submitting Author wishes to make the Work available on an Open Access basis (and intends to pay the relevant APC), the terms of reuse of such Open Access shall be governed by a Creative Commons licence – details of these licences and which Creative Commons licence will apply to this Work are set out in our licence referred to above.

Other than as permitted in any relevant BMJ Author's Self Archiving Policies, I confirm this Work has not been accepted for publication elsewhere, is not being considered for publication elsewhere and does not duplicate material already published. I confirm all authors consent to publication of this Work and authorise the granting of this licence.

For peer review only

Title

The financial burden of HIV and TB among patients in Ethiopia: a cross-sectional survey

Lelisa Fekadu Assebe[1]*, Eyerusalem Kebede Negussie[2], Abdulrahman Jbaily[3], Mieraf Taddesse Tolla[3], Kjell Arne Johansson[1]

1. Department of Global Public Health and Primary Care Medicine, University of Bergen, Bergen, Norway

2. Disease prevention and control program, Ministry of Health, Addis Ababa, Ethiopia

3. Department of Global Health and Population, Harvard T.H. Chan School of Public Health, Harvard University, Boston, MA, USA

*Correspondence: lelfekadu1@gmail.com

For peer review only

For peer review only - http://bmjopen.bmj.com/site/about/guidelines.xhtml

ABSTRACT

Objectives: Human Immunodeficiency Virus (HIV) and Tuberculosis (TB) are major global health threats and can result in household financial hardships. Here, we aim to estimate the household economic burden and the incidence of catastrophic health expenditures (CHE) incurred by HIV and TB care across income quintiles in Ethiopia.

Design: A cross-sectional survey.

Setting: 27 health facilities of Afar and Oromia regions for TB, and nationwide household survey for HIV.

Participants: A total of 1,006 and 787 individuals seeking HIV and TB care were enrolled, respectively.

Outcome measures: The economic burden (i.e. direct and indirect cost) of HIV and TB care was estimated. In addition, the CHE incidence and intensity was determined using direct costs exceeding 10% of the household income threshold.

Results: The mean (SD) age of HIV and TB patient was 40 (10), and 30 (14) years, respectively. The mean (SD) patient cost of HIV was \$78 (\$170) per year and \$115 (\$118) per TB episode. Out of the total cost, the direct cost of HIV and TB constituted 69% and 46%, respectively. The mean (SD) indirect cost was \$24 (\$66) per year for HIV and \$63 (\$83) per TB episode. The incidence of CHE for HIV was 20%; ranges from 43% in the poorest to 4% in the richest income-quintile (P <0.001). Similarly, for TB, the CHE incidence was 40% and ranged between 58% and 20% among the poorest and richest income-quintiles respectively (P <0.001). This figure was higher for drug resistant TB (62%).

Conclusions: HIV and TB are causes of substantial economic burden and CHE, inequitably, affecting those in the poorest income quintile. Broadening the health policies to encompass interventions that reduce the high cost of HIV and TB care, particularly for the poor, is urgently needed.

For peer review only - http://bmjopen.bmj.com/site/about/guidelines.xhtml

For peer review only

Strengths and limitations of this study

- This study will be of high value to policies aimed at universal health coverage for HIV and TB care in Ethiopia due to the financial risk of seeking care.
- Patient costs from this study can provide empirical bases for national HIV and TB programs for adopting public finance or health insurance-based financing strategies.
- The patient cost for outpatient and inpatient HIV and TB care are presented together with income and consumption levels of households.
- The cost measurements relied on a patient's ability to remember, which increases risk of recall bias.
- The HIV sampling consisted primarily of urban population and the TB study was limited to specific regions of the country, therefore, these samples are not necessarily nationally representative.

For peer review only - http://bmjopen.bmj.com/site/about/guidelines.xhtml

BACKGROUND

HIV and TB are major global health threats that cause a large financial burden on vulnerable populations. Global efforts, in the past two decades, have improved access to lifesaving HIV and TB interventions[1-3]. More than 72 million lives have been saved between 2000 – 2018[4, 5]. Nevertheless, high disease burden, inequality in utilisation of healthcare and service quality issues still exists[1-3]. Better understanding of factors that affect use of these services would help countries to achieve universal health coverage (UHC) of HIV and TB services.

The population in need for care is still large. Globally, 1.7 million people acquired HIV infection and 10 million new TB cases occurred in 2018. In the same period, more than 2 million people died from HIV and TB[4, 6]. In Ethiopia, the prevalence of HIV among adults was 1% (confidence interval (CI): 0.7%-1.4%) and the incidence rate of TB was 151/100,000 in 2018[4, 6]. In order to end HIV and TB, a comprehensive approach should include medical and non-medical interventions such as socio-economic support and poverty alleviation[7, 8].

Although many countries, including Ethiopia, offer ‘‘free’’ HIV and TB services, the implemented policies do not adequately provide realistic financial risk protection. The health budget in Ethiopia is low ($33.2 per capita) and mainly financed by out-of- pocket payment (OOP), which account for 31% of overall health finance[9, 10]. Hence, patient and their families, often face both direct and indirect costs, which create financial burden on households[11, 12]. A systematic review showed that individuals in low income countries spend a mean direct cost of $155 per drug susceptible TB and $406 per drug-resistant TB (DR-TB). The productivity losses were 2-3 times higher than the direct costs of drug susceptible and resistant cases, respectively[13]. Similarly, for HIV, spending ranges from $95 - $2,672 in sub-Saharan Africa[12]. Such high costs are related with catastrophic health expenditure (CHE), which occurs when the OOP exceeds 10% of annual income[14, 15] or 40% of household non-food expenditure[16]. In addition, OOP expenses for HIV and TB care may crowd out consumption

For peer review only - http://bmjopen.bmj.com/site/about/guidelines.xhtml

of basic needs and leave vulnerable households in debt/impoverishment[12, 17, 18]. Furthermore, high levels of patient cost may affect access to care, and lead to poor treatment outcome and prolonged period with infection[12, 14, 19-21].

Many factors may lead to CHE; exemptions of HIV and TB services often applies to limited aspects of the basic care package (e.g. $CD_{4,}$ viral load, Acid-fast bacilli, and Gene X-pert tests), treatment (e.g. antiretroviral therapy (ART), anti TB drugs). Patients pay for pre-diagnostic services, ancillary medications, basic laboratory testing, imaging, adverse event monitoring, hospitalization, transportation, food, lodging etc. [19, 22-24]. In addition, unavailability of diagnostic services in public health facilities pushes patients to seek care from expensive private providers[22]. Furthermore, low health insurance coverage (around 24% as of 2019), and repeated follow up visits were important contributing financial risk factors[25, 26]. It is also imperative that HIV and TB programs, needs to monitor household protection from CHE (i.e. financial risk protection) and its distribution across income groups (equity), as OOP health spending places greater burdens on the poor[27, 28].

In Ethiopia, few studies have been evaluating the extent of patient cost due to seeking HIV and TB care. The costs related to severe forms of the diseases and assessments across income or consumption groups were lacking from the studies reviewed[22, 23, 29]. Furthermore, none studied predictors of CHE[22, 23, 29]. Because HIV and TB are chronic diseases and intimately linked, it is reasonable to look at them jointly. This paper aims to estimate the economic burden and incidence of CHE incurred by standard HIV and TB care across income or consumption quintile among Ethiopian households. Moreover, we will assess factors associated with CHE for HIV and TB.

For peer review only - http://bmjopen.bmj.com/site/about/guidelines.xhtml

METHODS

Study setting and population

In this study, a nationwide household survey for HIV[10] and a cross sectional health facility based survey for TB, were used to estimate direct and indirect costs, and CHE.

Data for HIV were collected from mid-September to mid-October 2016. The total estimated number of PLHIV were 722,747 in Ethiopia. However, for TB, data were collected from December 2018 to September 2019 in three zones (i.e. zone 3 of Afar region, and Jimma and Adama special zones of Oromia region). The three zones were purposely selected and represents 4 million people mirroring the country's geographic and socio-economic heterogeneity. The zones account for 10% and 13% of the TB cases in Oromia and Afar regions (and 6% of the national prevalence), respectively.

Sample size and sampling technique

For HIV, PLHIV associations were used as a sampling frame to select HIV participants, as there is no national registry of PLHIV. The association operates in major cities in all regions and its members were primarily residents in urban areas. The estimated sample size was 4,200. The response rate ranges from 92% to 100% across regions. A two-stage stratified cluster-sampling method was employed. In stage one, a sample of 105 HIV associations from a total of 588 were randomly selected and allocated to each region using probability proportional to the size. In stage two, 40 HIV members from each sampled association, in total 4,171, were randomly selected and interviewed. Among the study participants (i.e. 4,171), 1,006 had HIV related care and were considered in our analysis.

For TB, the sample size was calculated using two-population proportion formula with 80% power, 5% type I error, 95% confidence level, and using 39%[21] of households incur CHE among the richest income quartile; and to detect 15% point difference revealed 186 samples for each quartile. The final sample size with 10% non-response rate was 818 (of which 7% were DR-

For peer review only - http://bmjopen.bmj.com/site/about/guidelines.xhtml

TB). Systematic random sampling was employed to select 27 public health facilities from the three zones. The total sample size was distributed proportional to the TB case load[30].

Patient and public involvement

The research question of this study is in line with the Ethiopian tuberculosis research plan developed through multiple consultative processes involving broader stakeholders including patient representatives. We plan to disseminate the research findings through the national TB research conferences involving researchers, policy makers, stakeholders and affected communities.

Data collection tools and quality assurance

The data collection was based on a structured questionnaire adapted from the United States Agency for International Development (USAID) for HIV and the World Health Organization (WHO) patient costing tool for TB[31, 32]. The questionnaire captures sociodemographic variables, direct cost, indirect cost, productivity loss, assets, income, consumptions and coping related information. We complemented clinical information for TB through review of medical records. The questionnaire was translated into local languages, pre-tested and modified accordingly. Trained data collectors under close supervision undertook the face-to-face interview. TB patients with a minimum of one month on treatment were interviewed consecutively and the expenses were reported retrospectively. This schedule of interview is based on WHO's recommendation regarding the cost survey of TB patients[28, 31]. Whereas, for HIV, the expenditure in the past four weeks for outpatient and six months for inpatient care were gathered.

Patient cost

The costs of seeking standard HIV and TB care were estimated from patient perspective. The expenditure related to routine care, HIV-related opportunistic infection, and managing co-

For peer review only - http://bmjopen.bmj.com/site/about/guidelines.xhtml

morbidities was considered for HIV. Likewise, for TB episode, the expenditure in the pathway to care from onset of symptoms, diagnosis and completion of treatment was included.

Direct costs were household expenditures for medical (i.e. registration/consultation fees, laboratory tests, X-ray, medicines, hospital admission) and non-medical services (i.e. special food/nutrition, transportation, and guardian cost) net of reimbursement. Indirect costs constitutes lost income following the disease episode. In order to estimate the indirect cost, patients were asked to estimate the time lost due to receiving and waiting for care, hospitalization, transportation, lost working days and guardian time (TB). Then the total time lost was multiplied by an hourly wage rate, which was derived from monthly income/consumption by assuming 22 working days a month and 8 hours a day. For children less than 15 years of age, non-medical direct and indirect TB costs was computed for the guardian. Total patient cost is the sum of all direct and indirect costs.

In order to annualize the cost, an average of four HIV outpatient visits and single TB episode outpatient visits was considered. The frequency of outpatient HIV visits per year was extrapolated on the basis of per capita health care visits an individual made over the last one month among all PLHIV interviewed. This proportion was annualized to an approximately 4 visits per patient and year. The TB patient cost was extrapolated for the whole duration of TB episode based on an individual data reported retrospectively. All costs were gathered in local currency (Ethiopian Birr) and converted to US dollar ($) with the 2019 exchange rate of $ 1= 29.1468 Ethiopian Birr[33]. For HIV, the cost was first converted to a reference period (2019) using Ethiopia's consumer price index[34]. Due to unavailability of data, we used household income (for HIV) and consumption aggregates (for TB) as a proxy for the household welfare measure and scaled to per adult equivalence (Supplementary appendix 1). In addition, participants were grouped into five-income/consumption quintiles to reflect the socio-economic strata (Supplementary appendix 1).

For peer review only

For peer review only - http://bmjopen.bmj.com/site/about/guidelines.xhtml

An incidence of CHE occurs when direct costs (i.e. OOP) exceed a 10% threshold of annual household income/consumption[14, 15, 17]. In addition to the 10% threshold, we carried out further analysis at 20% threshold of both OOP and total cost, and at 40% of non-food expenditure to allow comparison [16, 21, 31, 35]. Furthermore, the distribution of financial burden (measured as ratio of direct/total costs to total household expenditure) across income quintile was reported using headcount, overshoot and mean positive overshoot[36] (Supplementary appendix 1).

Data analysis

Data was analysed using Stata version 16 software. The data was summarized using mean with standard deviation (SD) or median with interquartile range (IQR) due to skewed distribution. The cost was disaggregated by outpatient and inpatient care. A concentration index was used to assess health outcome measure inequality across income quintiles[36, 37]. Multivariate logistic regression was conducted to identify determinants of CHE by including significant variables in univariate analysis. A stepwise regression approach was employed to develop the final model and adjusted odds ratios (aOR) with 95% CI was reported. P-value less than 0.05 declares statistical significance for each tests. Multicollinearity was ruled out (variance inflation factor <5). Goodness of fit was checked by Hosmer–Lemeshow test[38].

Ethical consideration

The study was approved by medical and health research ethics in Norway (2018/1647/REK) and Ethiopian public health institute (EPHI-IRB-121-2018). Informed written consent was obtained from TB participants. Oromia and Afar regional health bureau, and respective zonal health offices provided permission to undertake the TB study.

For peer review only

For peer review only - http://bmjopen.bmj.com/site/about/guidelines.xhtml

RESULTS

Out of the total 1,006 HIV and 787 TB participants, 75% of HIV and 50% of TB were females (Table 1). More than two thirds of the study participants were in the age group between 25-44 years for HIV and 18-34 years for TB. The median family size was 4 (IQR, HIV: 3–5, IQR, TB: 3-6). The mean (SD) household annual income/consumption per adult equivalence was $1,188 ($1,288) for HIV and $545 ($462) for TB. Seven percent of TB patients were co-infected with HIV. Almost all (99%) of HIV and 91% of TB/HIV co-infected were receiving ART. About 22% and 6% of HIV and TB patients had both outpatient and inpatient care, respectively. The mean hospital stay was 11 days for HIV and 10 days for TB. The mean (SD) time interval from first health care visit to TB diagnosis (health system delay) was 14 (38) days and 85% of TB were diagnosed in public health facilities.

Table 1: Socio-demographic and clinical characteristics of HIV and TB study participants (Ethiopia).

**Background characteristics**		**HIV (N=1006)**	**TB (N=787)**
		**N (%)**	**N (%)**
Gender	Female	756 (75)	396 (50)
Age in years (mean, SD)		40 (10)	30 (14)
Age group	<18	-	110 (14)
	18-24	14 (1)	190 (24)
	25-34	268 (27)	229 (29)
	35-44	435 (43)	117 (15)
	45-54	202 (20)	86 (11)
	55-64	58 (6)	32 (4)
	65+	29 (3)	23 (3)
Marital status	Single	38 (4)	309 (39)
	Married/Living together	493 (49)	409 (52)
	Widowed	277 (27)	24 (3)
	Divorced	137 (14)	33 (4)
	Separated	61 (6)	11 (2)
Place of residence	Urban	930 (92)	394 (50)
	Rural	76 (8)	393 (50)
Highest level of education	Illiterate	290 (29)	257 (33)
	Elementary	457 (45)	334 (42)
	Secondary and Higher	259 (26)	195 (25)
Family size	≤ 4	640 (64)	443 (56)
	>4	366 (36)	344 (44)
Annual household	Lowest	200 (20)	159 (20)

For peer review only - http://bmjopen.bmj.com/site/about/guidelines.xhtml

income/consumption	Second	470 (21)	298 (20)
	Middle	797 (18)	443 (20)
	Fourth	1,342 (20)	631 (20)
	Highest	3,084 (21)	1,198 (20)
ART status	On ART	996 (99)	52 (91)
	Not on ART	10 (1)	5 (9)
Past history of illness*	Yes	1,006 (24)	61 (8)
	No	3,165 (76)	726 (92)
Type of visit	Outpatient	790 (79)	739 (94)
	Inpatient	216 (22)	47 (6)
Number of visits per year/TB episode	Outpatient	4,428 (4 visit/patient)	48,720 (70 visit/patient)**
	Inpatient	249 (1 visit/patient)	2,409 (73 visit/patient)**
Type of TB	Pulmonary TB	-	507 (65)
	Extra-pulmonary TB	-	222 (28)
	Drug resistant TB	-	57 (7)

*HIV related comorbidities (HIV), and history of TB (TB)

** The number of daily visits per patient reaches 125 for outpatient and 135 for inpatient drug resistant TB cases.

Patient cost of HIV and TB care

The total mean (SD) patient cost for HIV care was $78 ($170) per year and $115 ($118) for the entire duration of a TB episode (Table 2). The mean (SD) direct cost was $54 ($144) for HIV and $53 ($59) for TB, which constitutes 69% and 46% of the total cost, respectively. Medical costs contributed to 68% and 38% of the direct costs for HIV and TB, respectively. Diagnostics and medicine account for 39% of the total HIV cost. The mean (SD) indirect cost was $24 ($66) for HIV per year, and $63 ($83) per TB episode. The productivity loss related to TB follow-up visits accounts for 36% of the total cost.

The mean (SD) total cost was $63 ($165) for annual outpatient HIV care visit and $110 ($114) for the whole duration of outpatient TB care (Table 2). Similarly, the mean (SD) total cost for each hospitalization was $96 ($139) for HIV and $105 ($78) for TB. For patients having both outpatient and inpatient visits, the mean cost reaches $133 ($178) ($p<0.001$) for HIV and $ 217 ($157) ($p<0.001$) for TB.

For TB, the patient costs incurred prior to initiation of treatment is equal to the cost from initiation to completion of TB treatment (paired t-test>0.05). The mean (SD) total cost for those with

For peer review only

For peer review only - http://bmjopen.bmj.com/site/about/guidelines.xhtml

TB/HIV co-infection reached $188 ($33). Similarly, the total cost of care significantly varies by type of TB, was $104 ($107) for pulmonary, $140 ($138) for extra-pulmonary and $446 ($732) for DR-TB (Kruskal Wallis test 41.1, P <0.001) (Supplementary appendix 1: Table A1).

Coping costs

HIV and TB care results in adverse financial consequences for households. Nearly 24% of HIV and 68% of TB study participants have adopted coping mechanisms. Nine percent of HIV and 4% of TB patients have borrowed money; while, 2% of HIV and 19% of TB patients sold their household assets. Furthermore, 12% of HIV patient relied on family assistance and 16% of TB patient used their savings to cope with the costs. Only 2% of HIV and 6% of TB participants are covered by health insurance.

Table 2: Distribution of household direct, indirect and total cost of HIV and TB care across main cost category in Ethiopia (expressed in $).

Cost category ($)		HIV (N=1,006)			TB (N=729)		
		Outpatient (Per year)	Inpatient (Per single visit)	Total	Outpatient (Per TB episode)	Inpatient (Per single visit)	Total
**I. Direct medical cost**							
Consultation fee	Mean (SD)	6 (15)	1 (5)	6 (15)	2 (5)	11 (16)	3 (7)
	Median (IQR)	0 (0-6)	0 (0-1)	0 (0-5)	1 (0-2)	7 (1-14)	1 (0-2)
Investigation cost	Mean (SD)	14 (45)	9 (19)	15 (47)	8 (13)	14 (16)	9 (15)
	Median (IQR)	0 (0-11)	0 (0-9)	1 (0-12)	4 (0-12)	7 (3-18)	5 (0-13)
Drug cost[1]	Mean (SD)	12 (40)	22 (63)	16 (48)	7 (14)	16 (21)	8 (16)
	Median (IQR)	0 (0-10)	4 (0-19)	1 (0-13)	1 (0-10)	8 (3-20)	2 (0-10)
**Subtotal**	Mean (SD)	32 (88)	32 (77)	37 (98)	17 (24)	40 (37)	20 (30)
	Median (IQR)	4 (0-34)	8 (0-32)	6 (0-38)	10 (1-22)	30 (16-45)	11 (1-25)
**II. Direct non-medical cost**							
Transportation fee	Mean (SD)	11 (55)	19 (34)	13 (53)	8 (10)	7 (7)	8 (10)
	Median (IQR)	2 (0-8)	6 (0-20)	3 (0-9)	5 (2-11)	7 (3-9)	5 (2-11)
Food/accommodation	Mean (SD)	3 (50)	10 (30)	5 (48)	23 (29)	28 (34)	25 (31)
	Median (IQR)	0	0 (0-5)	0	15 (7-27)	16 (7-38)	16 (7-29)
**Subtotal**	Mean (SD)	14 (80)	29 (49)	17 (76)	31 (34)	35 (36)	33 (37)
	Median (IQR)	2 (0-8)	10 (0-38)	3 (0-10)	21 (11-38)	20 (9-44)	21 (12-40)
**Total direct cost**	Mean (SD)	46 (142)	60 (113)	54 (144)	48 (50)	75 (68)	53 (59)
	Median (IQR)	12 (1-45)	22 (4-74)	15 (2-57)	35 (17-61)	54 (26-94)	36 (18-64)
**III. Indirect cost**							
Foregone income before treatment[2]	Mean (SD)	0	0	0	11 (28)	0	11 (26)
	Median (IQR)	0	0	0	0 (0-5)	0	0 (0-3)
Foregone income during treatment	Mean (SD)	11 (44)	22 (41)	16 (50)	11 (26)	12 (31)	10 (26)
	Median (IQR)	0	8 (3-19)	0 (0-8)	0 (0-4)	0	0 (0-3)

For peer review only

For peer review only - http://bmjopen.bmj.com/site/about/guidelines.xhtml

Time loss related cost	Mean (SD)	6 (19)	14 (52)	8 (30)	42 (59)	18 (24)	42 (58)
	Median (IQR)	2 (1-4)	2 (1-7)	2 (1-5)	27 (14-49)	8 (5-22)	27 (14-49)
**Subtotal**	Mean (SD)	17 (54)	35 (75)	24 (66)	62 (84)	30 (37)	63 (83)
	Median (IQR)	3 (1-8)	13 (5-32)	4 (2-17)	36 (17-77)	9 (5-39)	36 (17-78)
**IV. Total cost**	Mean (SD)	63 (165)	96 (139)	78 (170)	110 (114)	105 (78)	115 (118)
	Median (IQR)	20 (5-63)	52 (25-109)	27 (7-80)	79 (46-140)	87(39-158)	81 (47-150)

[1]Drug other than anti-retroviral and anti TB drugs
[2]Not captured in HIV survey

As shown in figure 1, the spikes in the pen's parade graph revealed that the health care costs of HIV and TB cause a large decrease in annual income/consumption for many of the households. The consumption drop is more pronounced for TB than the income drop for HIV (Figure 1).

[Figure 1 here]

As shown in Table 3, there are inequalities in OOP costs among HIV and TB participants. The total cost rises steadily across the income quintiles and is concentrated among the richer quintiles (i.e. the richer the quintile, the higher the cost). From the lowest to the highest income/consumption quintile, the mean (median) total cost of HIV increases from \$53 (\$20) to \$133 (\$60), with significant difference among income quintiles (Kruskal Wallis test 44.7, P < 0.001), and for TB the cost increases from \$50 (\$36) to \$202 (\$148) (Kruskal Wallis test 206.5, P < 0.001). In general, the median cost (both direct and indirect) seems to be a bit higher for TB patients as compared to those with HIV.

Table 3. Mean (median) HIV and TB patient costs per year across income quintiles in Ethiopia (expressed in \$).

Disease Category	Income quintiles	Cost type					
		Direct		Indirect		Total cost	
		Mean (SD)	Median (IQR)	Mean (SD)	Median (IQR)	Mean (SD)	Median (IQR)
**HIV**	Poorest	45 (92)	14 (2-48)	8 (15)	2 (1-7)	53 (97)	20 (3-61)
	Poor	55 (170)	13 (0-52)	10 (19)	3 (1-9)	65 (173)	22 (4-65)
	Middle	50 (118)	19 (3-49)	16 (30)	3 (2-16)	66 (136)	32 (7-65)
	Rich	47 (89)	15 (3-57)	25 (51)	4 (3-20)	71 (111)	27 (7-81)
	Richest	73 (206)	19 (3-79)	60 (124)	14 (5-59)	133 (262)	60 (12-132)
**P-value***		0.358		<0.001		<0.001	
**TB**	Poorest	31 (35)	23 (7-41)	19 (20)	11 (6-24)	50 (44)	36 (20-66)
	Poor	44 (48)	27 (15-54)	36 (32)	27 (13-45)	79 (65)	60 (38-96)

For peer review only - http://bmjopen.bmj.com/site/about/guidelines.xhtml

	Middle	49 (53)	36 (17-61)	57 (46)	40 (22-82)	106 (79)	82 (57-137)
	Rich	61 (48)	49 (28-84)	79 (63)	58 (27-117)	140 (96)	118 (68-181)
	Richest	78 (86)	54 (34-99)	124 (145)	70 (40-163)	202 (189)	148 (88-260)
**P-value***		< 0.001		< 0.001		< 0.001	

*kruskal wallis test

Incidence and intensity of CHE

At the 10% threshold, the overall CHE incidence of HIV was 20% (197 households); with 43% of the poorest and 4% of the richest household experiencing CHE (Chi-square for trend -10.58, P <0.001). The incidence is 33% for individuals with inpatient HIV care. The corresponding level of TB was 40% (291 households); with 58% and 20% of the poorest and richest income-quintile experienced CHE, respectively (Chi-square for trend -6.79, P <0.001) (Table 4). The incidence was much higher for those with TB/HIV co-infection (48%), DR-TB (62%) and was almost universal (94%) for hospitalized TB patient. At the 20% threshold of total expenses, 48% (353 households) of TB households experienced catastrophic total costs (Figure 2, Supplementary appendix 1: Table A2).

In our study, for example, the mean overshoot of TB related CHE was 6.3% (range: 1.9%-15.3%). On average, households spent 6.3% beyond the 10% threshold for TB care. However, the average positive overshoot among households that experienced CHE was 15.8% (range: 9.2% - 26.6%). Thus, on average, households that experienced CHE spent 25.8% (10% threshold + mean positive overshoot) of their total annual consumption for TB care (Figure 2, Supplementary appendix 1: Table A2).

[Figure 2 here]

Inequality in financial risk

In addition, as shown in figure 3, inequality in financial risk across income/consumption groups exists. The concentration curves for HIV and TB care costs lies below the 45° line of equality, which shows a greater concentration of the costs among the rich. However, the financial burden

For peer review only

For peer review only - http://bmjopen.bmj.com/site/about/guidelines.xhtml

is higher among the poor – the concentration curves for HIV and TB care expenditure in relation to income/consumption lies above the 45° line of equality.

[Figure 3 here]

Determinants of CHE

In the multivariate analysis (Table 4), three variables were independently associated with HIV related CHE: hospitalized patients (aOR: 3.3, 95% CI: 2.2-4.9), being poorest (aOR: 18.4, 95% CI: 8.9-37.7) and poor (aOR: 9.4, 95% CI: 4.5-19.5) were associated with catastrophic HIV care expenditures. Moreover, every additional visit for HIV care increases the odds of CHE by 7% (aOR: 1.07, 95% CI: 1.003-1.1).

Seven variables were significantly associated with TB related CHE (Table 4): private facility diagnosis (aOR: 2.6, 95%CI: 1.52-4.33); extra-pulmonary TB (aOR: 2.6, 95%CI: 1.77-3.95); hospitalized patients (aOR: 30.6, 95% CI: 4.77-199.83); being poorest (aOR: 14.6, 95% CI: 7.49-28.26); and TB/HIV co-infection (aOR: 3.2, 95% CI: 1.63-6.15) were very likely to have TB related CHE as compared to their counterparts after adjusting for other variables. Every additional visit for TB diagnosis increases the odds of experiencing CHE by 2.4 times (aOR: 2.4, 95% CI: 1.92-3.05). Households with a health insurance scheme have protection from CHE (aOR 2.7; 95%CI 1.06–6.73).

Table 4. Multivariate logistic regression model of determinants of CHE for TB and HIV care at a 10% threshold of household income/consumption (Ethiopia).

**TB**			**HIV**		
**Variable**	**aOR (95% CI)**	**P-value**	**Variable**	**aOR (95% CI)**	**P-value**
Frequency of visits[¥]	2.4 (1.9-3.1)	<0.001	Frequency of visits Per year[¥]	1.07 (1.003-1.1)	0.040
Hospitalization			Hospitalization		
No	Ref.		No	Ref.	
Yes	30.6 (4.8-199.8)	0.001	Yes	3.3 (2.2-4.9)	<0.001
Income quintiles			Income quintiles		
Richest	Ref.		Richest	Ref.	
Rich[£]	4.1 (2.1-7.8)	<0.001	Rich	2.5 (1.1-5.8)	0.025
Middle	4.9 (2.5-9.4)	<0.001	Middle	4.5 (2.1-9.8)	<0.001

For peer review only - http://bmjopen.bmj.com/site/about/guidelines.xhtml

Poor	7.0 (3.6-13.7)	<0.001	Poor	9.4 (4.5-19.5)	<0.001
Poorest	14.6 (7.5-28.3)	<0.001	Poorest	18.4 (8.9-37.7)	<0.001
Place of diagnosis					
Government	Ref.				
Private	2.6 (1.5-4.3)	<0.001			
TB/HIV co-infection					
No	Ref.				
Yes	3.2 (1.6-6.2)	0.001			
Insurance (i.e. CBHI)					
Yes	Ref.				
No	2.7 (1.1-6.7)	0.038			
Type of TB					
Bacteriologically confirmed TB	Ref.				
Clinically diagnosed TB	1.6 (1.0-2.8)	0.075			
Extra pulmonary TB£	2.6 (1.8-4.0)	<0.001			

¥ Variable treated as continuous
£Overall test is significant
CBHI: Community based health insurance

For peer review only

DISCUSSION

In this study, we tried to estimate the OOP, total cost, incidence and determinants of CHE among individuals seeking HIV and TB care. This could provide valuable insight into the level of financial risk protection that a health system offers to its population.

HIV care costs and CHE

In Ethiopia, where ART is given free-of-charge, PLHIV had to pay a total of $78 per year for HIV care ($19.5 per visit). The total HIV patient cost was equivalent to 7% of their annual income. As HIV requires lifelong care, such cost have devastating impact on affected households. Our estimate was lower than the level and rate found in Cameron 17%[39], Ethiopia 21%[23], Nepal 28.5%[40] and South Africa (30%)[41]. These variations might arise from different study settings[39, 40]; high expenses of additional food and time loss[41]. Similarly, more centralized HIV service delivery, delays in seeking care, and long distance travel to access services may explain the difference with the previous study from Ethiopia. Additionally, the annual income in this study was twice the latter study[23]. However, a study on outpatient HIV care in Nigeria reported one-fourth of the cost in this study ($21.76)[42].

In our study, the average direct OOP expenditure for HIV care was $54 (i.e. 69% of the total cost) and comparable with previous studies from Lao, Ethiopia and Nepal[19, 23, 40]. Diagnostics, medicines and transportation costs constitute the largest share, and may pause a serious challenge to the success of the HIV program. This calls for public financing policies (i.e. free of charge diagnosis and treatment of HIV related comorbidities for vulnerable groups) in the next steps of UHC expansion. The productivity loss found here was one half of the previous studies in Ethiopia and Nepal,[23, 40] but one fourth of that of Lao study[19]. The difference in productivity losses with previous Ethiopian study could be attributed to a more centralized provision of HIV services and backlog of patients with advanced HIV diseases, unlike this study. The total costs of HIV care increases as income rises. The equity ratios (Q1:Q5, 0.39) showed

For peer review only - http://bmjopen.bmj.com/site/about/guidelines.xhtml

that higher expenditure among the richest income quintile, which is consistent with a study from southeast Nigeria[43].

In our study, about a fifth (20%) of patients seeking HIV care experienced CHE. A study from India also depicts similar findings[44]. However, the rate is lower than a study from Cameroon[39]. Furthermore, the incidence of HIV related CHE remained relatively high; in particular, where poorest households suffer more. Similar socio-economic inequality was observed in previous studies,[42, 43] highlighting the importance of rendering equitable access to all in need of HIV care, particularly for the poor[39]. Consistent with previous studies, being poor is associated with higher CHE[19, 45]. In addition, the poor were pushed further beyond the CHE threshold than the better off. This is of great concern, as health shocks are slightly managed by the poor through reduction of basic requirements to compensate for HIV care. Similar to previous studies, being hospitalized was a stronger determinant of CHE[19, 42].

TB care costs and CHE

In our study, patients with TB incurred a total cost of $115 per episode and represents 21% of the annual household income, comparable to a study in South Africa (22%)[46]. However, the total cost was less than the figure reported by previous studies in Ethiopia[22, 29], but twice higher than the finding from southern Ethiopia[47]. The variation in cost from previous studies arises from high expenditure on nutritional supplements ($72 vs $25)[29], lower level of seeking diagnostic care from public health facilities (64% vs 85%), high rates of clinically diagnosed cases (49% vs 24%), diagnostic delays result in longer losses of working days [22], only direct cost captured[47]. The average patient costs of DR-TB care were four times higher than drug susceptible TB care. However, the DR-TB cost was three times lower than findings from another Ethiopian study[48]. Still the devastating nature of DR-TB may put them at special risk of CHE. The lower costs is mainly due to decentralization of DR-TB services in recent years and the

For peer review only - http://bmjopen.bmj.com/site/about/guidelines.xhtml

introduction of shorter DR-TB treatment regimen (i.e. 71% the study participants took 9-12 month regimen).

In this study, one-half of the mean TB cost was incurred prior to initiation of treatment, which was consistent with many previous studies[11, 21, 22, 49, 50]. This is mostly due to payment while demanding for proper diagnosis of TB[21]. In addition to the financial burden of high pre-treatment costs, it will be a barrier to complete the diagnostic process, and to timely access treatment and care. These emphasize the need for early case finding with rapid and point of care TB diagnostics, involvement of private care providers, and instituting effective referral and linkage[47].

The direct patient costs incurred constitutes 46% of the total, comparable with findings from elsewhere (36%) [51] and systematic review result (40%)[11]. However, the proportion was higher than the finding from southwestern Ethiopia (29%) and lower than the report from central Ethiopia (71%)[22, 29]. Consistent with previous studies, non-medical and indirect costs represent a large share of the TB cost, while medical cost represent less than 20%[11, 22, 41]. Therefore, ensuring the expansion of TB service package through the effective integration of a health insurance scheme and decentralization of services can reduce the direct costs.

A higher percentage of households incur CHE for TB care (40%), which was comparable with studies from Fiji (40%), Ghana (47.6%), China (53%), Philippines (35%) and lower than reported rates from Ethiopia (63%), Nigeria (65%) and Benin (72%)[29, 49, 50, 52-54]. However, CHE for TB care was higher in this study compared to findings in studies from India (21%) and Malaysia (6%)[51, 55]. This variation might arise from cost estimation method, study setting, health system and socioeconomic differences. The TB related CHE was higher than the reported rate for HIV. The main reasons for this are the differences in treatment duration, follow-up frequency and care access between HIV and TB. TB patients experience a very onerous set of direct and indirect costs during diagnostic and intensive phase of directly observed short course

For peer review only - http://bmjopen.bmj.com/site/about/guidelines.xhtml

therapy (i.e. more intense for retreatment, extra-pulmonary and DR-TB cases). After treatment completion and possible sputum conversion, TB patients are less likely to face additional costs. Even though the annual cost of HIV care is lower, PLHIV faces these costs over its lifetime because HIV infection is a chronic disease that needs lifelong treatment. The comparison of incidence and intensity of CHE for TB and HIV is also complicated by the use of income for HIV and consumption for TB-related computations. In developing countries, income is poor self-reported estimator of welfare due to more common informal employment, seasonal agricultural activities and widespread reluctance to disclose income[15]. Therefore, income could understate the welfare of the household, whereas using consumption may overstate the condition of the household because of the use of dissaving/borrowing to smooth consumption over time[56].

The mean overshoot for TB was 6.3% and the MPO was 15.3%, both were similar with finding from Benin (7.8% and, 14.8%) and Nigeria (6.0% and, 9.3%)[50, 52]. TB, inequitably, imposes a greater burden of CHE on the poor households. Even though poor households tend to spend less, a higher share of their income is spent on seeking TB care[12, 29, 46, 50, 52]. In addition, the excess CHE beyond the threshold was inequitably high among the poor. This finding is also in line with other studies[29, 50, 52]. Similar to other studies, hospitalization, income status, and TB/HIV coinfection were among the key determinants of TB related CHE[28, 49, 50, 52, 55]. In addition, even if the health insurance coverage (i.e. community based health insurance) was low and is limited to medical costs, we found protective effect of the scheme against CHE[57]. However, health insurance per se does not alleviate the major TB costs.

Study limitation and strengths

This study has some limitations. First, the cost measurements relied on patient's ability to remember, which increases risk of recall bias. However, we reduced the recall bias by interviewing participants who sought HIV and TB care within the past one month, when patients have a better recollection of their pathway to care[28, 31]. Second, HIV costs may be

For peer review only - http://bmjopen.bmj.com/site/about/guidelines.xhtml

For peer review only

overestimated when aggregated over one-year period. Third, our findings may not be representative of all patients with TB in Ethiopia, as the study is limited to specific regions of the country. Similarly, the PLHIV associations operate in high HIV prevalence urban areas, members of these associations may not be representative of both rural and non-members. Moreover, undetected HIV and TB cases not seeking care were not addressed. Despite these limitations, we used a standard tool and method to conduct the study. We believe that our study will be of high value to inform policy, at national and sub-national levels, related to financial risk protection of both diseases, which are central in achieving UHC.

To this end, despite OOP exemption of HIV and TB services in Ethiopia, we found that there is a large gap between the actual level of financial protection provided and the ideal goal. Our findings highlight several policy implications. First, more patient-centered care with effective diagnostics, appointment spacing for stable patients and community-based treatment are required to improve the delivery of HIV and TB services. Second, strategies are required to ensure social and financial risk protection for the households affected by HIV and TB. This requires effective integration of HIV and TB services with social and financial protection schemes, including the provision of travel vouchers, nutritional support and paid sick leaves through multi-sectoral collaboration.

CONCLUSION

HIV and TB affected individuals and their households in Ethiopia face substantial costs in seeking care despite “free medical services”. The incidence of CHE related to HIV and TB care were high in all income quintiles, though more so in the poorest households. Policy makers should introduce patient centred care; expand social and financial risk protection measures to minimize the high patient cost of HIV and TB care, particularly among vulnerable populations.

For peer review only - http://bmjopen.bmj.com/site/about/guidelines.xhtml

Supplementary file: Supplementary appendix 1: Consumption construction, and measurement of CHE, findings

Availability of data: The data supporting the conclusion of this study can be available upon reasonable request from the corresponding author.

Competing Interests: None declared

Funding: Bill & Melinda Gates Foundation grant number OPP1162384 supported this study.

Contributors: LFA, KAJ, MTT: conception and design of the study. LFA: acquisition of data, data analysis, and drafted the paper. LFA, AJ, KAJ, MTT, EKN: critically reviewed the paper and gave approval for the final version to be published.

Acknowledgments: The authors would like to thank all HIV and TB patients and supervisors who participated in this study. We thank all stakeholders, the Ministry of Health, Ethiopia, Ethiopian public health institute, Oromia and Afar regional health Bureau with their respective districts for making this research possible.

Figure 1. Pen's parade of household annual income/consumption gross and net of payments for HIV (a) and TB care (b) (Ethiopia).

Figure 2. Interval plot (with 95% confidence interval for the mean) of incidence and intensity of HIV and TB-related catastrophic health expenditures (CHE) across income quintiles, using 10% threshold (Ethiopia).

Figure 3. Concentrations curves and index (I) for direct, indirect, total cost and share of OOP to income/consumption for (a) HIV and (b) TB services in Ethiopia.

For peer review only

REFERENCES

1. Vitoria M, Granich R, Gilks CF, et al. The global fight against HIV/AIDS, tuberculosis, and malaria: current status and future perspectives. *American journal of clinical pathology*. 2009;131(6):844-8 DOI: 10.1309/ajcp5xhdb1pnaeyt.
2. Danforth K, Granich R, Wiedeman D, et al. Global Mortality and Morbidity of HIV/AIDS. 2017:29-44.
3. Bloom BR, Atun R, Cohen T, et al. Tuberculosis. 2017:233-313.
4. World Health Organization. Global tuberculosis report 2019. Geneva: World Health Organization; 2019.
5. World Health Organization. WHO fact sheet on HIV/AIDs. 25 July 2019. Available from: https://www.who.int/news-room/fact-sheets/detail/hiv-aids. Accessed 1 Sep 2019.
6. UNAIDS. UNAIDS data 2019. Available from https://www.unaids.org/en/resources/documents/2019/2019-UNAIDS-data. Accessed 24 Nov 2019.
7. WHO. IMPLEMENTING THE END TB STRATEGY: THE ESSENTIALS Geneva: World Health Organization; 2015.
8. Babigumira JB, Stergachis A, Veenstra DL, et al. Potential cost-effectiveness of universal access to modern contraceptives in Uganda. *PLoS One*. 2012;7(2):e30735 DOI: 10.1371/journal.pone.0030735.
9. The Federal Democratic Republic of Ethiopia Ministry of Health. Health Sector Transformation Plan 2015/16 - 2019/2020(2008-2012 EFY). Addis Ababa, Ethiopia.2015.
10. Federal Democratic Republic of Ethiopia Ministry of Health. September 2019.Ethiopia Health Accounts, 2016/17. Addis Ababa, Ethiopia.
11. Tanimura T, Jaramillo E, Weil D, et al. Financial burden for tuberculosis patients in low- and middle-income countries: a systematic review. *The European respiratory journal*. 2014;43(6):1763-75 DOI: 10.1183/09031936.00193413.
12. Russell S. The economic burden of illness for households in developing countries: a review of studies focusing on malaria, tuberculosis, and human immunodeficiency virus/acquired immunodeficiency syndrome. *The American journal of tropical medicine and hygiene*. 2004;71(2 Suppl):147-55.
13. Laurence YV, Griffiths UK, Vassall A. Costs to Health Services and the Patient of Treating Tuberculosis: A Systematic Literature Review. *PharmacoEconomics*. 2015;33(9):939-55 DOI: 10.1007/s40273-015-0279-6.
14. Wagstaff A, van Doorslaer E. Catastrophe and impoverishment in paying for health care: with applications to Vietnam 1993-1998. *Health economics*. 2003;12(11):921-34 DOI: 10.1002/hec.776.
15. O'Donnell O, Van Doorslaer E, Wagstaff A, et al. Analyzing Health Equity Using Household Survey Data A Guide to Techniques and Their Implementation. 2012.
16. Xu K, Evans DB, Kawabata K, et al. Household catastrophic health expenditure: a multicountry analysis. *Lancet (London, England)*. 2003;362(9378):111-7 DOI: 10.1016/s0140-6736(03)13861-5.
17. Kawabata K, Xu K, Carrin G. Preventing impoverishment through protection against catastrophic health expenditure. *Bulletin of the World Health Organization*. 2002;80(8):612.
18. Lopera MM, Einarson TR, Ivan Bula J. Out-of-pocket expenditures and coping strategies for people living with HIV: Bogota, Colombia, 2009. *AIDS care*. 2011;23(12):1602-8 DOI: 10.1080/09540121.2011.579938.
19. Barennes H, Frichittavong A, Gripenberg M, et al. Evidence of High Out of Pocket Spending for HIV Care Leading to Catastrophic Expenditure for Affected Patients in Lao People's Democratic Republic. *PLOS ONE*. 2015;10(9):e0136664 DOI: 10.1371/journal.pone.0136664.

For peer review only

20. Long Q, Smith H, Zhang T, et al. Patient medical costs for tuberculosis treatment and impact on adherence in China: a systematic review. *BMC Public Health*. 2011;11(1):393 DOI: 10.1186/1471-2458-11-393.
21. Wingfield T, Boccia D, Tovar M, et al. Defining catastrophic costs and comparing their importance for adverse tuberculosis outcome with multi-drug resistance: a prospective cohort study, Peru. *PLoS medicine*. 2014;11(7):e1001675 DOI: 10.1371/journal.pmed.1001675.
22. Asres A, Jerene D, Deressa W. Pre- and post-diagnosis costs of tuberculosis to patients on Directly Observed Treatment Short course in districts of southwestern Ethiopia: a longitudinal study. *Journal of health, population, and nutrition*. 2018;37(1):15 DOI: 10.1186/s41043-018-0146-0.
23. Vassall A, Seme A, Compernolle P, et al. Patient costs of accessing collaborative tuberculosis and human immunodeficiency virus interventions in Ethiopia. *The international journal of tuberculosis and lung disease : the official journal of the International Union against Tuberculosis and Lung Disease*. 2010;14(5):604-10.
24. Mauch V, Bonsu F, Gyapong M, et al. Free tuberculosis diagnosis and treatment are not enough: patient cost evidence from three continents. *The international journal of tuberculosis and lung disease : the official journal of the International Union against Tuberculosis and Lung Disease*. 2013;17(3):381-7 DOI: 10.5588/ijtld.12.0368.
25. Bhojani U, Thriveni B, Devadasan R, et al. Out-of-pocket healthcare payments on chronic conditions impoverish urban poor in Bangalore, India. *BMC Public Health*. 2012;12:990 DOI: 10.1186/1471-2458-12-990.
26. Ministry of Health, Ethiopia. Annual health sector performance report 2011 EFY (2018/19). Addis Ababa, Ethiopia.
27. World Health Organization. Health Financing: Monitoring Sustainable Development Goals— Indicator 3.8.2; World Health Organization: Geneva, Switzerland, 2019. Available from: https://www.who.int/health financing/topics/financial-protection/monitoring-sdg/en/. Accessed 5 Sep 2019.
28. Ukwaja KN, Alobu I, lgwenyi C, et al. The High Cost of Free Tuberculosis Services: Patient and Household Costs Associated with Tuberculosis Care in Ebonyi State, Nigeria. *PLOS ONE*. 2013;8(8):e73134 DOI: 10.1371/journal.pone.0073134.
29. Getahun B, Wubie M, Dejenu G, et al. Tuberculosis care strategies and their economic consequences for patients: the missing link to end tuberculosis. *Infectious Diseases of Poverty*. 2016;5(1):93 DOI: 10.1186/s40249-016-0187-9.
30. Ministry of Health, Ethiopia. Health Management Information System (HMIS) annual TB and Leprosy report 2016. Addis Ababa, Ethiopia.
31. WHO. WHO Global TB Programme. Protocol for survey to determine direct and indirect costs due to TB and to estimate proportion of TB-affected households experiencing catastrophic costs. November 2015.
32. Federal Democratic Republic of Ethiopia Ministry of Health. Health Service Utilization and Expenditure Survey among People Living with HIV (PLHIV), 2015/16, Addis Ababa, Ethiopia. August 2017.
33. National Bank of Ethiopia. Inter-bank daily foreign exchange rate in (USD)—archive. 2019. Available from: https://www.nbe.gov.et/market/dailyexchange.html. Accessed 29 Oct 2019.
34. International Monetary Fund.World Economic Outlook Database October 2015. Available from: https://www.imf.org/external/pubs/ft/weo/2019/02/weodata/index.aspx . Accessed 17 May 2019.
35. WHO Global TB Programme. Protocol for survey to determine direct and indirect costs due to TB and to estimate proportion of TB-affected households experiencing catastrophic costs. November 2015.

For peer review only

For peer review only - http://bmjopen.bmj.com/site/about/guidelines.xhtml

36. O'Donnell OvD, Eddy Wagstaff, Adam Lindelow, Magnus. Analyzing Health Equity Using Household Survey Data.
37. O'Donnell O, O'Neill S, Van Ourti T, et al. conindex: Estimation of concentration indices. *The Stata journal*. 2016;16(1):112-38.
38. L. Gortmaker S, Hosmer D, Lemeshow S. Applied Logistic Regression. *Contemporary Sociology*. 1994;23:159 DOI: 10.2307/2074954.
39. Boyer S, Abu-Zaineh M, Blanche J, et al. Does HIV services decentralization protect against the risk of catastrophic health expenditures?: some lessons from Cameroon. *Health services research*. 2011;46(6pt2):2029-56 DOI: 10.1111/j.1475-6773.2011.01312.x.
40. Poudel AN, Newlands D, Simkhada P. The economic burden of HIV/AIDS on individuals and households in Nepal: a quantitative study. *BMC health services research*. 2017;17(1):76 DOI: 10.1186/s12913-017-1976-y.
41. Mudzengi D, Sweeney S, Hippner P, et al. The patient costs of care for those with TB and HIV: a cross-sectional study from South Africa. *Health policy and planning*. 2017;32(suppl_4):iv48-iv56 DOI: 10.1093/heapol/czw183.
42. Etiaba E, Onwujekwe O, Torpey K, et al. What Is the Economic Burden of Subsidized HIV/AIDS Treatment Services on Patients in Nigeria and Is This Burden Catastrophic to Households? *PLoS One*. 2016;11(12):e0167117 DOI: 10.1371/journal.pone.0167117.
43. Onwujekwe O, Dike N, Chukwuka C, et al. Examining catastrophic costs and benefit incidence of subsidized antiretroviral treatment (ART) programme in south-east Nigeria. *Health policy (Amsterdam, Netherlands)*. 2009;90(2-3):223-9 DOI: 10.1016/j.healthpol.2008.10.006.
44. Shukla M, Agarwal M, Singh J, et al. Catastrophic health expenditure amongst people living with HIV/AIDs availing antiretroviral treatment services at two tertiary care health facilities in district of northern India. *National Journal of Community Medicine*. 2015;6:323-8.
45. Mnzava T, Mmari E, Berruti A. Drivers of Patient Costs in Accessing HIV/AIDS Services in Tanzania. *Journal of the International Association of Providers of AIDS Care*. 2018;17:2325958218774775 DOI: 10.1177/2325958218774775.
46. Foster N, Vassall A, Cleary S, et al. The economic burden of TB diagnosis and treatment in South Africa. *Social Science & Medicine*. 2015;12 DOI: 10.1016/j.socscimed.2015.01.046.
47. Datiko DG, Lindtjørn B. Cost and Cost-Effectiveness of Treating Smear-Positive Tuberculosis by Health Extension Workers in Ethiopia: An Ancillary Cost-Effectiveness Analysis of Community Randomized Trial. *PLOS ONE*. 2010;5(2):e9158 DOI: 10.1371/journal.pone.0009158.
48. Berhanu RH, Schnippel K, Kularatne R, et al. Can patients afford the cost of treatment for multidrug-resistant tuberculosis in Ethiopia? *The international journal of tuberculosis and lung disease : the official journal of the International Union against Tuberculosis and Lung Disease*. 2018;22(8):358-62 DOI: 10.5588/ijtld.17.0837.
49. Duan W, Zhang W, Wu C, et al. Extent and determinants of catastrophic health expenditure for tuberculosis care in Chongqing municipality, China: a cross-sectional study. *BMJ Open*. 2019;9(4):e026638 DOI: 10.1136/bmjopen-2018-026638.
50. Ukwaja KN, Alobu I, Abimbola S, et al. Household catastrophic payments for tuberculosis care in Nigeria: incidence, determinants, and policy implications for universal health coverage. *Infect Dis Poverty*. 2013;2(1):21 DOI: 10.1186/2049-9957-2-21.
51. Atif M, Sulaiman SA, Shafie AA, et al. Resource utilization pattern and cost of tuberculosis treatment from the provider and patient perspectives in the state of Penang, Malaysia. *BMC health services research*. 2014;14:353 DOI: 10.1186/1472-6963-14-353.
52. Laokri S, Dramaix-Wilmet M, Kassa F, et al. Assessing the economic burden of illness for tuberculosis patients in Benin: determinants and consequences of catastrophic health

For peer review only - http://bmjopen.bmj.com/site/about/guidelines.xhtml

expenditures and inequities. *Tropical medicine & international health : TM & IH*. 2014;19(10):1249-58 DOI: 10.1111/tmi.12365.

53. Viney K, Islam T, Hoa NB, et al. The Financial Burden of Tuberculosis for Patients in the Western-Pacific Region. *Tropical medicine and infectious disease*. 2019;4(2) DOI: 10.3390/tropicalmed4020094.
54. Pedrazzoli D, Siroka A, Boccia D, et al. How affordable is TB care? Findings from a nationwide TB patient cost survey in Ghana. *Tropical medicine & international health : TM & IH*. 2018;23(8):870-8 DOI: 10.1111/tmi.13085.
55. Prasanna T, Jeyashree K, Chinnakali P, et al. Catastrophic costs of tuberculosis care: a mixed methods study from Puducherry, India. *Global health action*. 2018;11(1):1477493 DOI: 10.1080/16549716.2018.1477493.
56. Tracking universal health coverage: 2017 global monitoring report. World Health Organization and International Bank for Reconstruction and Development / The World Bank; 2017. Licence: CC BY-NC-SA 3.0 IGO.
57. Zhou C, Long Q, Chen J, et al. The effect of NCMS on catastrophic health expenditure and impoverishment from tuberculosis care in China. *International Journal for Equity in Health*. 2016;15(1):172 DOI: 10.1186/s12939-016-0463-0.

For peer review only

For peer review only - http://bmjopen.bmj.com/site/about/guidelines.xhtml

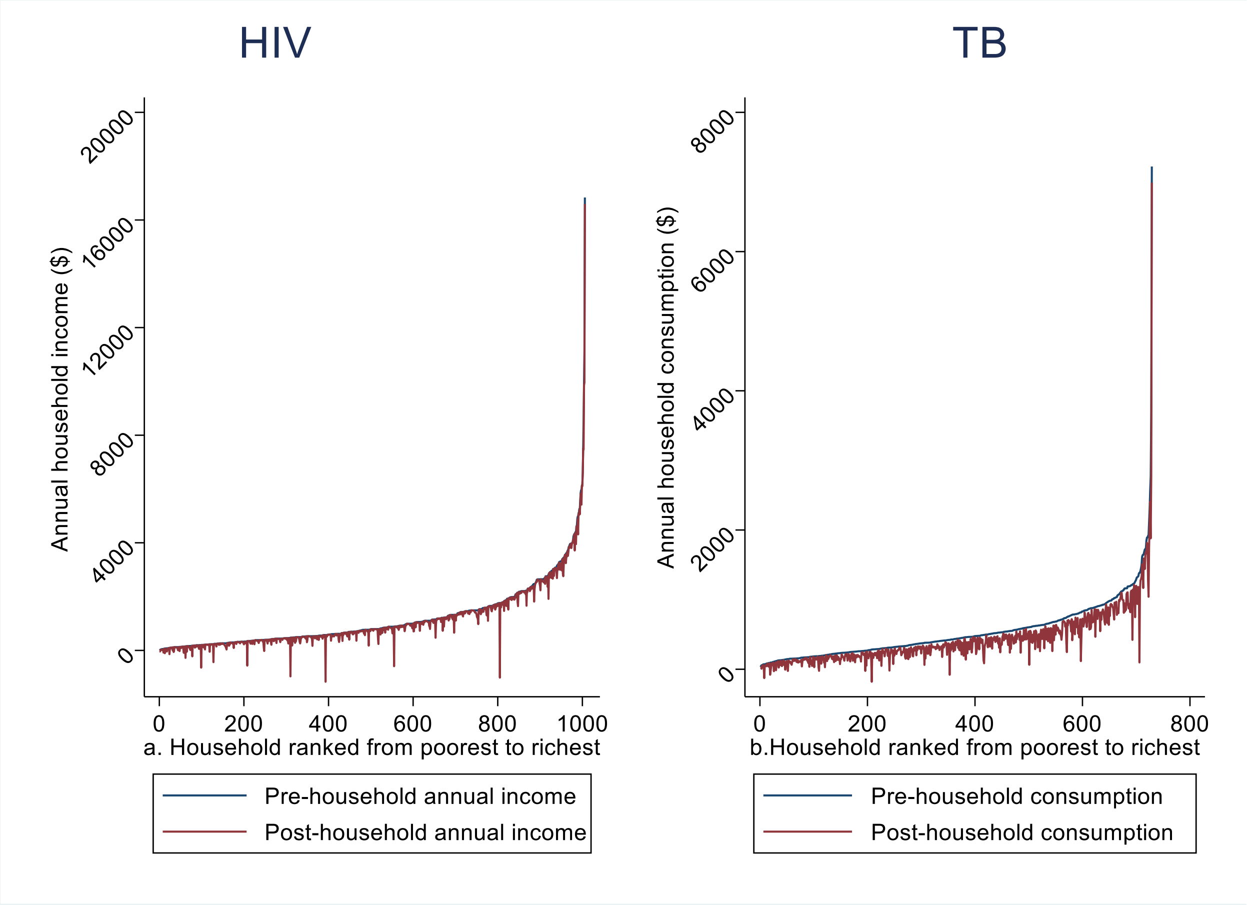

Figure 1. Pen's parade of household annual income/consumption gross and net of payments for HIV (a) and TB care (b) (Ethiopia).

For peer review only

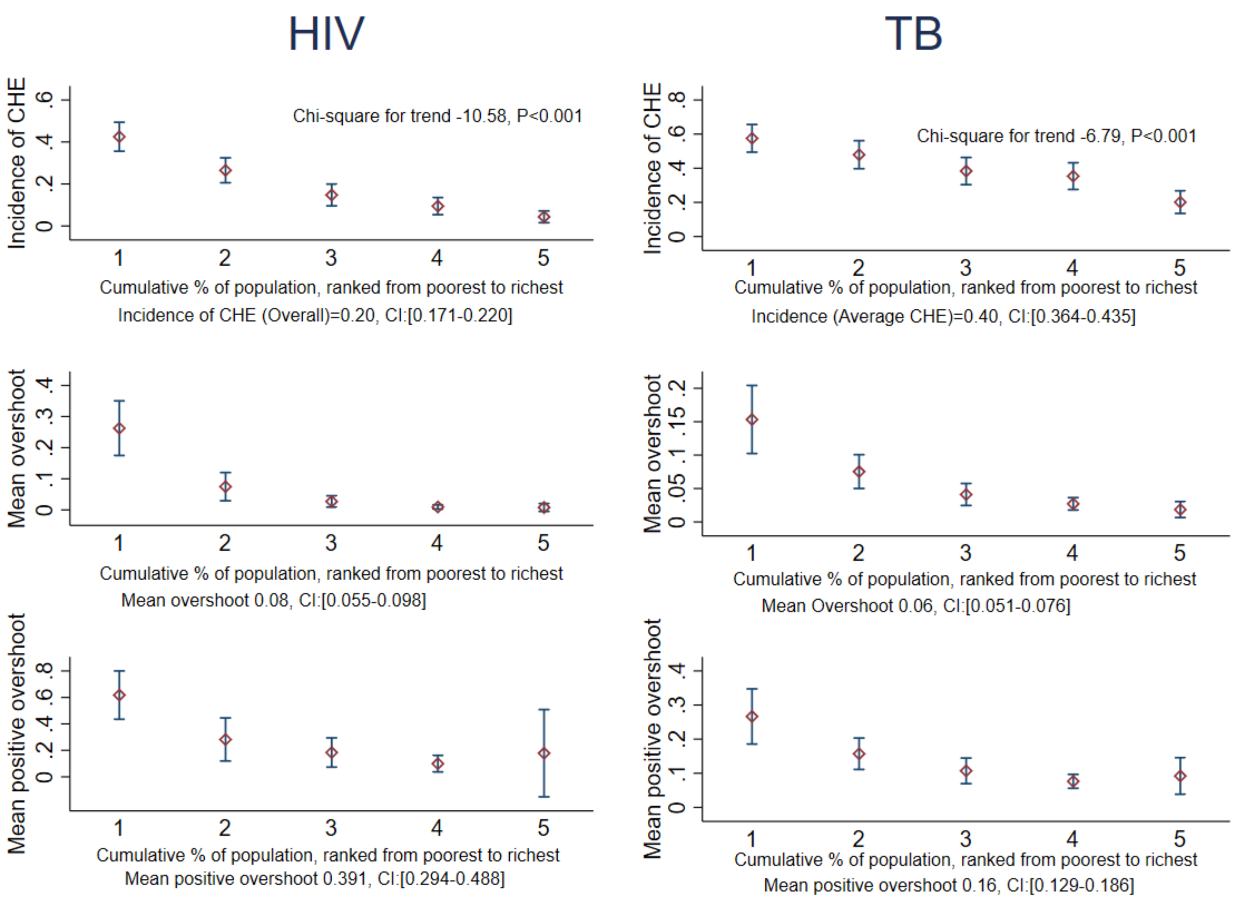

Figure 2. Interval plot (with 95% confidence interval for the mean) of incidence and intensity of HIV and TB-related catastrophic health expenditures (CHE) across income quintiles, using 10% threshold (Ethiopia).

For peer review only - http://bmjopen.bmj.com/site/about/guidelines.xhtml

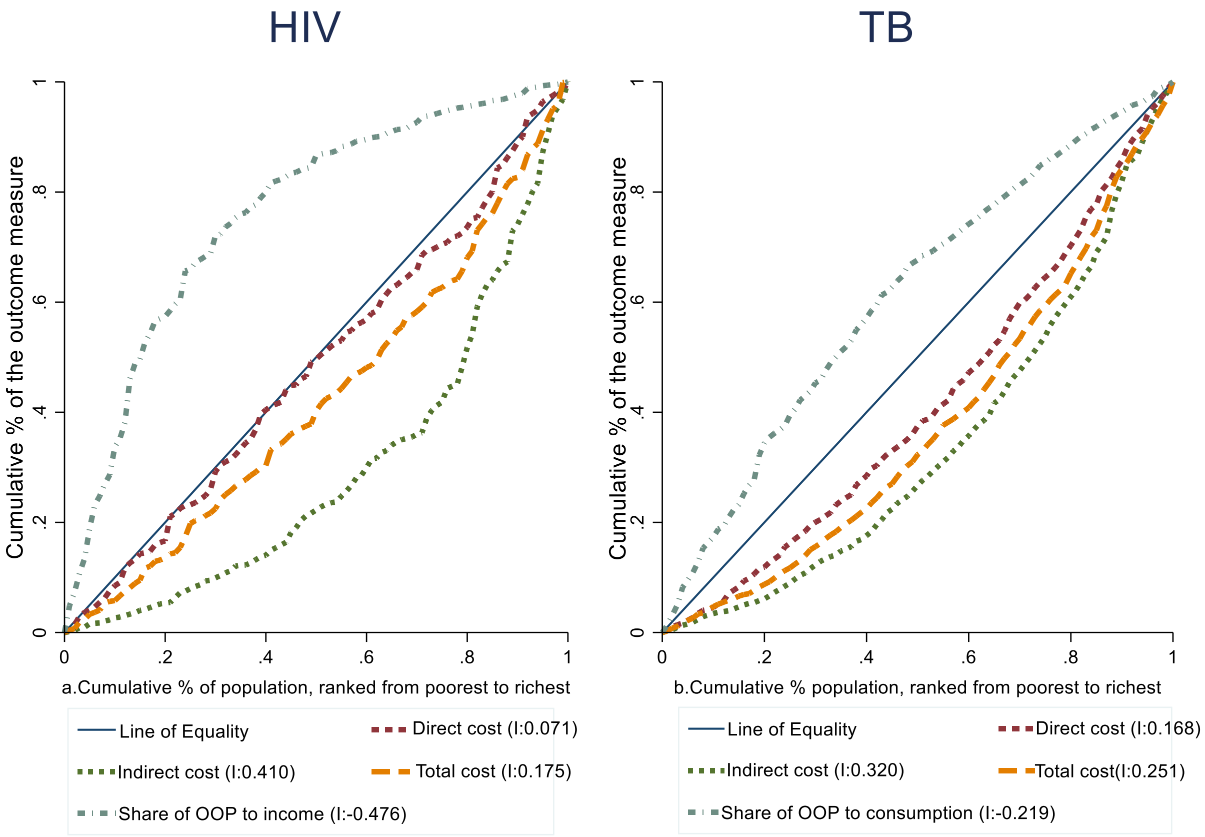

Figure 3. Concentrations curves and index (I) for direct, indirect, total cost and share of OOP to income/consumption for (a) HIV and (b) TB services in Ethiopia.

Supplementary appendix 1

The financial burden of HIV and TB among patients in Ethiopia: a cross-sectional survey

Lelisa Fekadu[1]*, Eyerusalem Negussie[2], Abdulrahman Jbaily[3], Mieraf Taddesse Tolla[3], Kjell Arne Johansson[1]

S1. Consumption construction

In this study, income (for HIV) and consumption as proxy for income (for tuberculosis) were used as household welfare measure. The consumption aggregates for tuberculosis (TB) was constructed using the overall value of the food (purchased food, home produced food) items, non-food items, and housing information collected at different recall periods (ranging from 1 to 12 months depending on how frequently the item purchased). Then, all reported expenditures on food items, non-food items, and housing were converted to a month period, then added up and multiplied by 12 to create annual consumption for each household.

The income (for HIV) and consumption (for TB) measures were also adjusted for family size and demographic composition to reflect economy of scale. We constructed per adult equivalence through dividing household income/consumption expenditure by an adult equivalent scale. Adult equivalent values (AE) were calculated using $AE = (A + \alpha K)^{\theta}$ for HIV, where A stands for number of adults, K is the number of children in the household, α is the cost of a child relative to an adult (0.33) and θ is the degree of economies of scale (0.9) (1). Whereas for TB, due to the unavailability of child data, we calculated using $AE = hsize^{\beta}$, where "$hsize$" is the actual household size and β was set to be 0.56 (2). Using per adult equivalence income (HIV) and consumption (TB) values, we grouped all households into five income quintiles of equal size.

For peer review only - http://bmjopen.bmj.com/site/about/guidelines.xhtml

S2. Incidence and intensity of catastrophic health expenditure (CHE)

S2.1. Measuring incidence of CHE (Headcount)

Catastrophic health expenditure (headcount, H) occurs when household OOP health spending exceeds a predefined threshold (10%) of household income/consumption. Furthermore, we conducted analysis at 20% threshold of both OOP and total cost (new definition of catastrophic cost recommended for TB), and at 40% of non-food expenditure (i.e. net of basic subsistence expenditure).

The CHE headcount is calculated using the equation

$$H = \frac{1}{N}\sum_{i=1}^{N} E_i \,,$$

$$\text{Where,} \quad E_i = 1 \text{ if } \frac{T_i}{X_i} \text{ or } \frac{T_i}{(X_i - FE_i)} > z \text{ and 0 otherwise.}$$

Here, N equals the total sample size, T_i is the OOP health spending of household i, X_i is the total expenditure of household i, FE_i is the food expenditure of household i and z is the specified threshold.

S2.2. Measuring intensity of CHE

The intensity is measured using overshoot (O) and mean positive overshoot (MPO).

S2.2.1. Overshoot

The overshoot measures the extent of average expenditure exceeding the given threshold in the entire sample. The overshoot is calculated using the equation

$$O = \frac{1}{N}\sum_{i=1}^{N} (E_i \left(\frac{Ti}{Xi}\right) - z)$$

S2.2.2. Mean positive overshoot

For peer review only - http://bmjopen.bmj.com/site/about/guidelines.xhtml

The mean positive overshoot measures the extent of an average expenditure exceeding the threshold among households experiencing CHE, and is calculated by:

$$MPO = \frac{O}{H}$$

Table A1. Mean (median) patient costs per TB case across three types of TB (Ethiopia) expressed in $.

Income quintiles	Type of TB					
	Pulmonary[¥]		Extra-pulmonary TB[¥]		Drug resistant-TB	
	Mean (SD)	Median (IQR)	Mean (SD)	Median (IQR)	Mean (SD)	Median (IQR)
**Poorest**	46 (43)	32 (13-62)	60 (45)	46 (32-75)	295 (290)	139 (65-607)
**Poor**	67 (44)	56 (34-85)	107 (90)	83 (49-136)	159 (85)	153 (68-199)
**Middle**	102 (80)	75 (48-137)	115 (74)	94 (72-131)	205 (175)	156 (86-250)
**Rich**	133 (99)	102 (65-176)	155 (86)	133 (79-212)	534 (671)	291 (131-669)
**Richest**	175 (165)	121 (78-216)	262 (226)	212 (112-294)	971 (1356)	610 (201-821)
**Total***	104 (107)	73 (41-135)	140 (138)	96 (58-180)	446 (732)	191 (107-607)

* kruskal wallis test p-value <0.001

¥ CHE at 10% threshold ranged from 33% to 57% for pulmonary and extra-pulmonary TB, respectively.

Table A2. Incidence and intensity of CHE for HIV and TB across different income quintiles and threshold, 2019, Ethiopia

Disease	Measure Of CHE	Threshold	Average	Income Quantile				
				Q1	Q2	Q3	Q4	Q5
**HIV**	Out-of-pocket HIV spending: as a share of annual income							
	Head count (CHE incidence) %	10%	20	43	27	15	10	4
	Overshoot %	10%	7.6	26.2	7.5	2.7	0.9	0.9
	Mean positive overshoot %	10%	39.1	61.8	28.2	18.3	9.9	17.8
	Out-of-pocket HIV spending: as a share of annual income							
	Head count (CHE incidence) %	20%	11	30	14	6	3	1
	Overshoot %	20%	6.2	22.5	5.6	1.7	0.5	0.6
	Mean positive overshoot %	20%	58.0	74.9	38.6	28.2	18.2	122.0
	Total HIV spending: as a share of annual income							
	Head count (CHE incidence) %	20%	15	38	18	11	6	3
	Overshoot %	20%	7.2	25.4	6.4	2.5	0.8	0.8
	Mean positive overshoot %	20%	48.6	67.8	36.2	23.6	15.0	27.7
	Out-of-pocket HIV spending: as a share of total non-food expenditure							
	Head count (CHE incidence) %	40%	11	31	15	7	3	1
	Overshoot %	40%	13.1	47.4	11.8	3.6	1.0	1.2
	Mean positive overshoot %	40%	116.3	153.0	79.5	55.4	39.7	128
**TB**	Out-of-pocket TB spending: as a share of annual income							
	Head count (CHE incidence) %	10%	40	58	48	38	35	20
	Overshoot %	10%	6.3	15.8	7.5	4.1	2.7	1.9
	Mean positive overshoot %	10%	15.3	26.6	15.7	10.7	7.6	9.2

For peer review only - http://bmjopen.bmj.com/site/about/guidelines.xhtml

	Out-of-pocket TB spending: as a share of annual income							
	Head count (CHE incidence) %	20%	17	38	23	10	8	6
	Overshoot %	20%	3.7	10.7	4.1	1.9	0.7	0.8
	Mean positive overshoot %	20%	21.3	27.8	17.7	18.7	8.8	15.2
	Total TB spending: as a share of annual income							
	Head count (CHE incidence) %	20%	48	60	53	48	46	36
	Overshoot %	20%	10.0	18.6	11.0	8.0	6.7	4.4
	Mean positive overshoot %	20%	20.0	31.1	20.9	16.7	14.7	12.3
	Out-of-pocket HIV spending: as a share of total non-food expenditure							
	Head count (CHE incidence) %	40%	37	50	43	39	33	19
	Overshoot %	40%	29.2	73.0	27.5	18.6	14.9	11.6
	Mean positive overshoot %	40%	78.7	146	63.6	47.7	44.7	59.5
	Where, Q1 = Poorest; Q2 = Poorer; Q3 = Middle; Q4 = Richer; Q5 = Richest.							

For peer review only - http://bmjopen.bmj.com/site/about/guidelines.xhtml

References

1. Deaton A, Zaidi S. Guidelines for constructing consumption aggregates for welfare analysis. 2002.
2. Xu K, Evans DB, Kawabata K, Zeramdini R, Klavus J, Murray CJ. Household catastrophic health expenditure: a multicountry analysis. Lancet (London, England). 2003;362(9378):111-7.

For peer review only

For peer review only - http://bmjopen.bmj.com/site/about/guidelines.xhtml
